# Evaluation of Online Near-Peer Teaching for Penultimate-Year Objective Structured Clinical Examinations in the COVID-19 Era: Longitudinal Study

**DOI:** 10.2196/37872

**Published:** 2022-05-26

**Authors:** Savan Shah

**Affiliations:** 1 Barking Havering and Redbridge University Hospitals NHS Trust Queen's Hospital London United Kingdom

**Keywords:** near-peer teaching, peer-assisted learning, Objective Structured Clinical Examination, OSCE, online teaching, COVID-19, medical education, learning, medical school, near-peer teacher, NPT, near-peer learner, NPL

## Abstract

**Background:**

The benefits of near-peer learning are well established in several aspects of undergraduate medical education including preparing students for Objective Structured Clinical Examinations (OSCEs). The COVID-19 pandemic has resulted in a paradigm shift to predominantly online teaching.

**Objective:**

This study aims to demonstrate the feasibility and benefits of an exclusively online near-peer OSCE teaching program in a time of significant face-to-face and senior-led teaching shortage.

**Methods:**

A teaching program was delivered to penultimate-year students by final-year students at Manchester Medical School. Program development involved compiling a list of salient topics and seeking senior faculty approval. Teachers and students were recruited on Facebook. In total, 22 sessions and 42 talks were attended by 72 students and taught by 13 teachers over a 3-month period. Data collection involved anonymous weekly questionnaires and 2 separate anonymous student and teacher postcourse questionnaires including both quantitative and qualitative components.

**Results:**

On a scale of 1-10, students rated the quality of the program highly (mean 9.30, SD 1.15) and felt the sessions were highly useful in guiding their revision (mean 8.95, SD 0.94). There was a significant increase in perceived confidence ratings after delivery of the program (*P*<.001). Teachers felt the program helped them better understand and retain the subject material taught (mean 9.36, SD 0.81) and develop skills to become effective clinical teachers (mean 9.27, SD 0.79).

**Conclusions:**

This is the first study demonstrating the efficacy of a near-peer OSCE teaching program delivered exclusively online. This provides an exemplary framework for how similar programs should be encouraged given their efficacy and logistical viability in supplementing the undergraduate curriculum.

## Introduction

“Near-peer teaching” refers to a way of teaching where the teacher is a trainee who is at least 1 year senior to the student and on the same level of the medical education spectrum [1]. Advocates fundamentally claim that its effectiveness stems from the social and cognitive congruence between near-peer learners (NPLs) and near-peer teachers (NPTs) as they are of similar ages, and therefore, share similar social roles (social congruence) and knowledge base (cognitive congruence) [2]. This cognitive congruence equips NPTs with unique insights and greater appreciation of the knowledge held by NPLs, subsequently enabling them to tailor the teaching to an appropriate level.

Near-peer teaching has grown in popularity in recent years within medical schools as a means of supplementing the formal curriculum. This paradigm shift was perhaps to be expected, given the sheer volume of literature advocating its benefits and effectiveness. For instance, Rodrigues et al [3] report on the efficacy of a near-peer teaching scheme delivered by junior doctors to medical students in a randomized controlled trial. The students ultimately made significantly fewer prescribing errors than the control group. In line with the social congruence theory, Leeper et al [4] argue that near-peer teaching creates a safer learning environment whereby students are more open to making mistakes and learning from them, while Topping [5] proposes that NPTs serve as influential role models in a phenomenon referred to as peer modeling. This helps students navigate the “hidden curriculum”—a set of unwritten rules students should follow to excel. The 19th century French moralist Joseph Joubert famously stated, “to teach is to learn twice,” eloquently conveying one of the key benefits of near-peer teaching. Elaborating on this, a randomized trial by Bargh and Schul [6] demonstrated that students who were asked to study a text with the task of teaching other students about it scored higher in an unexpected written test than those students from the control group who were asked to study for a test on their own. This suggests that teaching and its preceding preparation serves as a powerful drive for learning in a way that is distinct from preparing for an assessment. Others have taken a more pragmatic approach in accounting for the popularity of near-peer teaching, claiming it alleviates teaching pressures on faculty as medical school class sizes grow with a rising demand for doctors globally [7]. Therefore, it is reasonable to postulate that such near-peer programs will continue to grow in popularity.

Since the World Health Organization declared COVID-19 a global pandemic, widespread global lockdown restrictions resulted in a shift to online learning and cancellation of most face-to-face teaching and assessments [8]. Even prior to the pandemic, online learning had become an increasingly valued component of the undergraduate curriculum with multiple studies highlighting its merits. For instance, a recent meta-analysis found that online learning results in significantly better knowledge and skills outcomes based on posttest scores compared to traditional “offline” classroom teaching [9]. Another contemporaneous review of virtual teaching during the pandemic reports on the value of peer learning in this setting [10]. It highlights that peer learning reduces learner stress, helps develop resilience, and provides a platform for critical thinking and collaboration. Additional practical perceived benefits have been reported by medical students during the pandemic, including an absence of travel to attend sessions, flexibility to learn at their own pace, and opportunities to ask questions anonymously, subsequently encouraging wider engagement [11]. Nevertheless, numerous shortcomings of online learning have also been reported, including distraction from the physical environment, participants speaking over each other, and less of a perceived obligation to participate [12].

In this paper, I aim to outline how an online near-peer Objective Structured Clinical Examination (OSCE) teaching program can enhance the preparedness, knowledge, and skills of both the student and the teacher. In doing so, I aim to demonstrate that such endeavors are practical, reproducible, easy to implement, and valuable adjuncts to the medical undergraduate curriculum.

## Methods

### Ethics Approval

Consultation with the University of Manchester’s ethics decision tool [13] revealed that no formal external ethical approval was required for this study. All administered questionnaires outlined how the data gathered would be used and handled, followed by an opportunity to consent to the terms.

### Program Development and Delivery

Manchester Medical Society page facilitated advertisement of the program to prospective NPTs and NPLs. NPTs expressing interest were added to a group chat, while NPLs followed a link to join a Facebook page. An instructional sheet was sent to all the NPTs. It incorporated reminders to include disclaimers, keep the presentations under 30 minutes, have them completed by a particular date before being sent for quality assurance by the head of clinical teaching, and include an OSCE practice scenario at the end of their presentations to contextualize the taught content. This ensured session standardization and OSCE relevance.

The fourth-year syllabus at Manchester Medical School is split into 2 distinct overarching themes: “families and children” (F&C) and “mind and movement” (M&M). The programs, therefore, consisted of 2 separate weekly, hour-long sessions on Zoom, 1 for each theme. Each session was split into 2 separate 30-minute lessons delivered by 2 NPTs. A total of 42 talks were delivered over 3 months, covering a range of topics within each theme (Table 1). Presentations were reviewed and returned each weekend by the head of clinical teaching so that timely revisions could be made. Formal approval of the teaching program involved drafting a proposal and liaising with multiple members of the senior faculty at Manchester Medical School. Examples of 2 presentations can be seen in Multimedia Appendices 1 and 2.

**Table 1:** List of topics covered over the course of the program.

Week number	F&C^a^ topics	M&M^b^ topics
Week 1	Developmental milestones Gynecology history taking and menorrhagia	Delirium Mental state examination
Week 2	Breast medicine: triple assessment Pediatric examinations	Weakness Loss of vision
Week 3	Psychiatric history taking Common ear presentations and examination	Pediatric history taking Dermatology: common presentations and their assessment
Week 4	Oncological emergencies Infectious disease: a brief overview	Parkinson disease and examination Osteoarthritis, rheumatoid arthritis, and hand examination
Week 5	Cancer red flags Pediatric respiratory presentations and clinical assessment	Prescribing Stroke medicine
Week 6	Cervical health HIV	Ethics and law Gout
Week 7	Infertility Skin cancers	Vasculitis Psychopharmacology
Week 8	Pregnant abdomen examination and complications in pregnancy Pediatric gastroenterology and abdominal examination	Falls Common fractures
Week 9	Bleeding in early pregnancy Ethics and law	Alcohol dependence Pediatric orthopedics and examination
Week 10	Sexually transmitted infections and PV discharge Nonaccidental injury and safeguarding	Dementia Cancelled
Week 11	OSCE^c^ example stations Cancelled	SBAR^d^ handover OSCE example stations

^a^F&C: families and children.

^b^M&M: mind and movement.

^c^OSCE: Objective Structured Clinical Examination.

^d^SBAR: Situation, Background, Assessment, Recommendation.

### Feedback Questionnaires and Analysis

Anonymized weekly feedback questionnaires, 1 for the M&M and 1 for the F&C theme, were distributed using Google Forms following each session. This functioned as a means of tailoring future sessions toward the needs and wishes of the NPLs.

In addition, 2 separate postcourse questionnaires were distributed to NPLs and NPTs at the end of the program (Multimedia Appendices 3 and 4). The NPL postcourse questionnaire covered a range of questions relating to the perceived overall quality of the sessions, usefulness of the program, usefulness in guiding revision, confidence ratings prior to and after the program (out of 10), and the likelihood of organizing a similar teaching program. A free-text section allowed NPLs to relay any positive comments and advice for future improvement. The NPT postcourse evaluative questionnaire comprised of multiple statements with which the NPTs indicated their level of agreement on a 10-point Likert scale. They included topics relating to enjoyment of the program, its effect on teaching skills, and its benefits for teacher learning. The questionnaire concluded with 2 free-text sections inquiring about the greatest perceived benefit of engaging in the program and providing space for further comments.

A paired 2-tailed *t* test analysis compared confidence ratings of NPLs prior to and after the course. The remaining items in the NPL and NPT postcourse questionnaires were analyzed by mean and SD.

Two NPTs conducted qualitative analysis of the free-text sections in the NPL and NPT postcourse questionnaires. This involved identifying and agreeing on common themes and categorizing each response appropriately.

## Results

### Program Development and Delivery

The 22 sessions (11 for F&C and M&M each) were attended by a total of 72 different NPLs and taught by 13 NPTs. The F&C weekly session attendance ranged from 8-26 NPLs (mean 14.64, SD 4.41). The M&M weekly session attendance ranged from 11-26 NPLs (mean 14.45, SD 4.63). Overall weekly attendance across the 2 themes ranged from 21-49 NPLs (mean 29.09, SD 8.64).

### Feedback Questionnaires and Analysis

In total, 37 NPL postcourse evaluative questionnaires were completed equating to a 51.39% feedback response rate. A paired 2-tailed *t* test analysis demonstrated a statistically significant increase in mean confidence ratings following delivery of the program (*t*_36_=–13.71, *P*<.001). Quality of teaching, usefulness of the OSCE scenarios, and usefulness in guiding revision were all rated highly (Table 2).

There was considerable variation in likelihood of organizing a similar program, with the majority taking a neutral stance (n=23), and the remaining indicating it is either somewhat likely or very likely (n=13), or it is somewhat unlikely (n=1). Of 37 students, 23 (62.16%) left comments in the free-text sections. Qualitative analysis revealed 8 broad themes with some overlap (Table 3).

Of 13 NPTs, 11 (84.62%) completed the NPT postcourse questionnaire. Respondents unanimously agreed that engaging with the program helped them better understand the material taught (mean 9.45, SD 0.86), facilitated long-term retention of the content (mean 9.36, SD 0.81), helped them develop their teaching skills (mean 9.00, SD 1), was rewarding and motivational in engaging in more teaching in the future (mean 9.27, SD 0.79), and was highly enjoyable (mean 9.30, SD 1.15) (Table 4).

Thematic analysis of the 2 free-text sections mostly reinforced findings from these rating questions. When asked what the NPTs felt the greatest benefit of the program was, 3 broad themes emerged, with some overlap (Table 5).

The “any positive comments/areas for improvement” section comprised of 3 comments expressing gratitude for organizing the program. A summary of the key findings from both questionnaires can be seen in Table 6.

**Table 2:** Findings from rating questions in the near-peer learner postcourse questionnaires.

Questions	Responses, mean (SD)
How confident did you feel about your OSCE^a^ examinations prior to these teaching sessions?	4.51 (1.41)
How confident do you feel about your OSCE examinations now?	8.24 (0.93)
How would you rate the overall quality of the sessions?	9.30 (1.15)
How would you rate the usefulness of the OSCE practice scenarios at the end of each session in consolidating your learning?	8.92 (0.95)
How useful did you find the sessions in guiding your revision?	8.95 (0.94)

^a^OSCE: Objective Structured Clinical Examination.

**Table 3:** Qualitative analysis of free-text sections in near-peer learner postcourse questionnaires in response to the question “Any positive comments/areas for improvement?”

Theme	Examples of comments	Responses, n
Thanks, or expressions of gratitude	“Thank you so much to all the speakers and organizers for the effort through the semester!”	8
Praising the quality, organization, or structure of the teaching	“Really liked how the sessions were structured with a [sic] OSCE scenario at the end to consolidate the topic being taught. Really great stuff.”	7
Helped in getting ahead with revision or placement	“Attending the sessions helped me get a head start on the placement I hadn’t attended yet as everything was pitched at the correct level and at a good pace.”	4
Advice to include more multiple-choice questions	“The only thing I could suggest to improve if [sic] more MCQ’s^a^ but I do understand that it was a more OSCE focused program which you guys hit the nail on the head. Thanks!”	3
Praising the top tips provided	“Really well organized and taught. You really provided some great OSCE tips throughout!”	3
Beneficial, given limited clinical exposure due to COVID-19	“The sessions have been particularly valuable with the reduced clinical opportunities we’ve had at placement with COVID.”	2
Request for more sessions	“Would love some more sessions in the new year–found this really helpful in consolidating the TCD^b^ cases.”	2
Connection or technical issues	“Although the technical issues did sometimes interfere the sessions have been a great addition to the online cases.”	2

^a^MCQ: multiple-choice question.

^b^TCD: Themed Case Discussion.

**Table 4:** Findings from rating questions in the near-peer teacher postcourse questionnaires.

Questions or statements	Responses, mean (SD)
Getting involved with the teaching has helped me better understand the subject material I taught.	9.45 (0.68)
Getting involved in the teaching has helped with my long-term retention of the subject material I taught.	9.36 (0.81)
I have found this teaching experience to be rewarding, and it has motivated me to get involved in more teaching in the future.	9.00 (1.00)
As a result of this experience, I have developed teaching skills that I will use in the future as a doctor.	9.27 (0.79)
On a scale of 1-10, how enjoyable did you find this teaching experience?	9.30 (1.15)

**Table 5:** Qualitative analysis of the free-text sections in the near-peer teacher postcourse questionnaires in response to the question “What do you think has been the greatest benefit of teaching in this program?”

Theme	Examples of comments	Responses, n
Beneficial to learning or guiding revision	“I think teaching is a very effective way of deepening your own understanding of a topic as well as identifying any gaps in your knowledge. If you are able to explain a complex topic as well as answer specific questions, this shows you have a very good level of knowledge. Therefore, this teaching program allows the tutors to expand on their understanding.”	6
Enhancing or developing teaching skills	“Helpful feedback form students which I have reflected on to improve my teaching skills.”	6
Increased self-confidence in teaching ability	“Over the course of the program, I’ve felt more confident and less nervous whilst teaching and ended up enjoying it a lot more than I would have expected!”	2

**Table 6:** Summary of the key findings from near-peer learner and near-peer teacher questionnaires.

Items in the questionnaires			Key findings
**Near-peer learner postcourse questionnaire**			
	Change in perceived confidence to sit the OSCE^a^ examination	Precourse: mean 4.51, SD 1.41 Postcourse: mean 8.24, SD 0.93 *P*<.001	
	“Any positive comments/areas for improvement?”	Thanks/expression of gratitude (n=8) Praising the quality/organization/structure of teaching (n=7)	
**Near-peer teacher postcourse questionnaire**			
	Getting involved with the teaching has helped me better understand the subject material I taught	Mean 9.45, SD 0.68	
	“What do you think has been the greatest benefit of teaching in this program?”	Beneficial to learning/revision (n=6) Enhancing/developing teaching skills (n=6)	

^a^OSCE: Objective Structured Clinical Examination.

## Discussion

### Principal Findings

In line with the existing body of literature, this study clearly demonstrates the value of undergraduate near-peer programs for both the student and the teacher. Crucially, it demonstrates a significant increase in NPLs’ perceived confidence to sit their OSCE examinations. Simulated OSCE scenarios and the setup of the sessions in helping guide revisions explain this finding. The study also demonstrates numerous benefits obtained by NPTs including enhanced comprehension and retention of the taught materials, development of teaching skills, and increased motivation in engaging with future teaching endeavors.

### Evaluation of Findings and Comparison to Prior Work

A variety of reasons can account for the significant increase (*P*<.001) in NPLs’ confidence in the OSCE performance. Evaluation of the free-text sections in the weekly questionnaires helped tailor future sessions suitably. For instance, the overwhelmingly positive reaction to the OSCE scenarios prompted organization of 2 sessions focusing exclusively on such scenarios for the final week of the program. The perceived benefit of these scenarios was mirrored in the NPL postcourse questionnaires’ average rating of usefulness of the scenarios (mean 8.92, SD 0.95); it also supports the notion that simulation exercises in medical education lead to improvements in knowledge, confidence, and procedural performance upon retesting [14].

Rashid et al [15] adopted a similar methodology in 2011 to evaluate the success of their near-peer program for final year OSCE examinations and similarly found their program to have a positive influence on OSCE preparedness. However, unlike prior comparable studies, this study ensured that all taught materials were quality assured by a relevant senior faculty—the head of clinical teaching and examinations at Manchester Medical School. The exceptionally high ratings of the overall quality (mean 9.30, SD 1.15) are likely attributable to session standardization, facilitated by quality assurance and NPTs’ instruction sheets. This highlights the importance of quality assurance and selection of appropriate individuals to ensure the relevance and accuracy of teaching. Qualitative analysis further consolidates this, with 7 students praising the overall quality, organization, or structure of the sessions. The anecdotal “top tips” provided throughout the sessions were also praised (n=3), further corroborating the notion of “cognitive congruence” and its intrinsic benefit in near-peer teaching [2].

NPTs also obtained multiple benefits, with engagement shown to enable better understanding (mean 9.45, SD 0.86) and long-term retention (mean 9.36, SD 0.81) of the taught material. These findings are supported by multiple prior studies [6,16] and analysis of the free-text sections, where a handful of NPTs (n=6) commented that teaching was beneficial to their learning and revision. Dandavino et al [17] postulate that other than learning, teaching provides the additional benefit of covert familiarization with teaching and learning principles, yielding a more effective learner and teacher. Additionally, the reported positive impact on teaching skills and motivation to teach as a doctor coincides with the United Kingdom’s General Medical Council’s “good medical practice” guidelines, which state that doctors “should be prepared to contribute to teaching and training doctors and students” [18]. The free-text sections complemented this notion, with NPTs outlining how teaching in an informal environment and responding to feedback helped enhance their teaching skills (n=6) and confidence in their teaching ability (n=2).

### Limitations and Future Directions

First, the data collected and interpreted from the questionnaires in this study mostly deal with perceptions and lack objective measures. Future endeavors will look to collect data from tangible objective outcomes (ie, achieved OSCE marks of NPLs). Second, connectivity issues occasionally interfered with the delivery of teaching and our ability to share media and video clips—a drawback also identified by Mageswaran and Ismail [19], and 2 of the students in this study (Table 3). This can be avoided by asking all teachers to host sessions from a location where a stable connection can be ensured and to record sessions for students to play back in their own time if their connection fails during the live session. Third, although this study argues the case for near-peer learning, it fails to compare it to the alternative (ie, teaching by experienced senior faculty). Nevertheless, numerous prior studies have established that students taught by peers perform just as well as those taught by experienced teachers [3,20]. For instance, a randomized controlled trial by Tolsgaard et al [20] in 2007 found that students taught catheterization and intravenous access by student teachers performed just as well as those taught by associated professors in a postcourse assessment.

### Conclusions

Numerous studies have established the value of the near-peer model in undergraduate medical education, including facilitation of OSCE preparedness [21,22]. However, this is the first study to evaluate an OSCE program delivered exclusively online. Given that the majority of curricular undergraduate clinical teaching is delivered by senior clinicians, there is scope for implementation of online OSCE-focused programs similar to the one described in this study. Such programs should involve careful review of weekly feedback to fine-tune sessions, practice scenarios to consolidate learning, and session standardization including quality assurance by relevant senior faculty as well as instructional sheets for teachers. Although online teaching is not a substitute to learning in the clinical setting, it is certainly a worthy adjunct that is economically and logistically viable and highly effective in preparing both students and teachers for clinical examinations and future practice. Peers embarking on similar endeavors will uncover novel ideas for delivering similar online programs in the future.

## Appendix

Multimedia Appendix 1 Pediatric milestones presentation.

Multimedia Appendix 2 Falls presentation.

Multimedia Appendix 3 Near-peer teacher postcourse questionnaire.

Multimedia Appendix 4 Near-peer learner postcourse questionnaire.

## Abbreviations

F&C: families and children
M&M: mind and movement
NPL: near-peer learner
NPT: near-peer teacher
OSCE: Objective Structured Clinical Examination
