# Supplementary material for: Evaluation of Online Near-Peer Teaching for Penultimate-Year Objective Structured Clinical Examinations in the COVID-19 Era: Longitudinal Study
Source: JMIR Med Educ. 2022 May 26;8(2):e37872. doi: 10.2196/37872 (PMC9185334; doi:10.2196/37872)
Supplement: Multimedia Appendix 1 [file mededu_v8i2e37872_app1.pptx]

## Slide 1
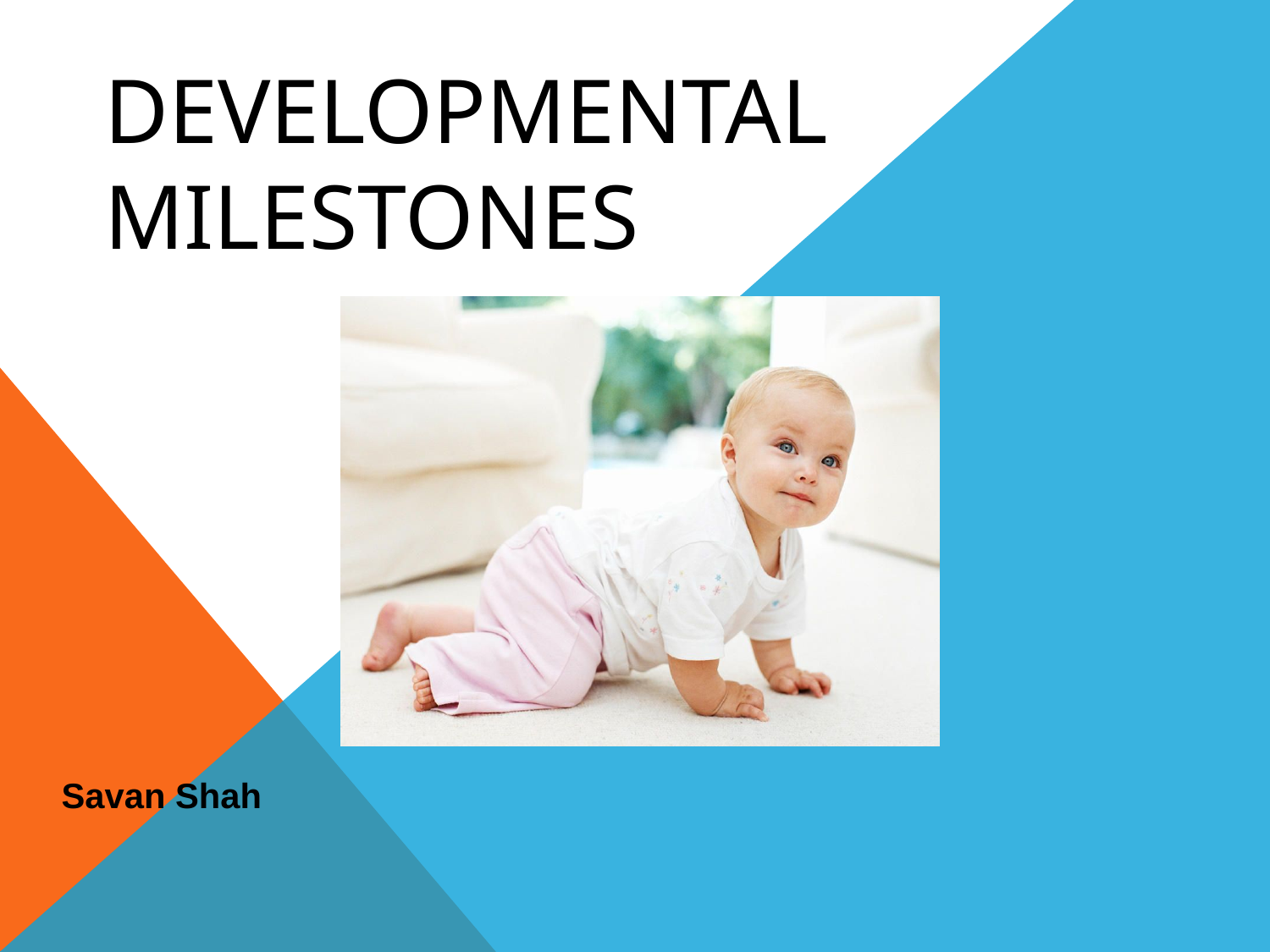

# Developmental MILESTONES
Savan Shah

## Slide 2
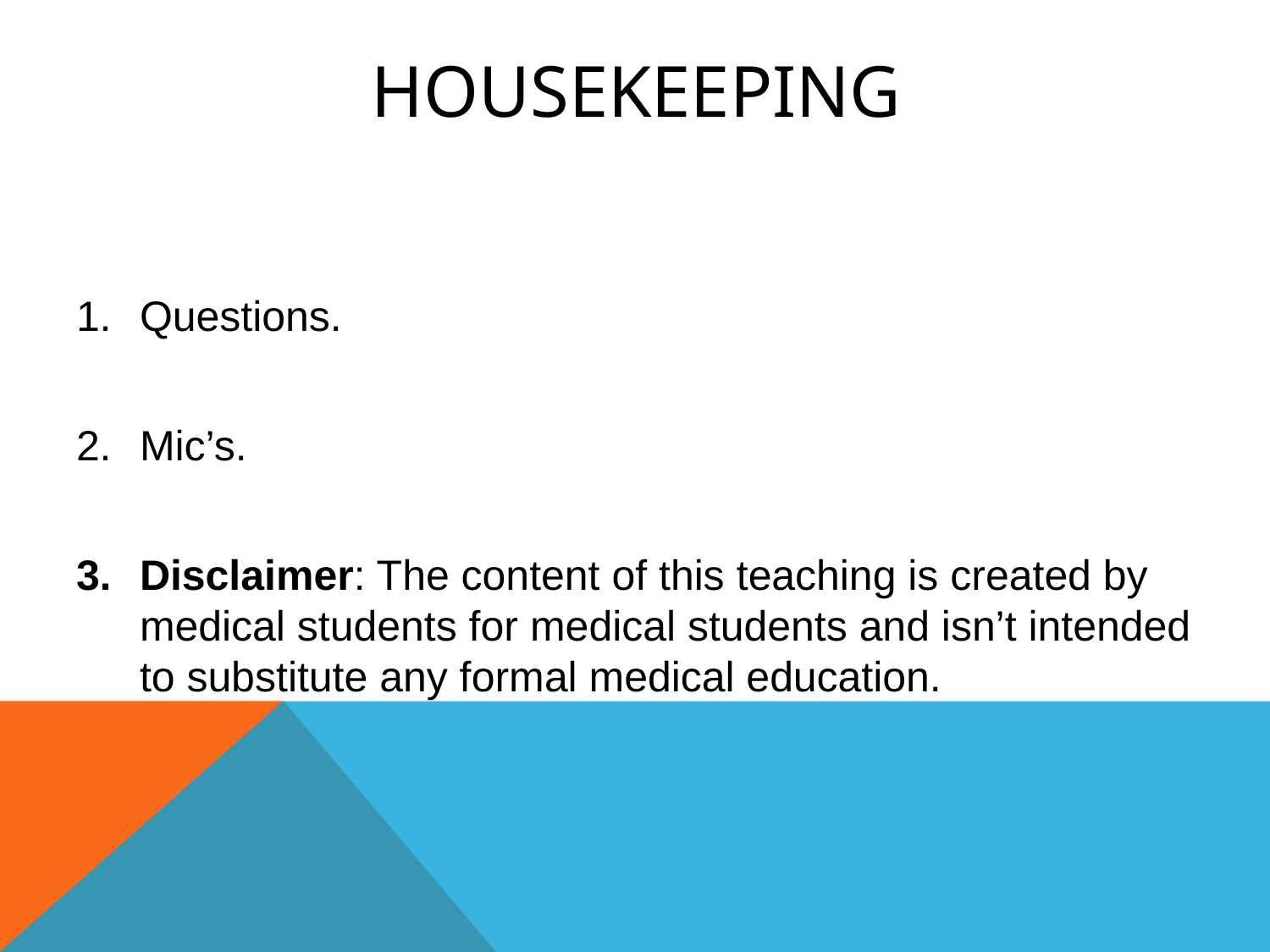

# Housekeeping
Questions.
Mic’s.
Disclaimer: The content of this teaching is created by medical students for medical students and isn’t intended to substitute any formal medical education.

## Slide 3
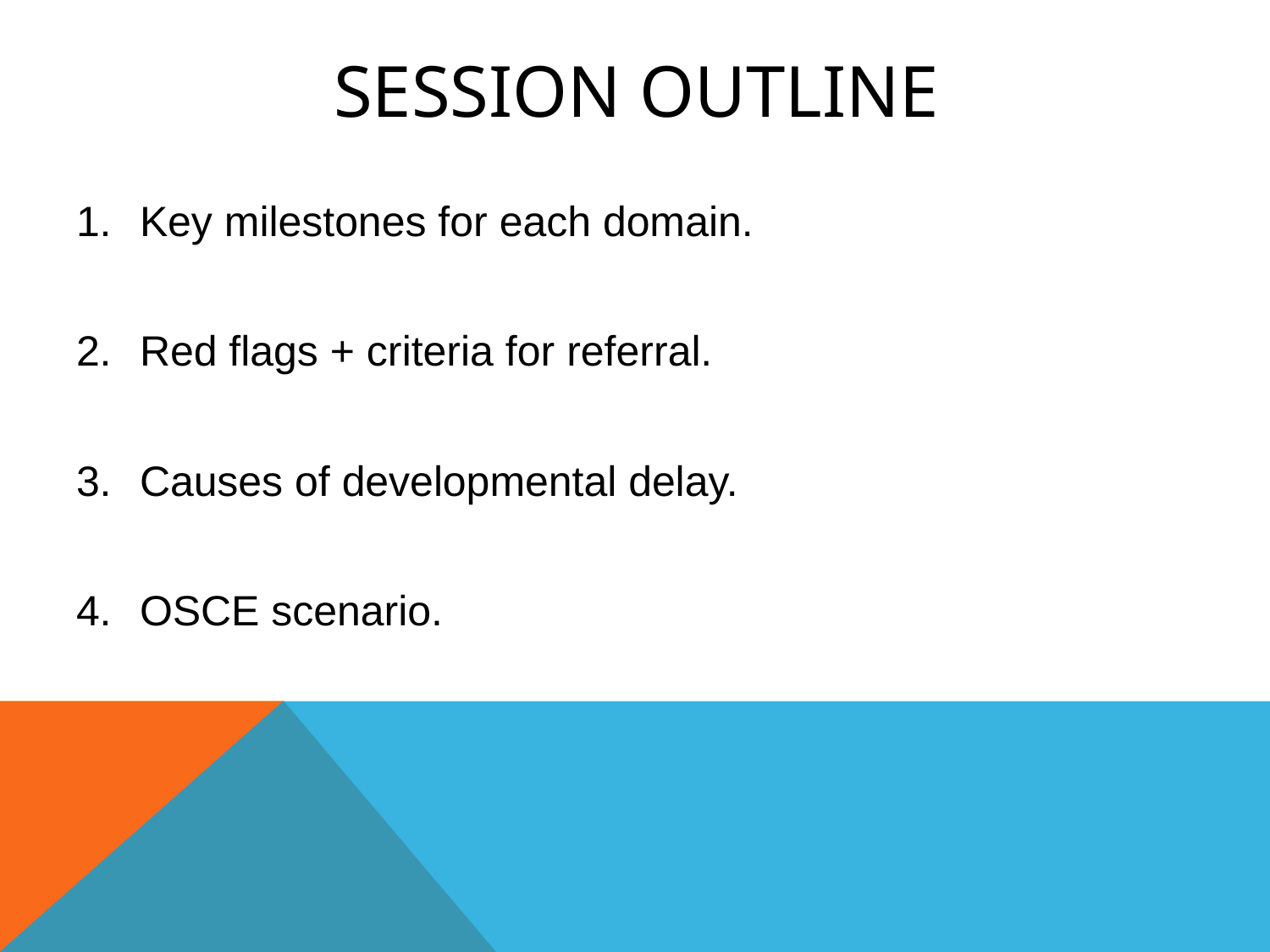

# Session outline
Key milestones for each domain.
Red flags + criteria for referral.
Causes of developmental delay.
OSCE scenario.

## Slide 4
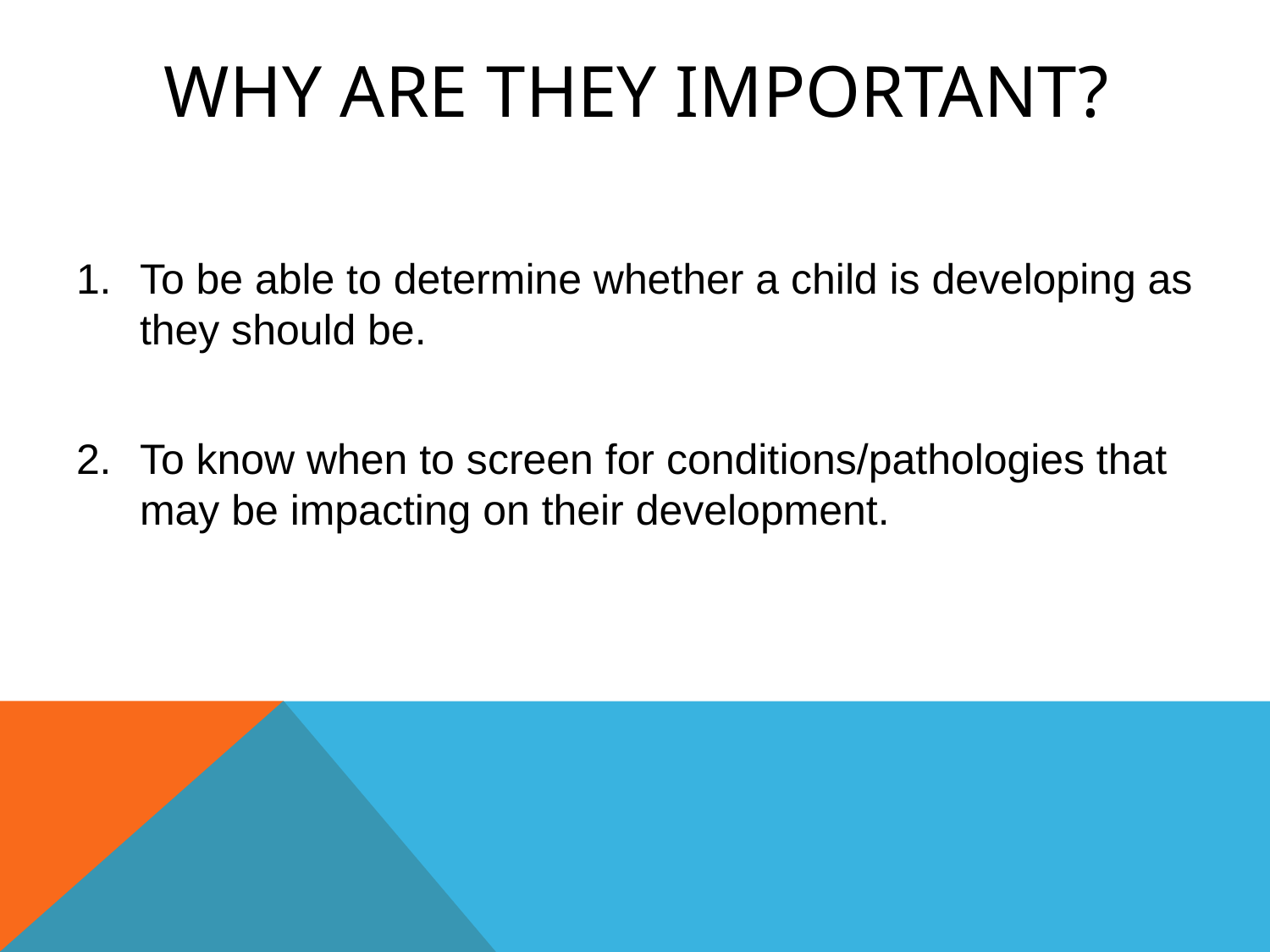

# Why are they important?
To be able to determine whether a child is developing as they should be.
To know when to screen for conditions/pathologies that may be impacting on their development.

## Slide 5
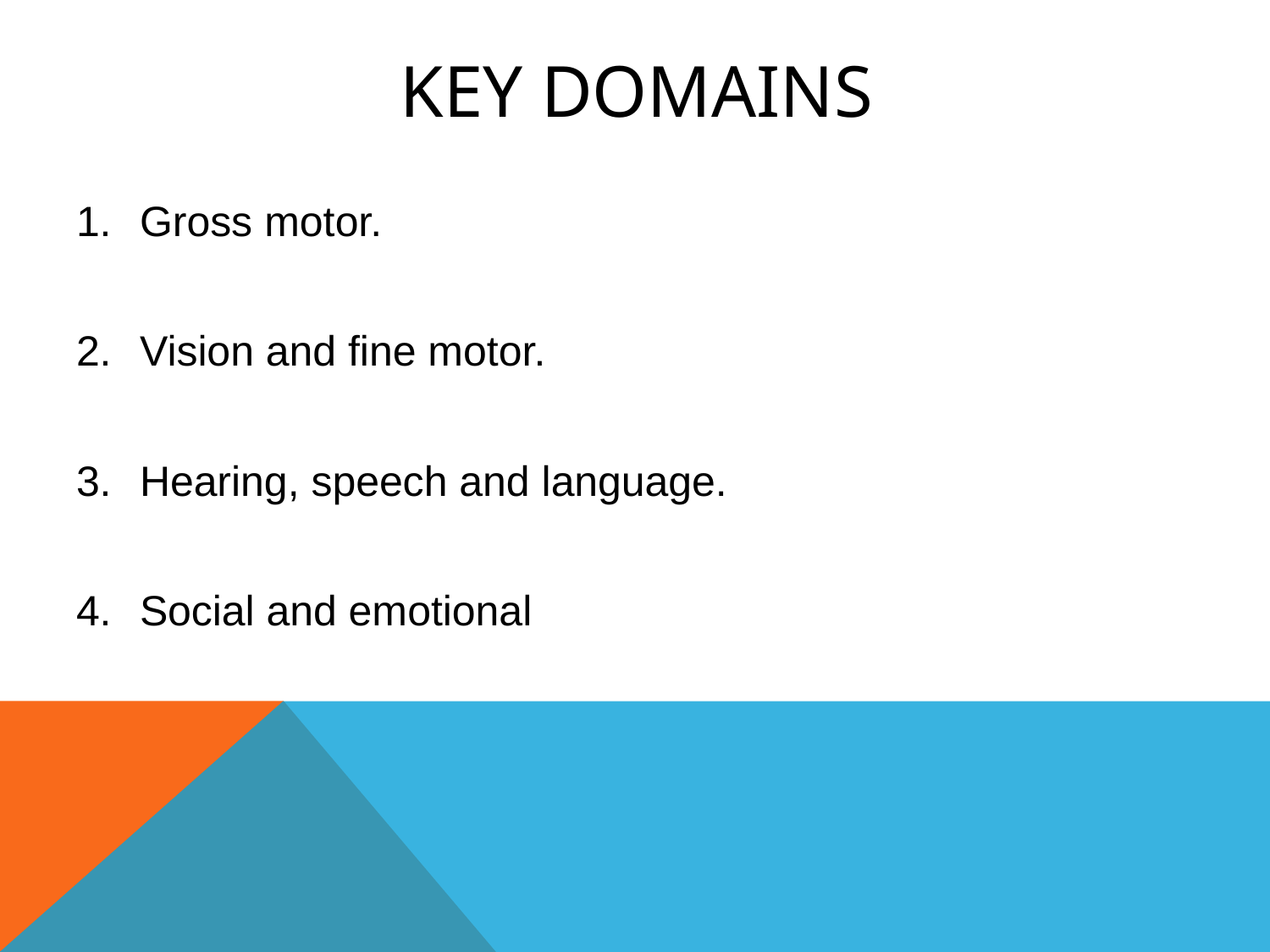

# Key domains
Gross motor.
Vision and fine motor.
Hearing, speech and language.
Social and emotional

## Slide 6
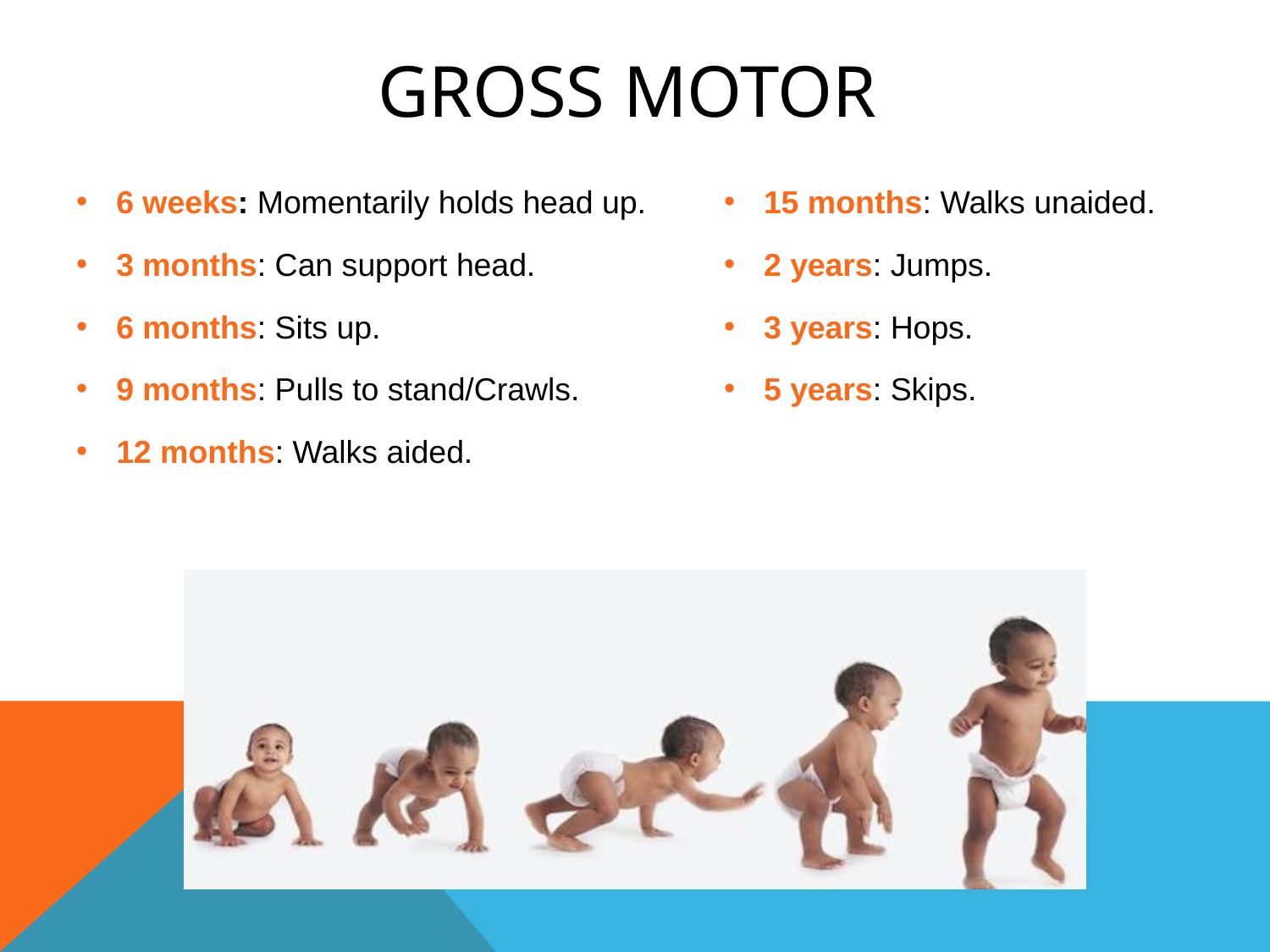

# Gross motor
15 months: Walks unaided.
2 years: Jumps.
3 years: Hops.
5 years: Skips.
6 weeks: Momentarily holds head up.
3 months: Can support head.
6 months: Sits up.
9 months: Pulls to stand/Crawls.
12 months: Walks aided.

## Slide 7
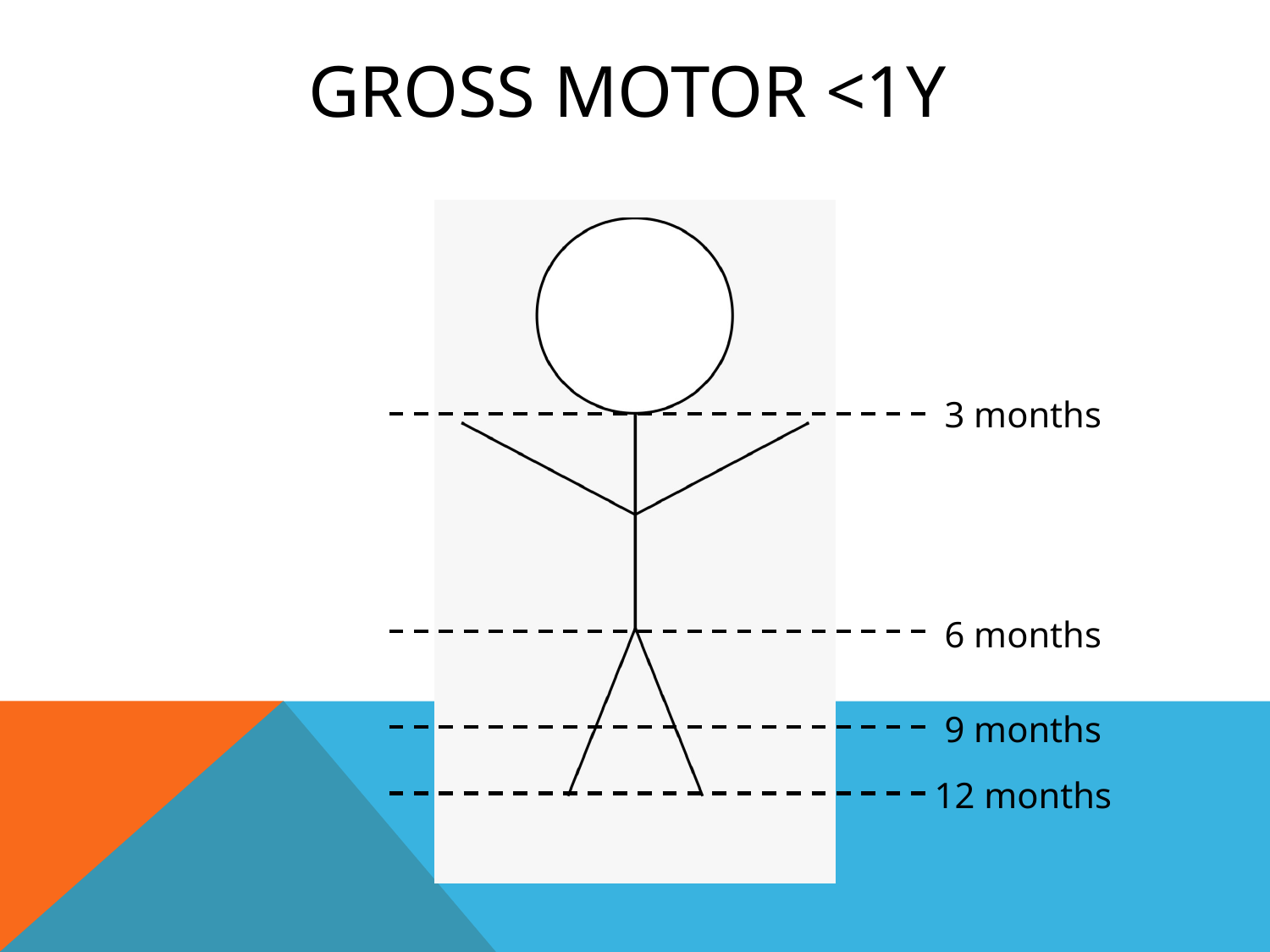

# Gross motor <1Y
3 months
6 months
9 months
12 months

## Slide 8
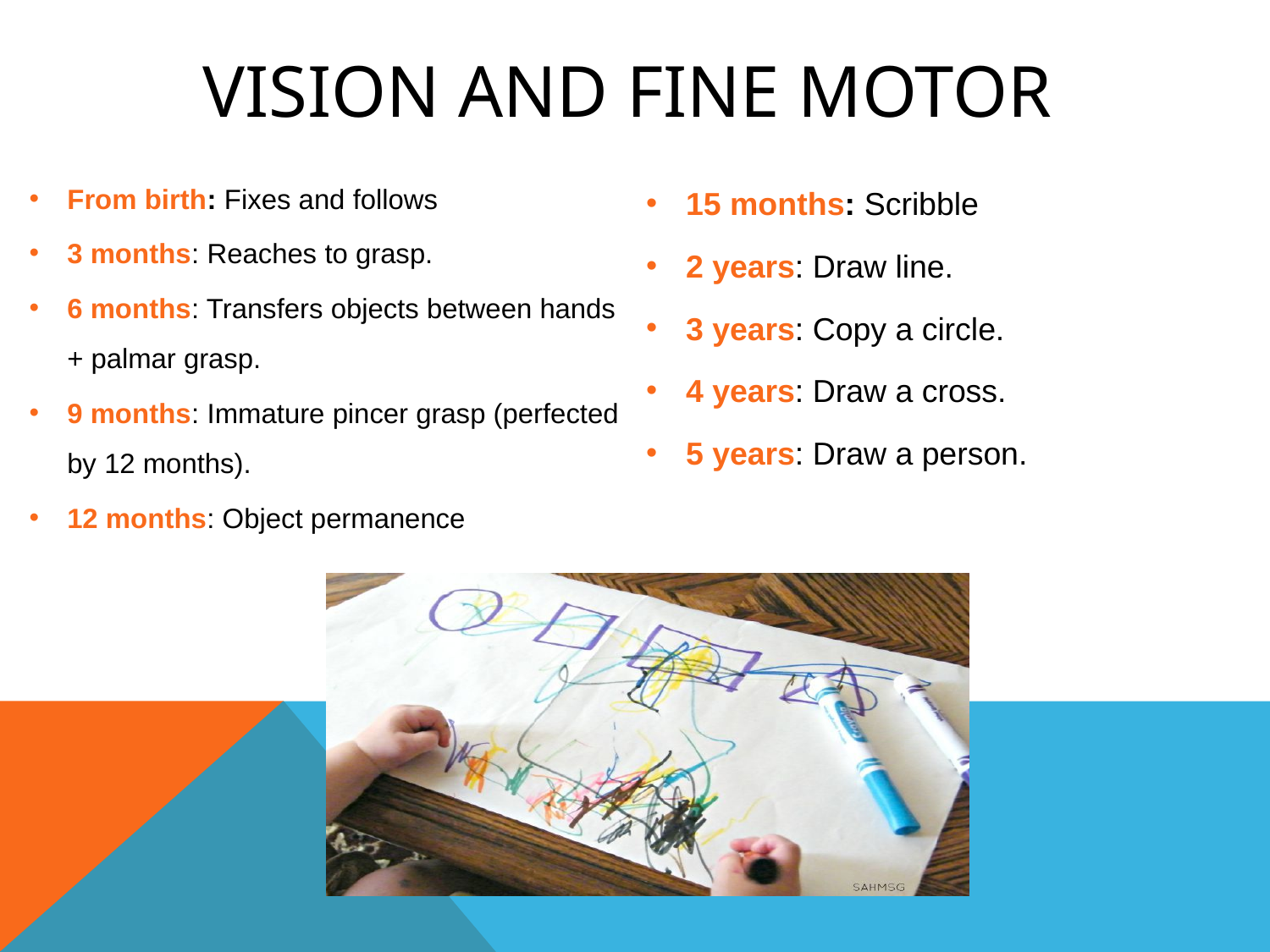

# Vision and fine motor
From birth: Fixes and follows
3 months: Reaches to grasp.
6 months: Transfers objects between hands + palmar grasp.
9 months: Immature pincer grasp (perfected by 12 months).
12 months: Object permanence
15 months: Scribble
2 years: Draw line.
3 years: Copy a circle.
4 years: Draw a cross.
5 years: Draw a person.

## Slide 9
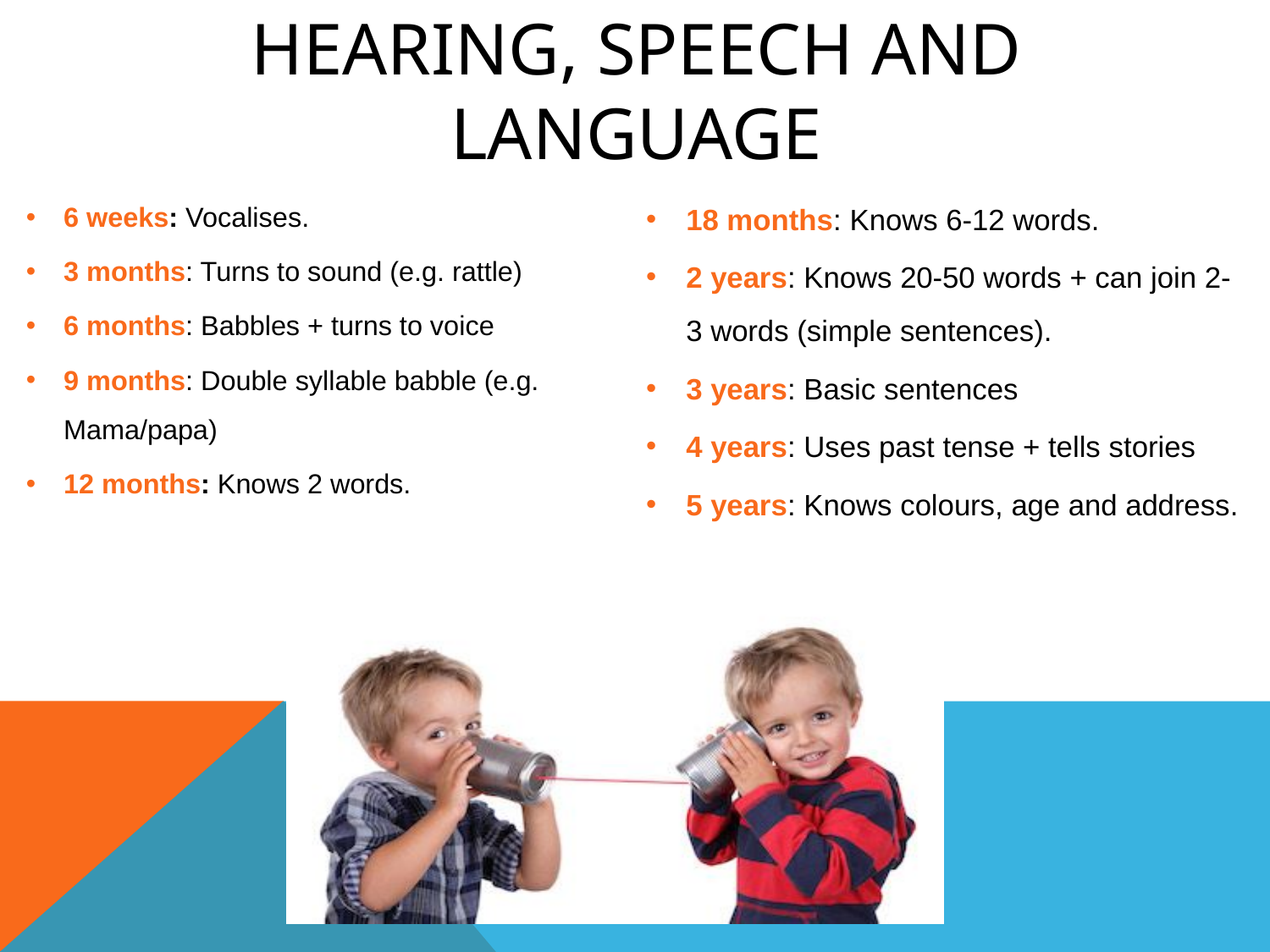

# Hearing, Speech and language
6 weeks: Vocalises.
3 months: Turns to sound (e.g. rattle)
6 months: Babbles + turns to voice
9 months: Double syllable babble (e.g. Mama/papa)
12 months: Knows 2 words.
18 months: Knows 6-12 words.
2 years: Knows 20-50 words + can join 2-3 words (simple sentences).
3 years: Basic sentences
4 years: Uses past tense + tells stories
5 years: Knows colours, age and address.

## Slide 10
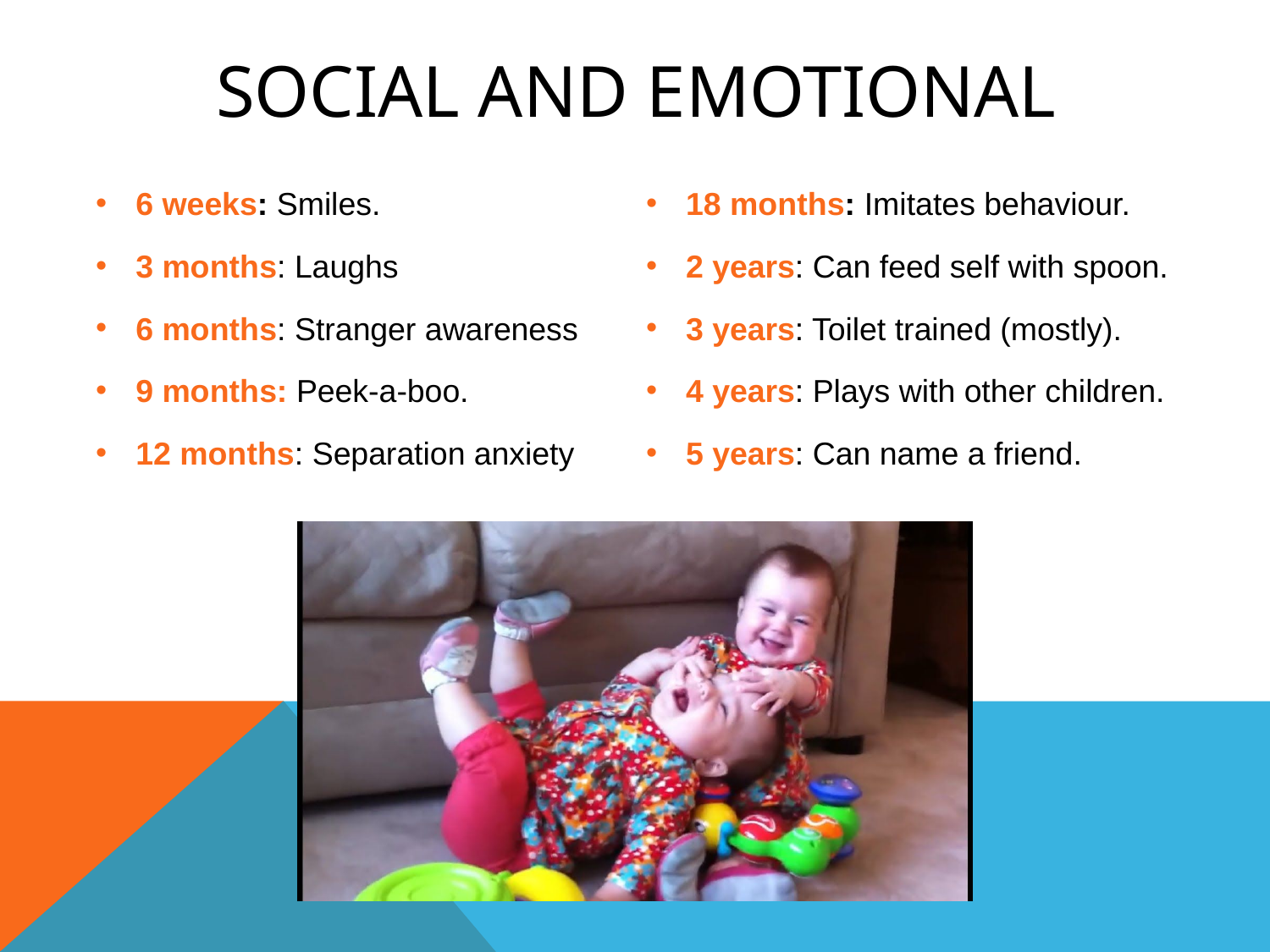

# Social and emotional
6 weeks: Smiles.
3 months: Laughs
6 months: Stranger awareness
9 months: Peek-a-boo.
12 months: Separation anxiety
18 months: Imitates behaviour.
2 years: Can feed self with spoon.
3 years: Toilet trained (mostly).
4 years: Plays with other children.
5 years: Can name a friend.

## Slide 11
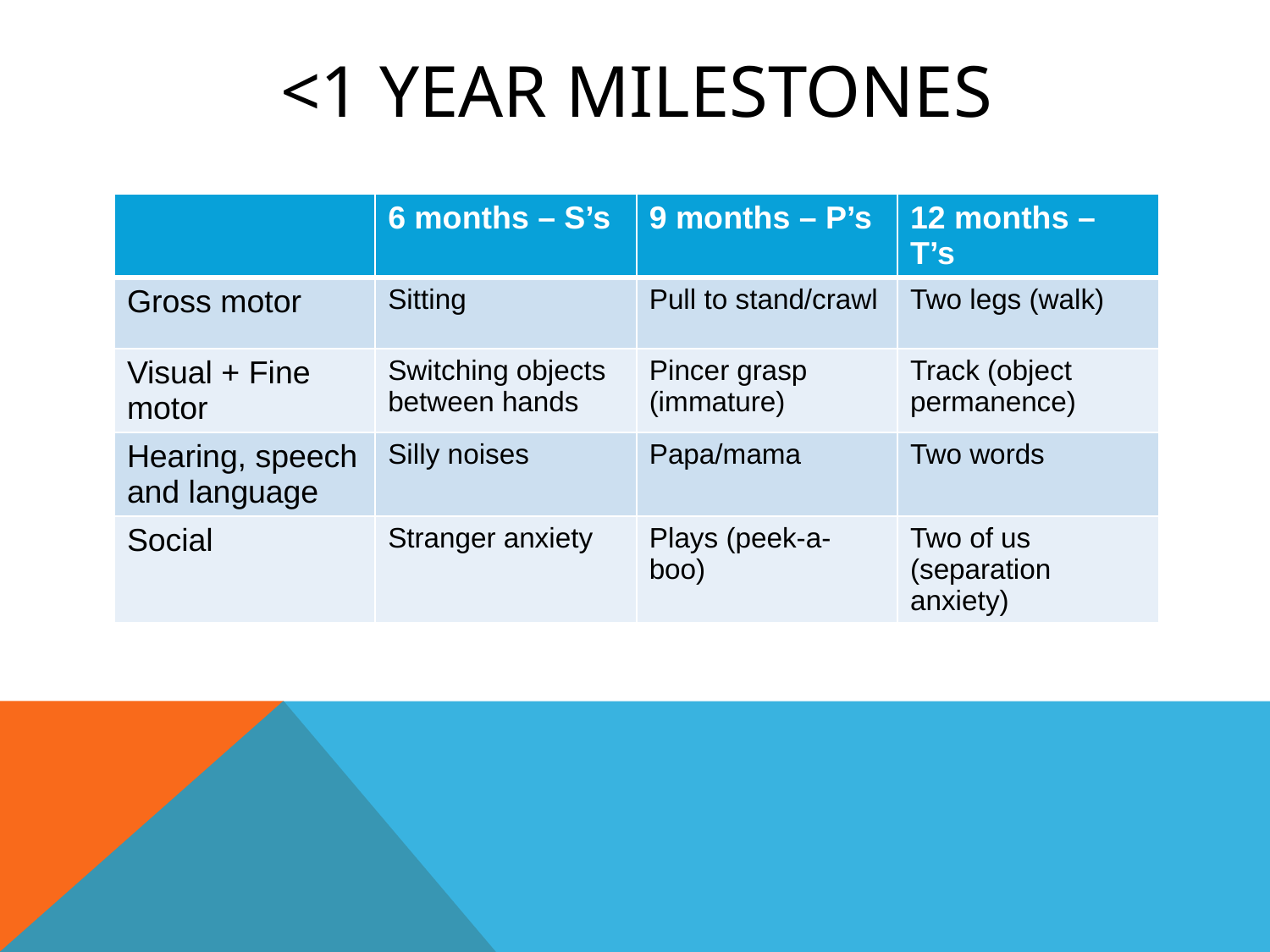

# <1 YEAR MILESTONES
| | 6 months – S’s | 9 months – P’s | 12 months – T’s |
| --- | --- | --- | --- |
| Gross motor | Sitting | Pull to stand/crawl | Two legs (walk) |
| Visual + Fine motor | Switching objects between hands | Pincer grasp (immature) | Track (object permanence) |
| Hearing, speech and language | Silly noises | Papa/mama | Two words |
| Social | Stranger anxiety | Plays (peek-a-boo) | Two of us (separation anxiety) |

## Slide 12
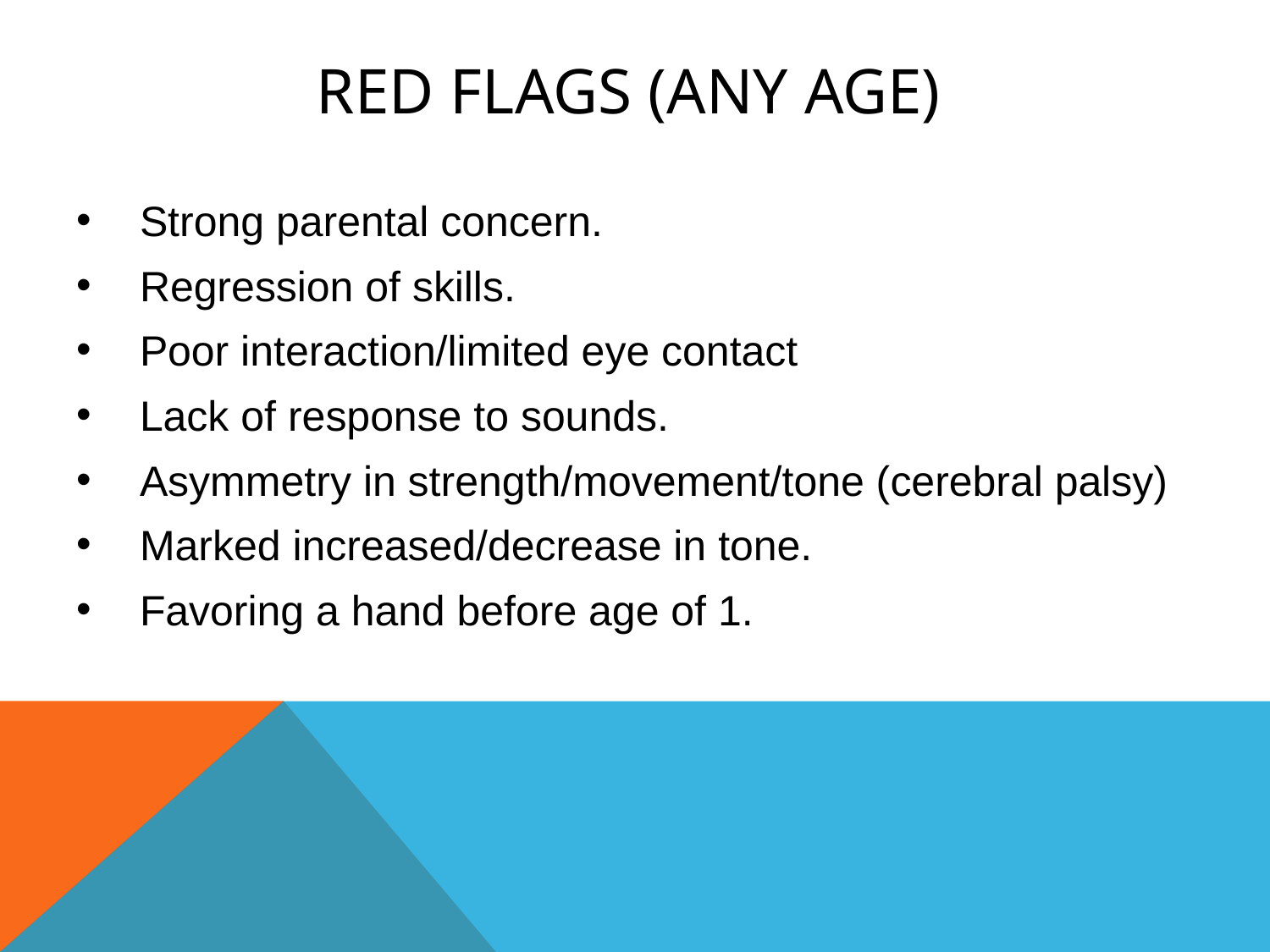

# Red flags (any age)
Strong parental concern.
Regression of skills.
Poor interaction/limited eye contact
Lack of response to sounds.
Asymmetry in strength/movement/tone (cerebral palsy)
Marked increased/decrease in tone.
Favoring a hand before age of 1.

## Slide 13
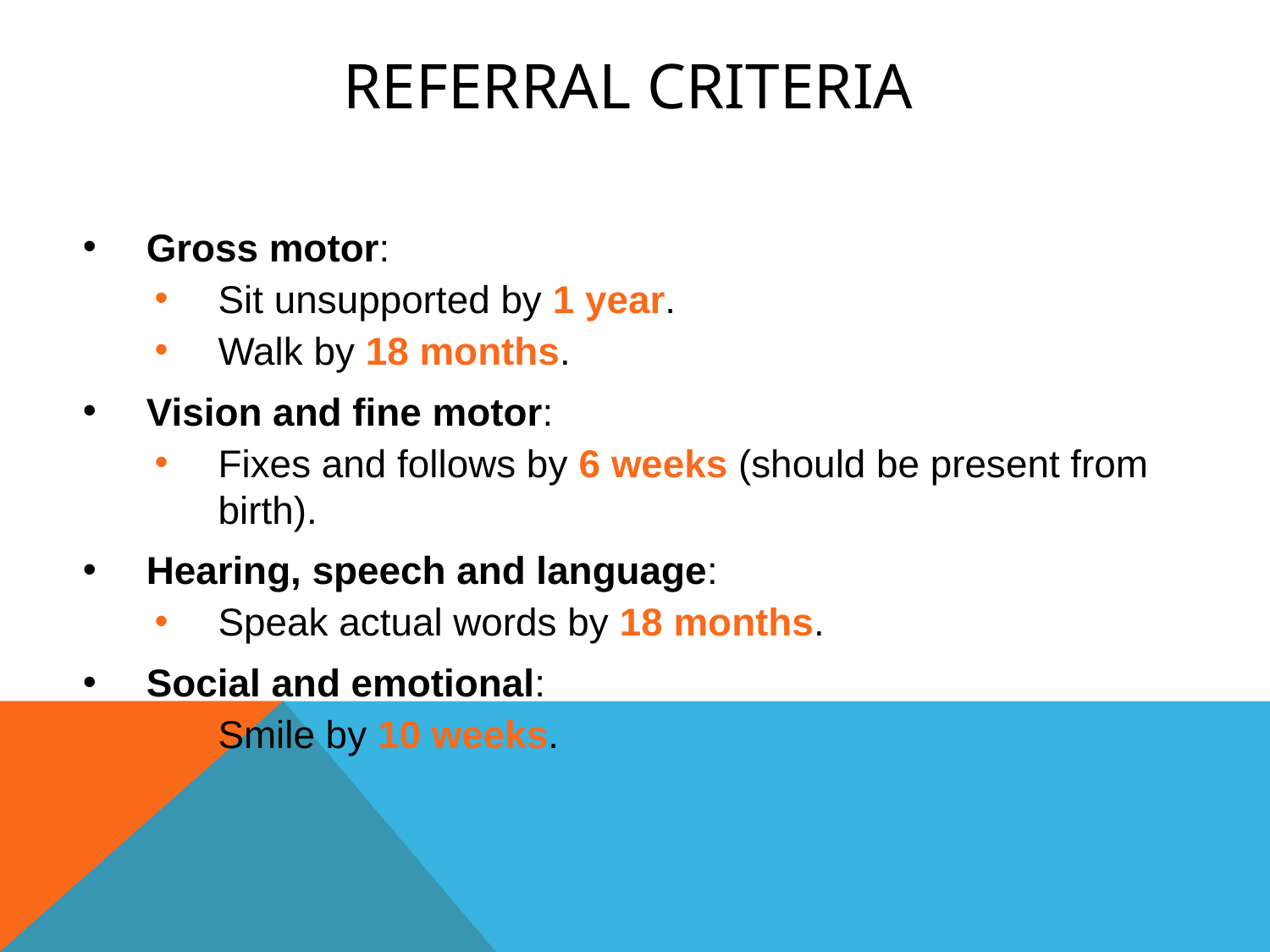

# Referral criteria
Gross motor:
Sit unsupported by 1 year.
Walk by 18 months.
Vision and fine motor:
Fixes and follows by 6 weeks (should be present from birth).
Hearing, speech and language:
Speak actual words by 18 months.
Social and emotional:
Smile by 10 weeks.

## Slide 14
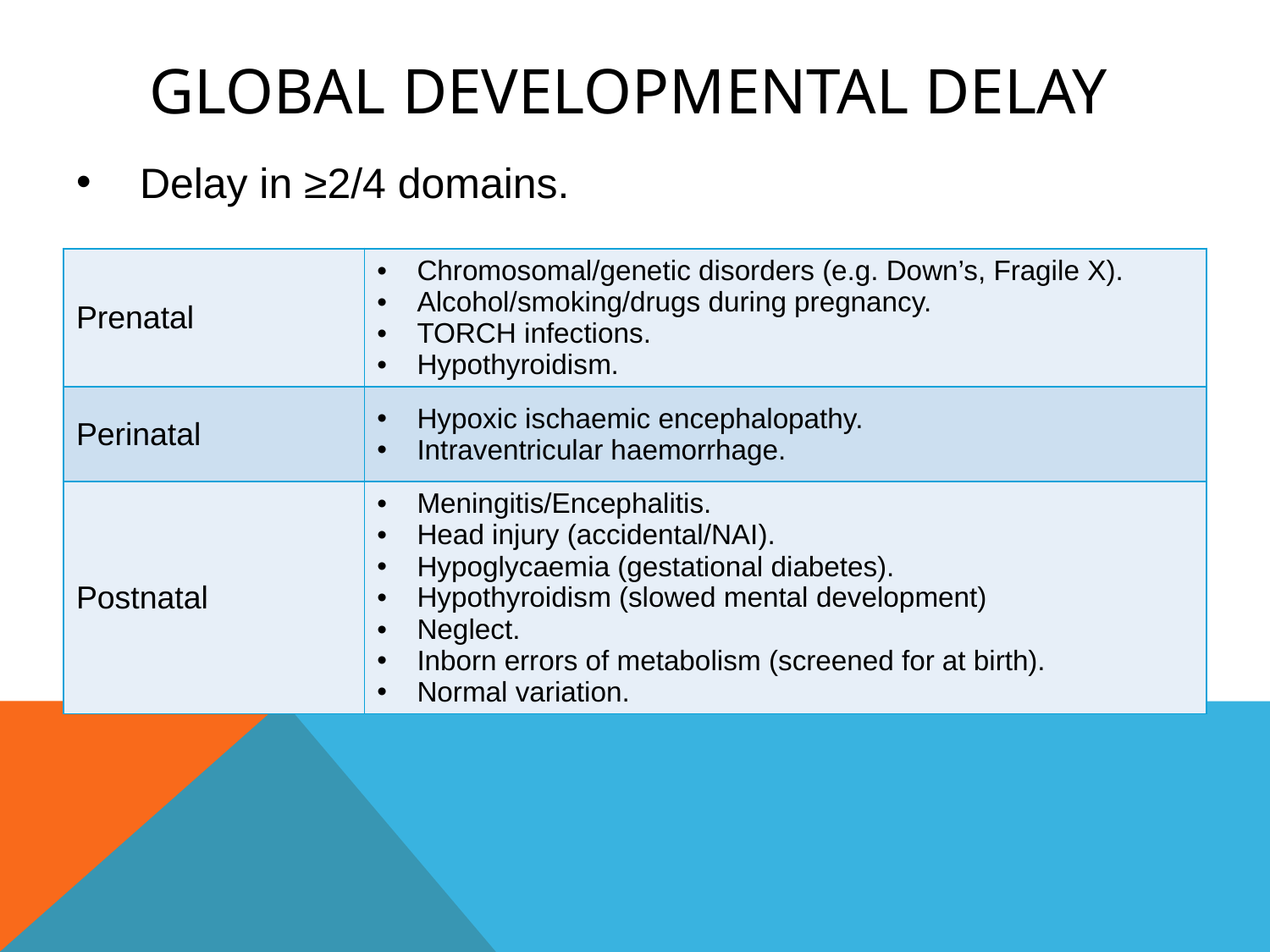

# Global Developmental delay
Delay in ≥2/4 domains.
| Prenatal | Chromosomal/genetic disorders (e.g. Down’s, Fragile X). Alcohol/smoking/drugs during pregnancy. TORCH infections. Hypothyroidism. |
| --- | --- |
| Perinatal | Hypoxic ischaemic encephalopathy. Intraventricular haemorrhage. |
| Postnatal | Meningitis/Encephalitis. Head injury (accidental/NAI). Hypoglycaemia (gestational diabetes). Hypothyroidism (slowed mental development) Neglect. Inborn errors of metabolism (screened for at birth). Normal variation. |

## Slide 15
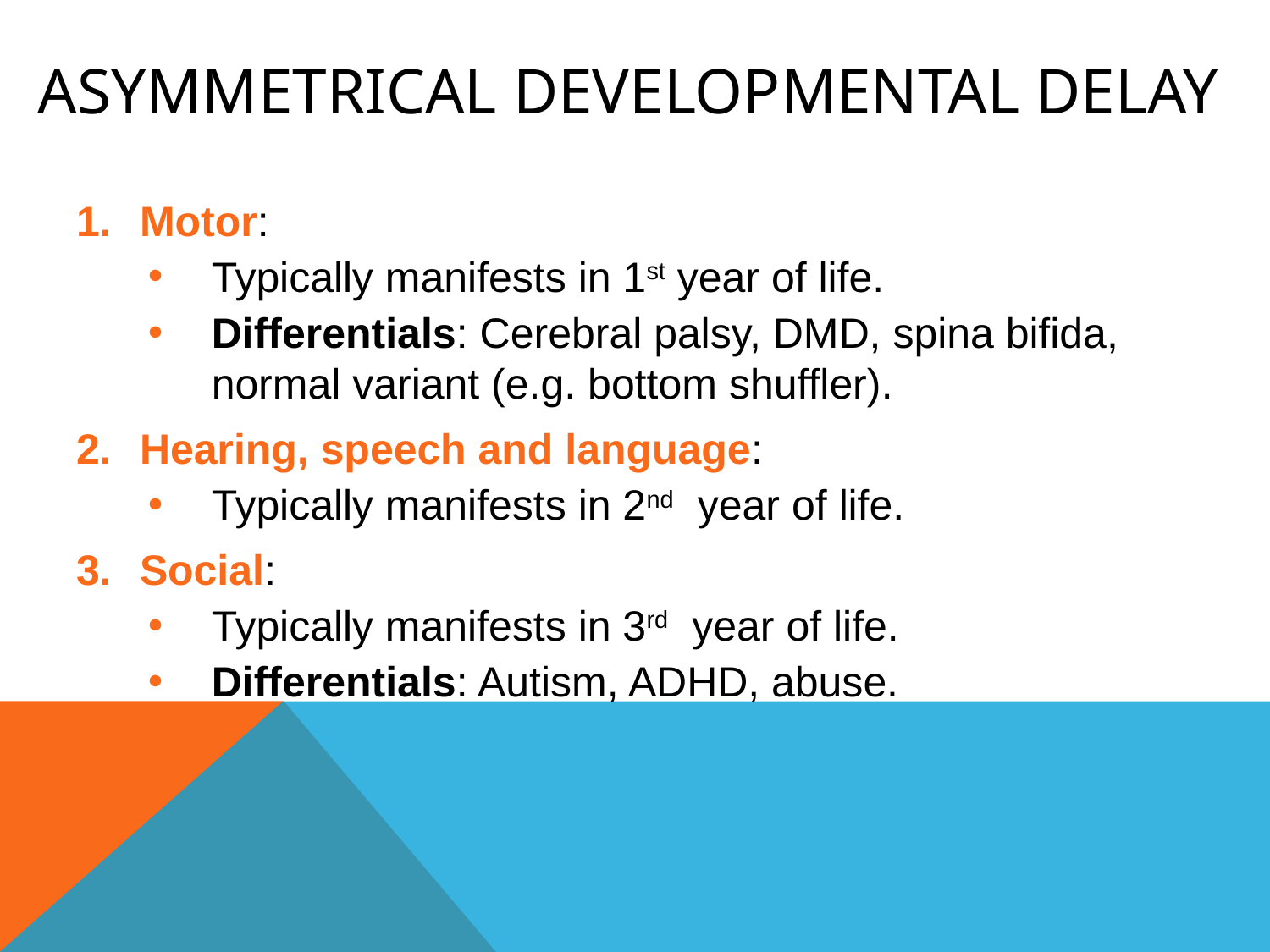

# Asymmetrical developmental delay
Motor:
Typically manifests in 1st year of life.
Differentials: Cerebral palsy, DMD, spina bifida, normal variant (e.g. bottom shuffler).
Hearing, speech and language:
Typically manifests in 2nd year of life.
Social:
Typically manifests in 3rd year of life.
Differentials: Autism, ADHD, abuse.

## Slide 16
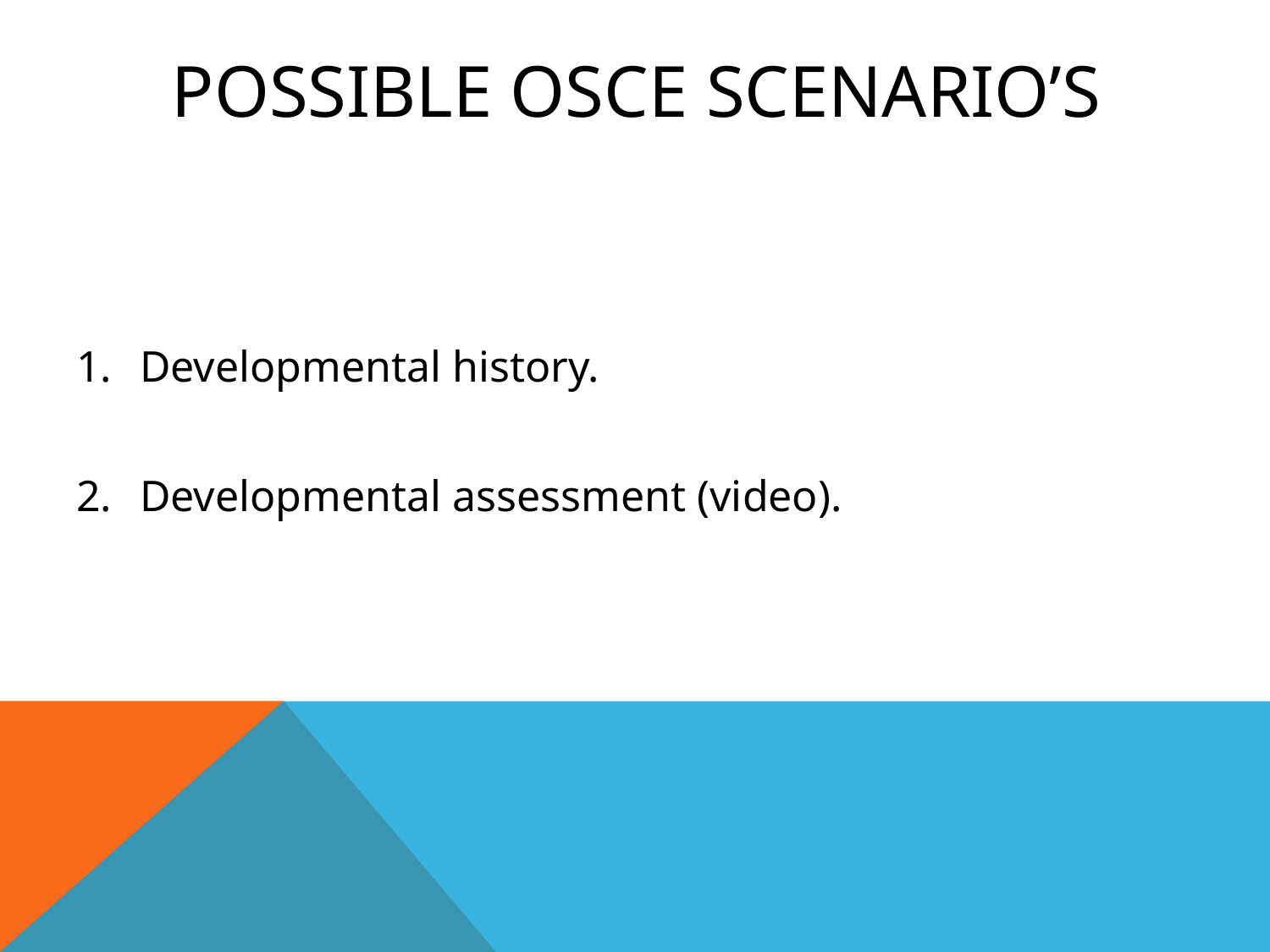

# Possible OSCE SCENARIO’S
Developmental history.
Developmental assessment (video).

## Slide 17
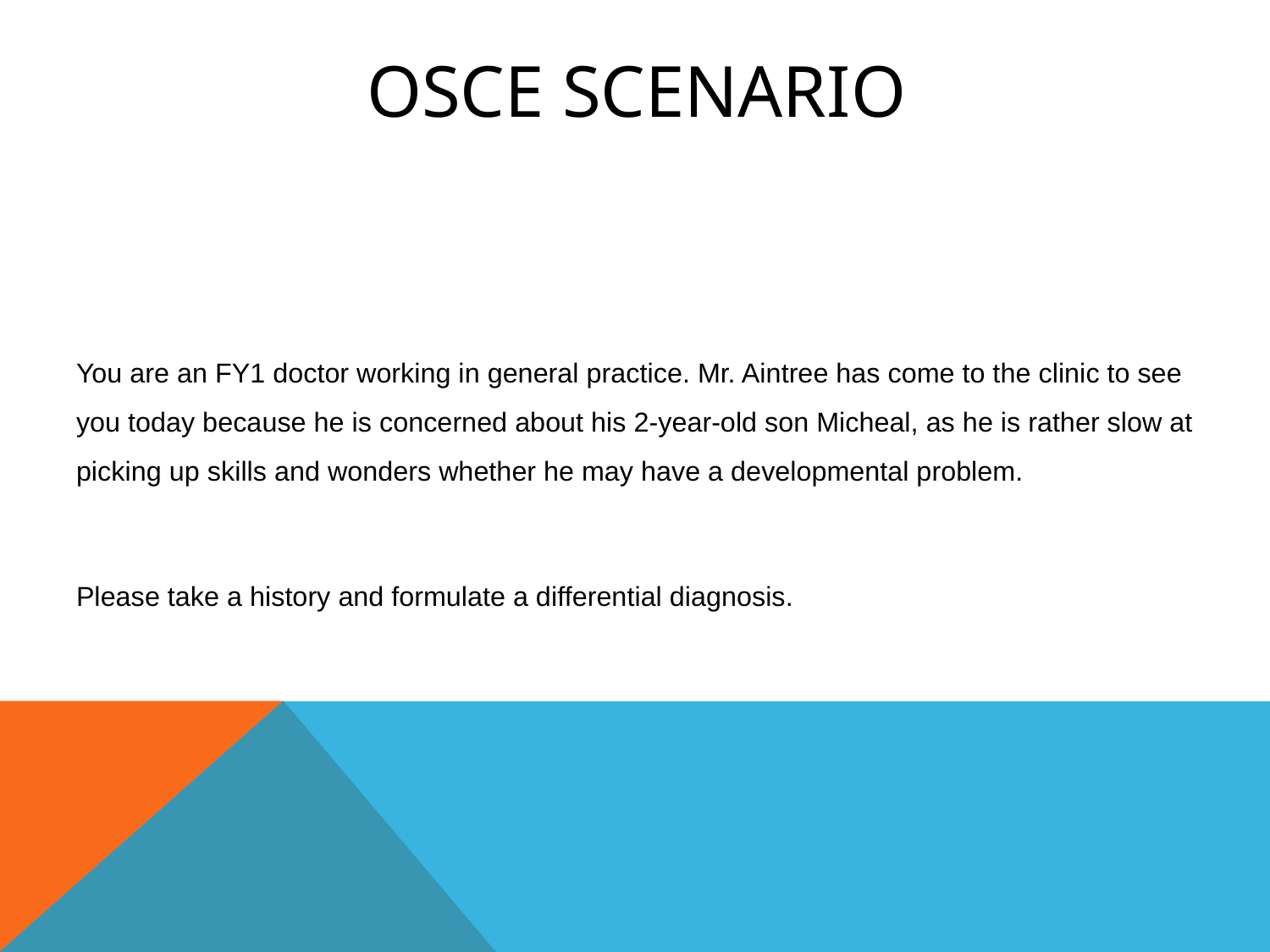

# OSCE SCENARIO
You are an FY1 doctor working in general practice. Mr. Aintree has come to the clinic to see you today because he is concerned about his 2-year-old son Micheal, as he is rather slow at picking up skills and wonders whether he may have a developmental problem.
Please take a history and formulate a differential diagnosis.

## Slide 18
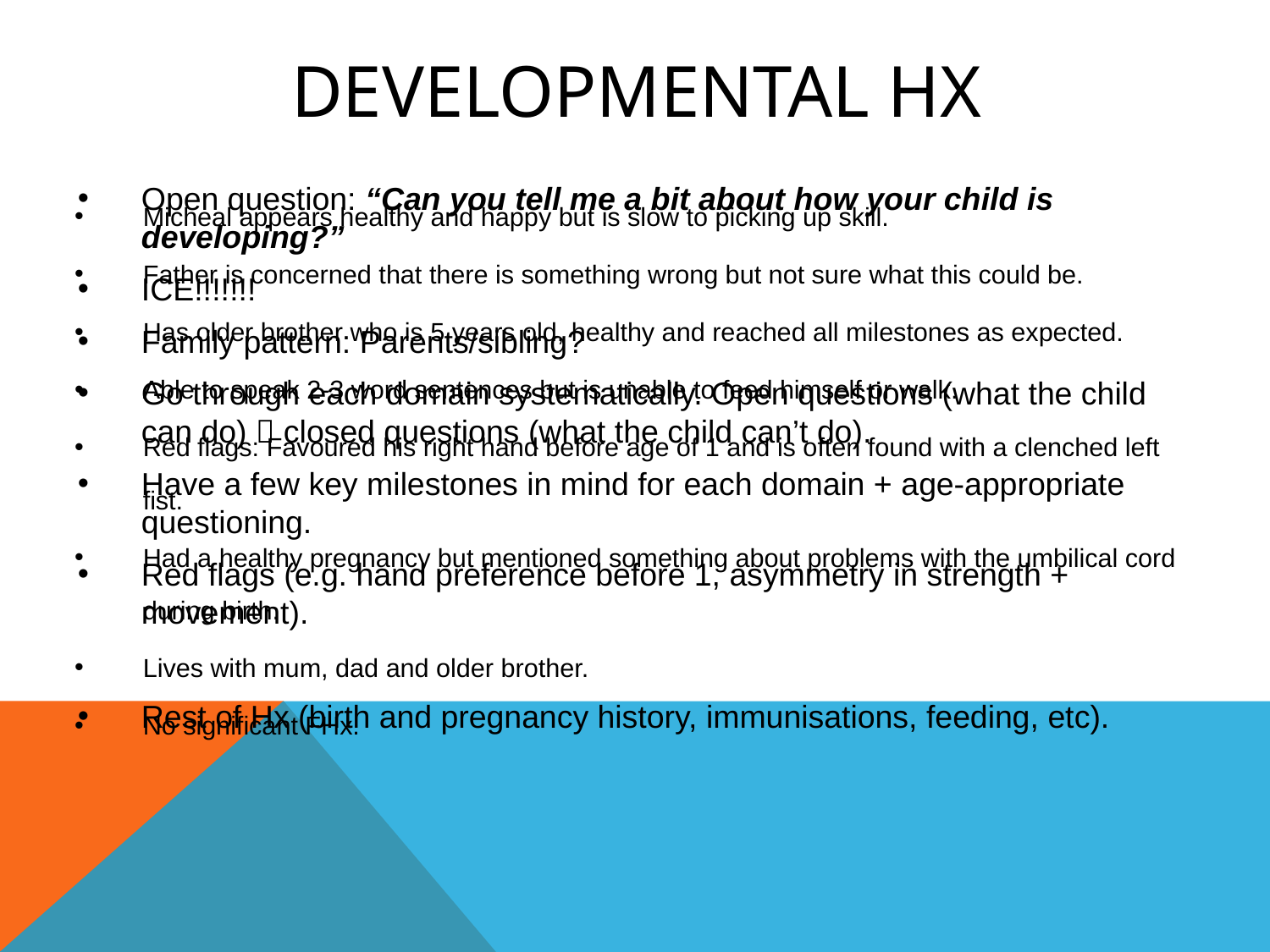

# Developmental Hx
Micheal appears healthy and happy but is slow to picking up skill.
Father is concerned that there is something wrong but not sure what this could be.
Has older brother who is 5 years old, healthy and reached all milestones as expected.
Able to speak 2-3 word sentences but is unable to feed himself or walk.
Red flags: Favoured his right hand before age of 1 and is often found with a clenched left fist.
Had a healthy pregnancy but mentioned something about problems with the umbilical cord during birth.
Lives with mum, dad and older brother.
No significant FHx.
Open question: “Can you tell me a bit about how your child is developing?”
ICE!!!!!!!
Family pattern: Parents/sibling?
Go through each domain systematically. Open questions (what the child can do)  closed questions (what the child can’t do).
Have a few key milestones in mind for each domain + age-appropriate questioning.
Red flags (e.g. hand preference before 1, asymmetry in strength + movement).
Rest of Hx (birth and pregnancy history, immunisations, feeding, etc).

## Slide 19
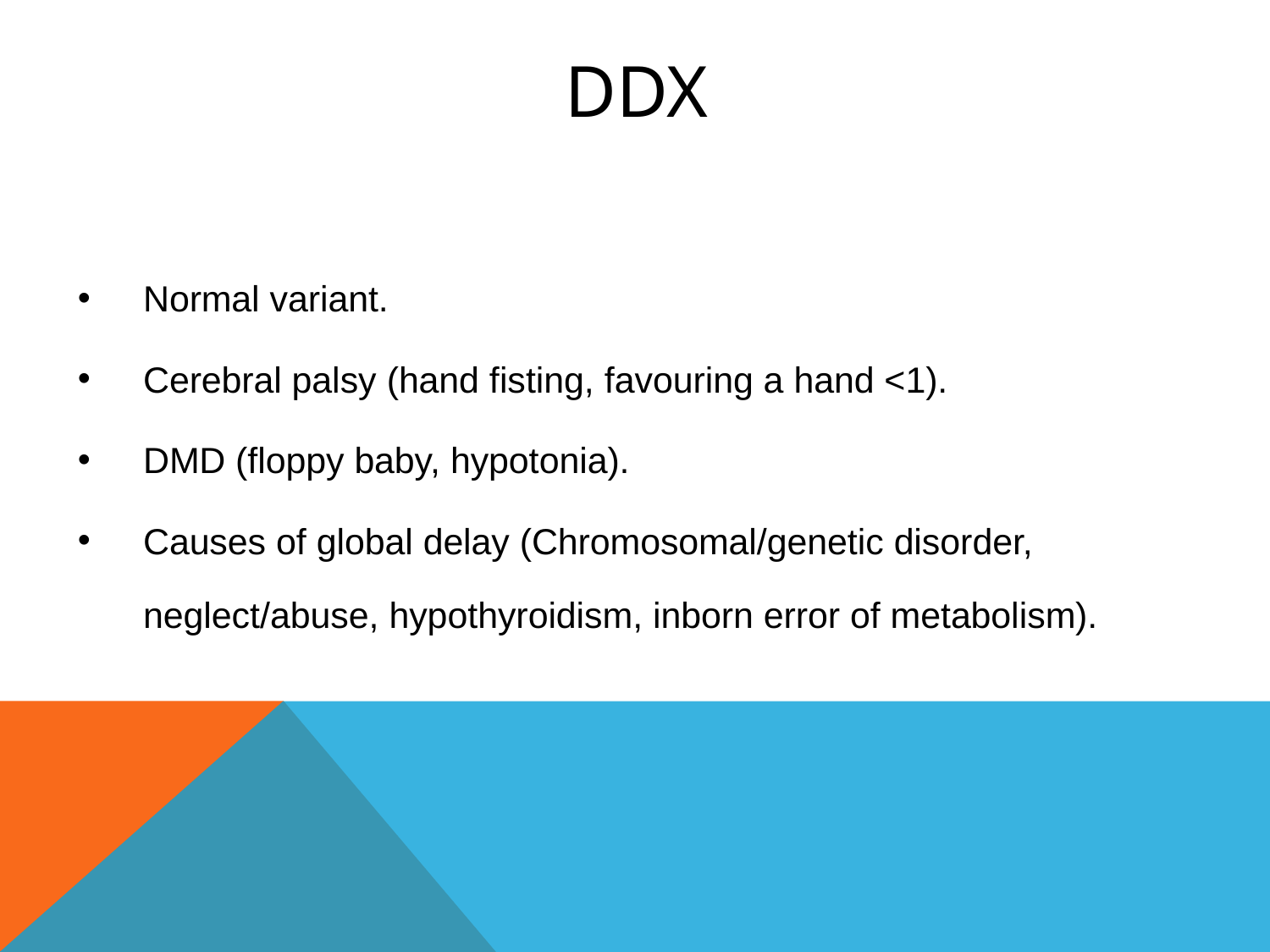

# DDX
Normal variant.
Cerebral palsy (hand fisting, favouring a hand <1).
DMD (floppy baby, hypotonia).
Causes of global delay (Chromosomal/genetic disorder, neglect/abuse, hypothyroidism, inborn error of metabolism).

## Slide 20
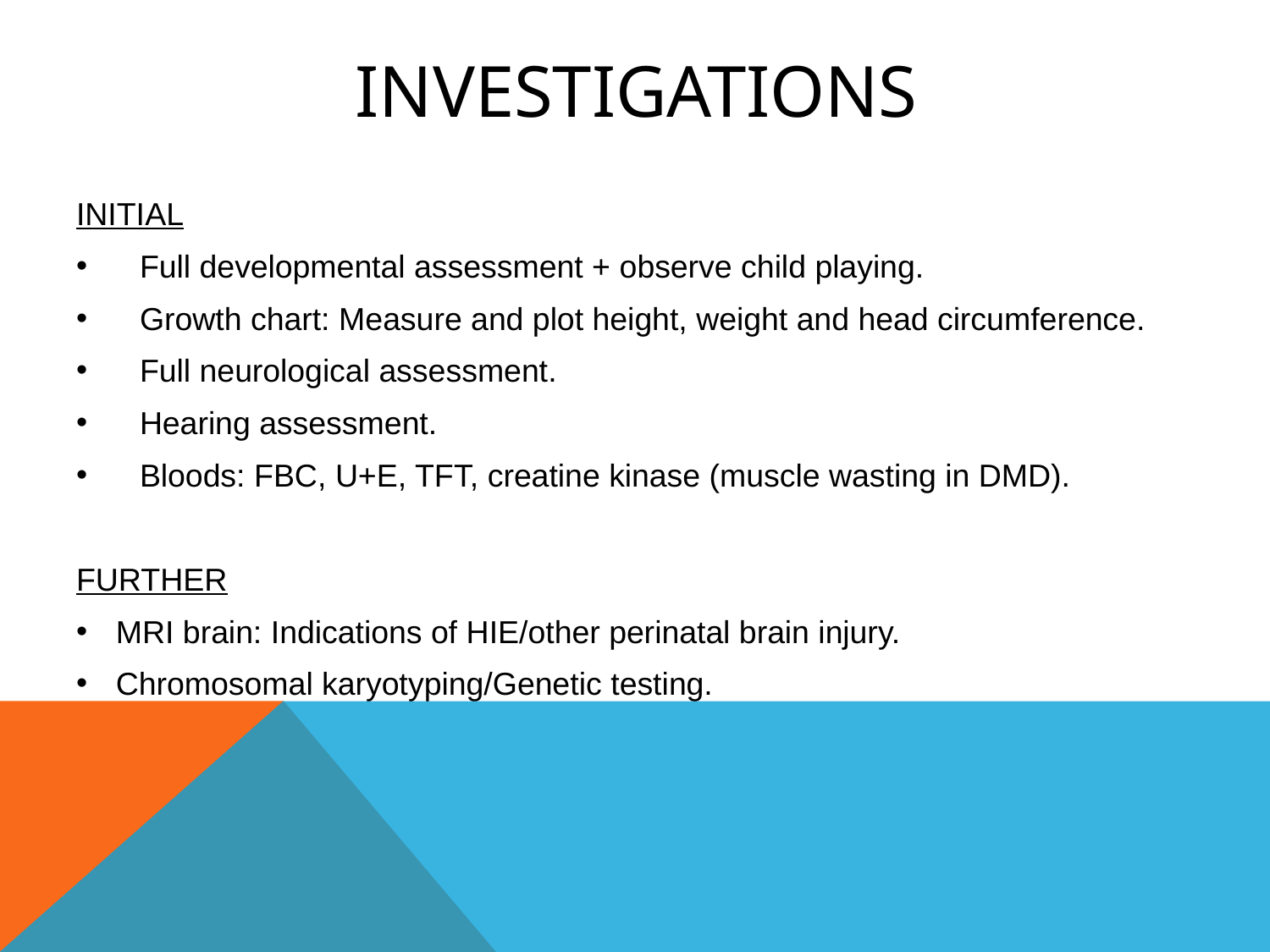

# INVESTIGATIONS
INITIAL
Full developmental assessment + observe child playing.
Growth chart: Measure and plot height, weight and head circumference.
Full neurological assessment.
Hearing assessment.
Bloods: FBC, U+E, TFT, creatine kinase (muscle wasting in DMD).
FURTHER
MRI brain: Indications of HIE/other perinatal brain injury.
Chromosomal karyotyping/Genetic testing.

## Slide 21
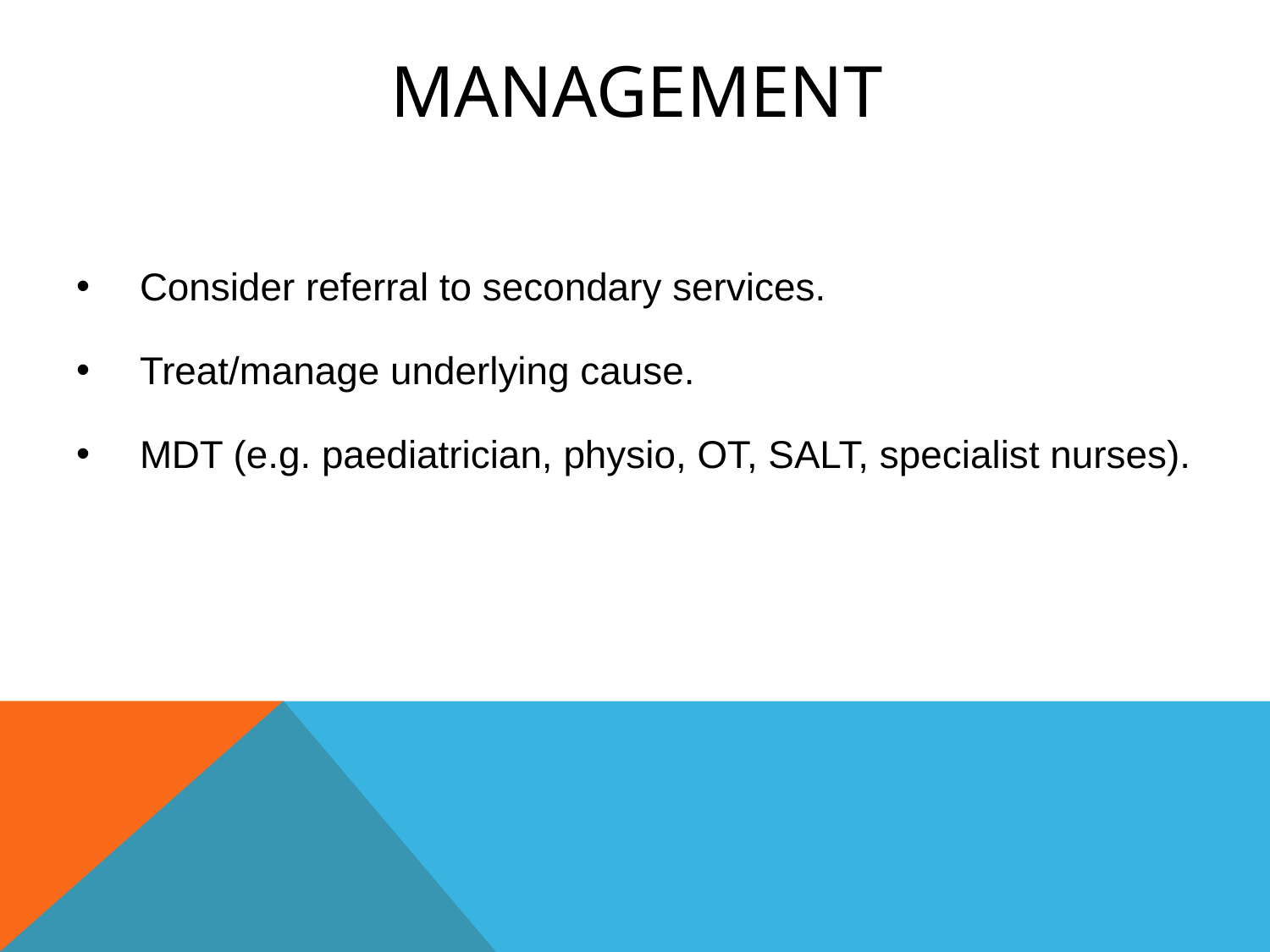

# MANAGEMENT
Consider referral to secondary services.
Treat/manage underlying cause.
MDT (e.g. paediatrician, physio, OT, SALT, specialist nurses).

## Slide 22
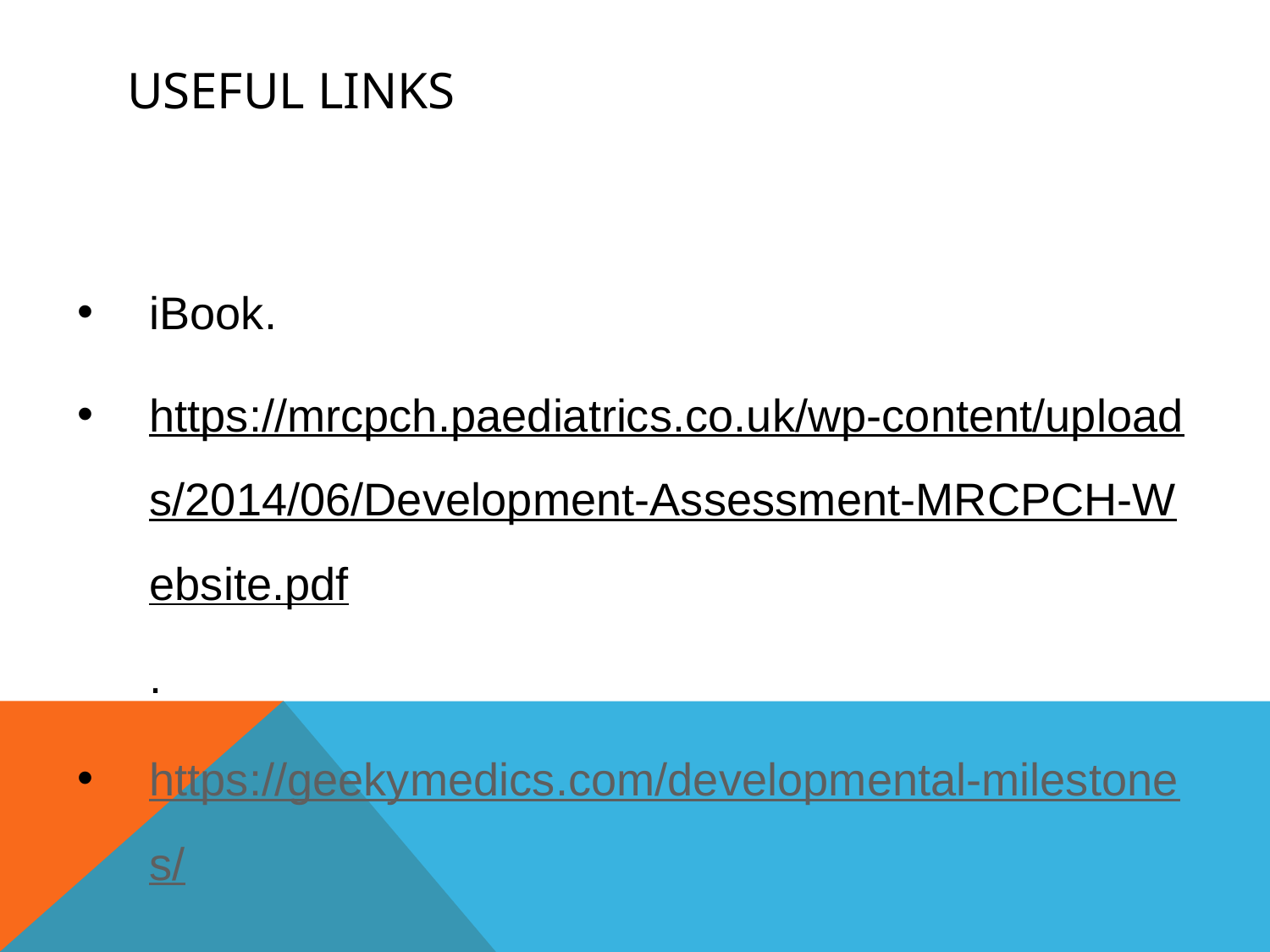

# Useful links
iBook.
https://mrcpch.paediatrics.co.uk/wp-content/uploads/2014/06/Development-Assessment-MRCPCH-Website.pdf.
https://geekymedics.com/developmental-milestones/

## Slide 23
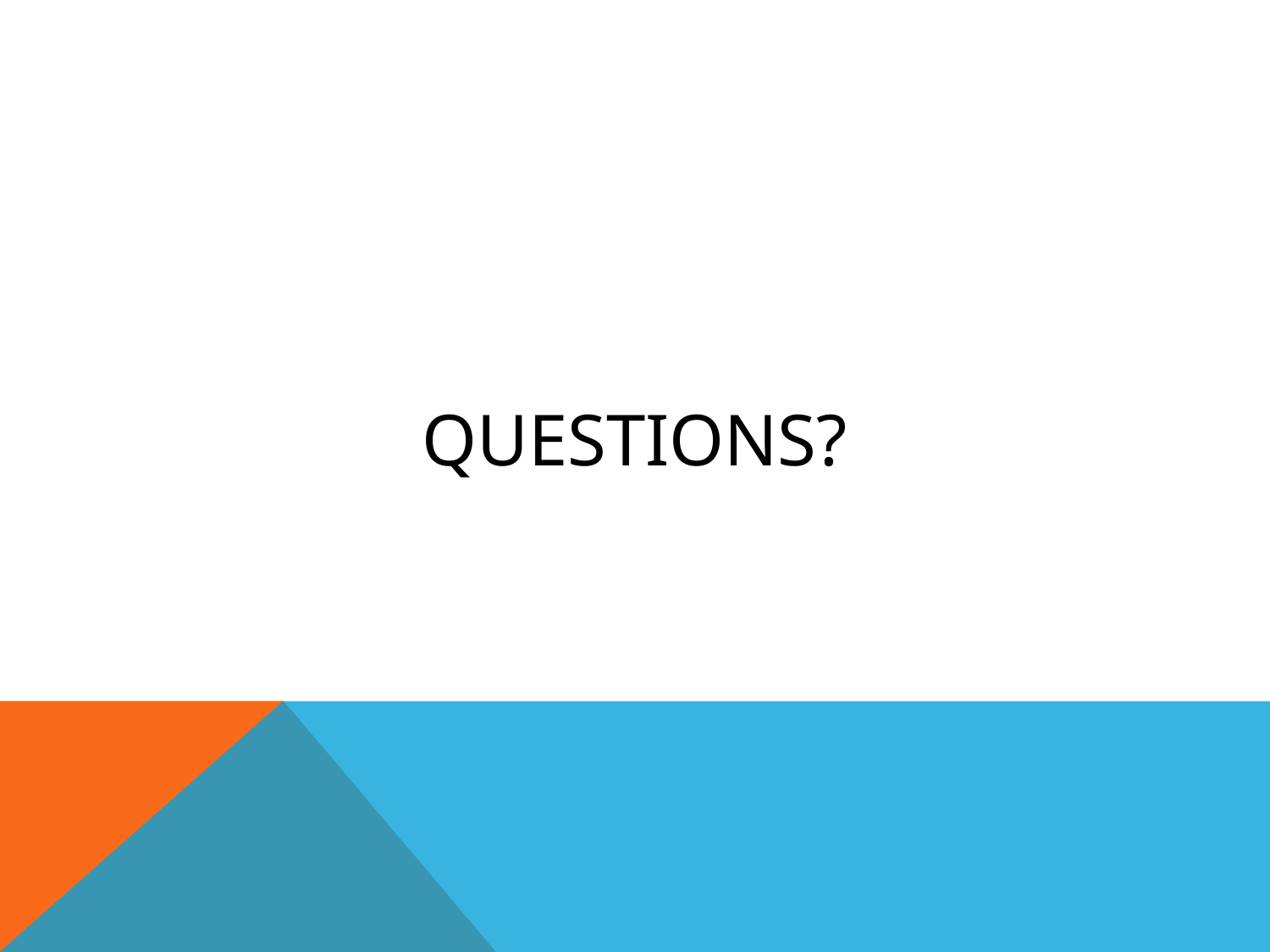

# Questions?

## Slide 24
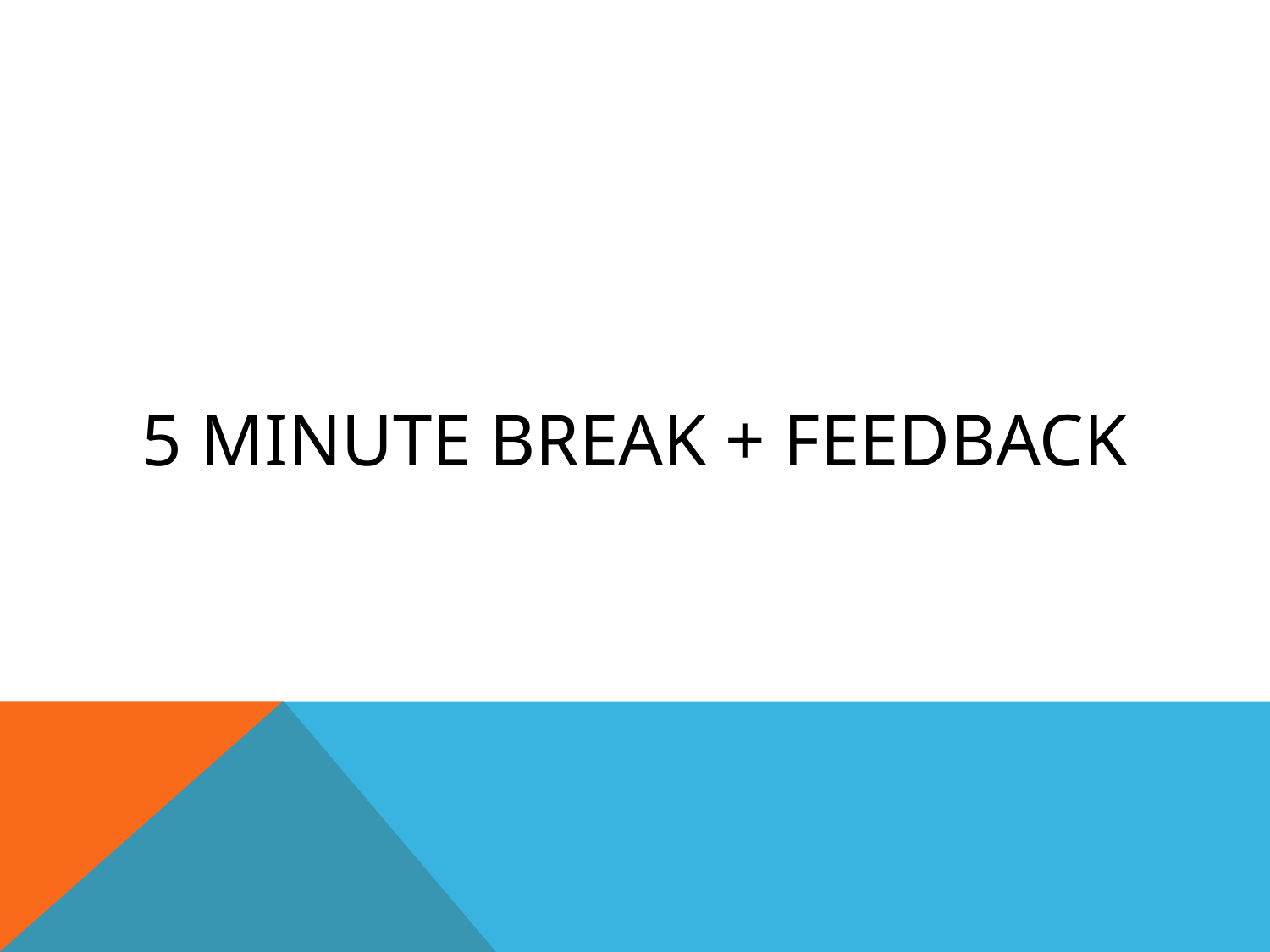

# 5 minute break + Feedback
