# Supplementary material for: Evaluation of Online Near-Peer Teaching for Penultimate-Year Objective Structured Clinical Examinations in the COVID-19 Era: Longitudinal Study
Source: JMIR Med Educ. 2022 May 26;8(2):e37872. doi: 10.2196/37872 (PMC9185334; doi:10.2196/37872)
Supplement: Multimedia Appendix 2 [file mededu_v8i2e37872_app2.pptx]

## Slide 1
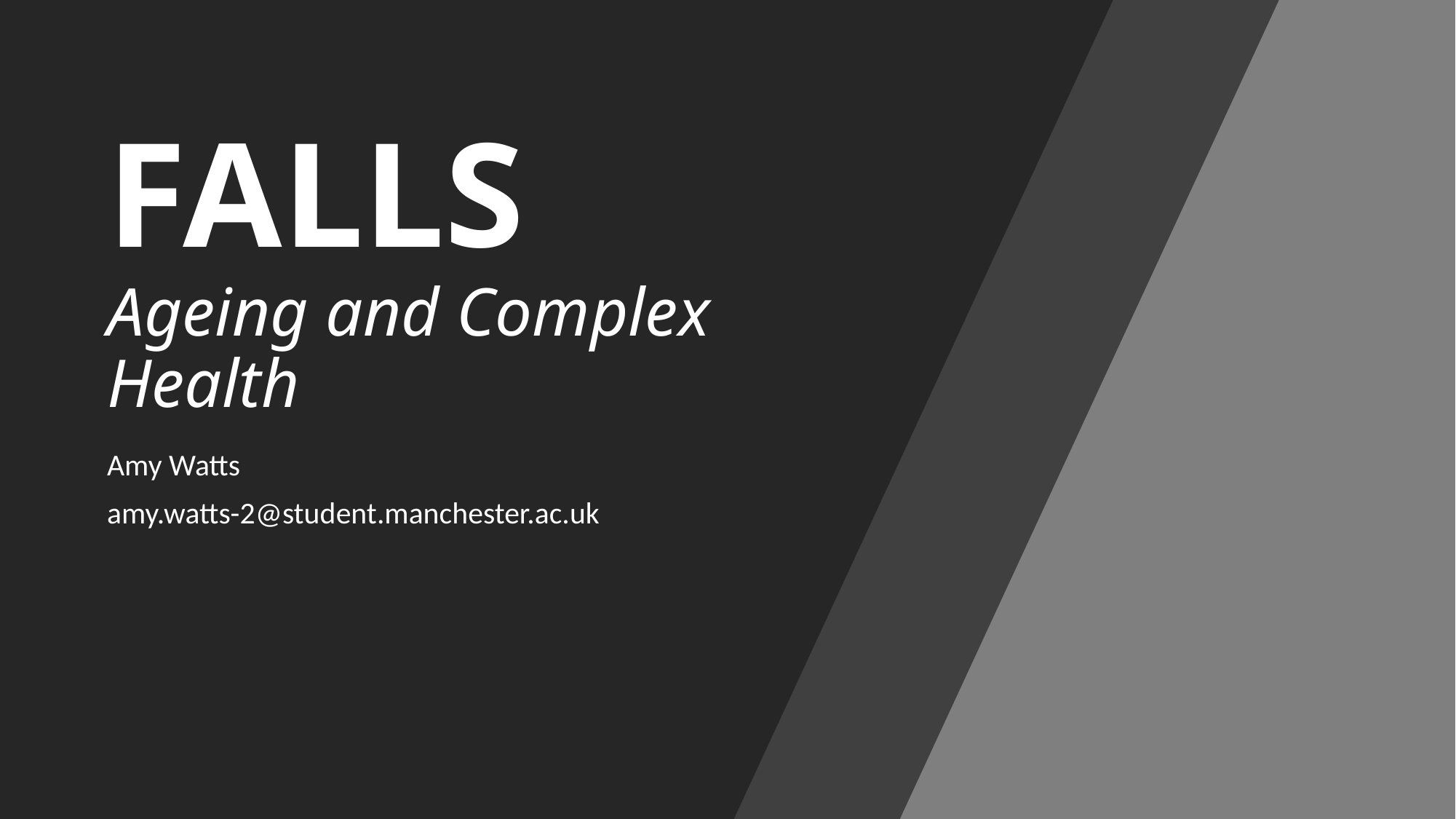

# FALLS Ageing and Complex Health
Amy Watts
amy.watts-2@student.manchester.ac.uk

## Slide 2
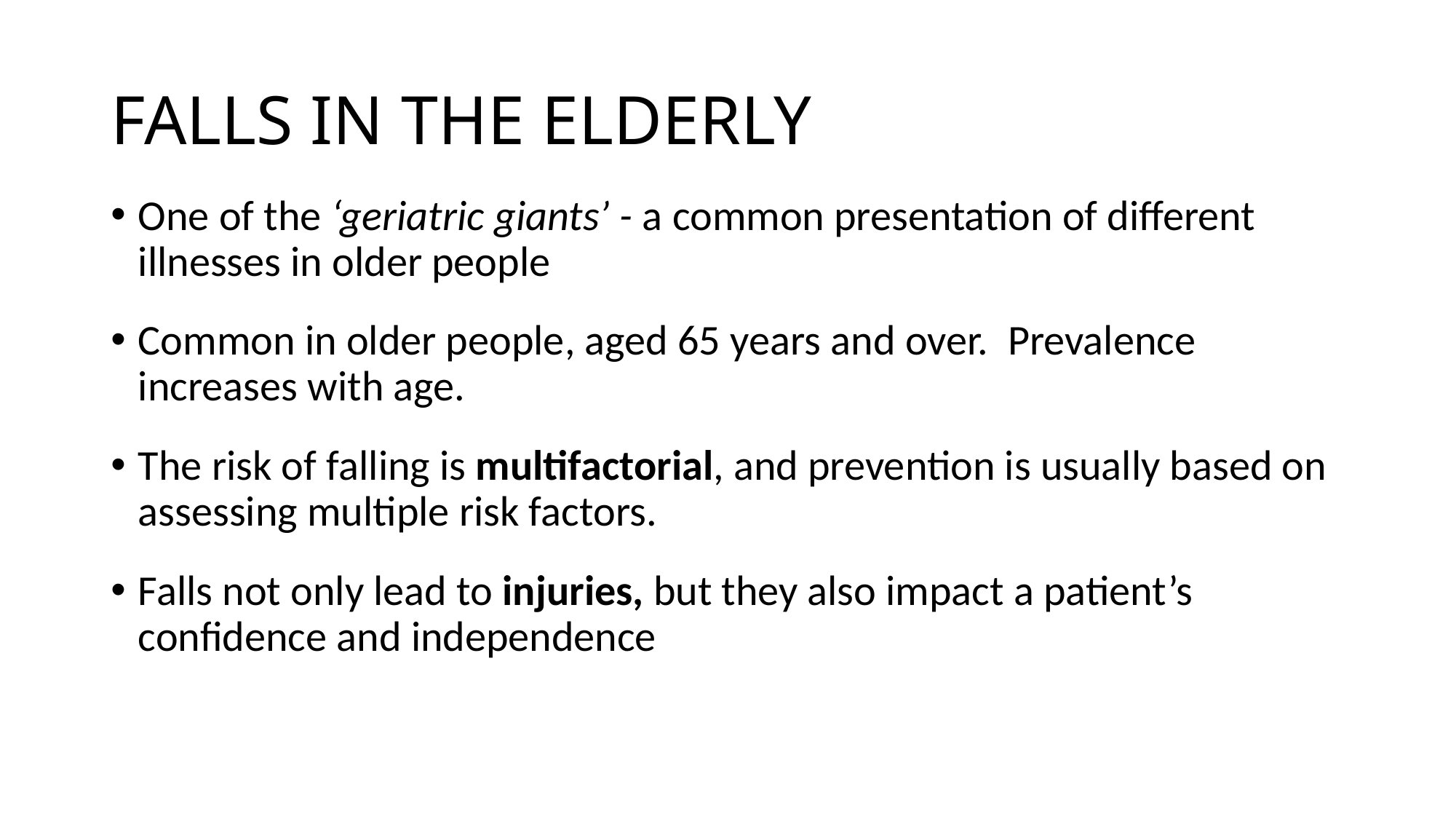

# FALLS IN THE ELDERLY
One of the ‘geriatric giants’ - a common presentation of different illnesses in older people
Common in older people, aged 65 years and over. Prevalence increases with age.
The risk of falling is multifactorial, and prevention is usually based on assessing multiple risk factors.
Falls not only lead to injuries, but they also impact a patient’s confidence and independence

## Slide 3
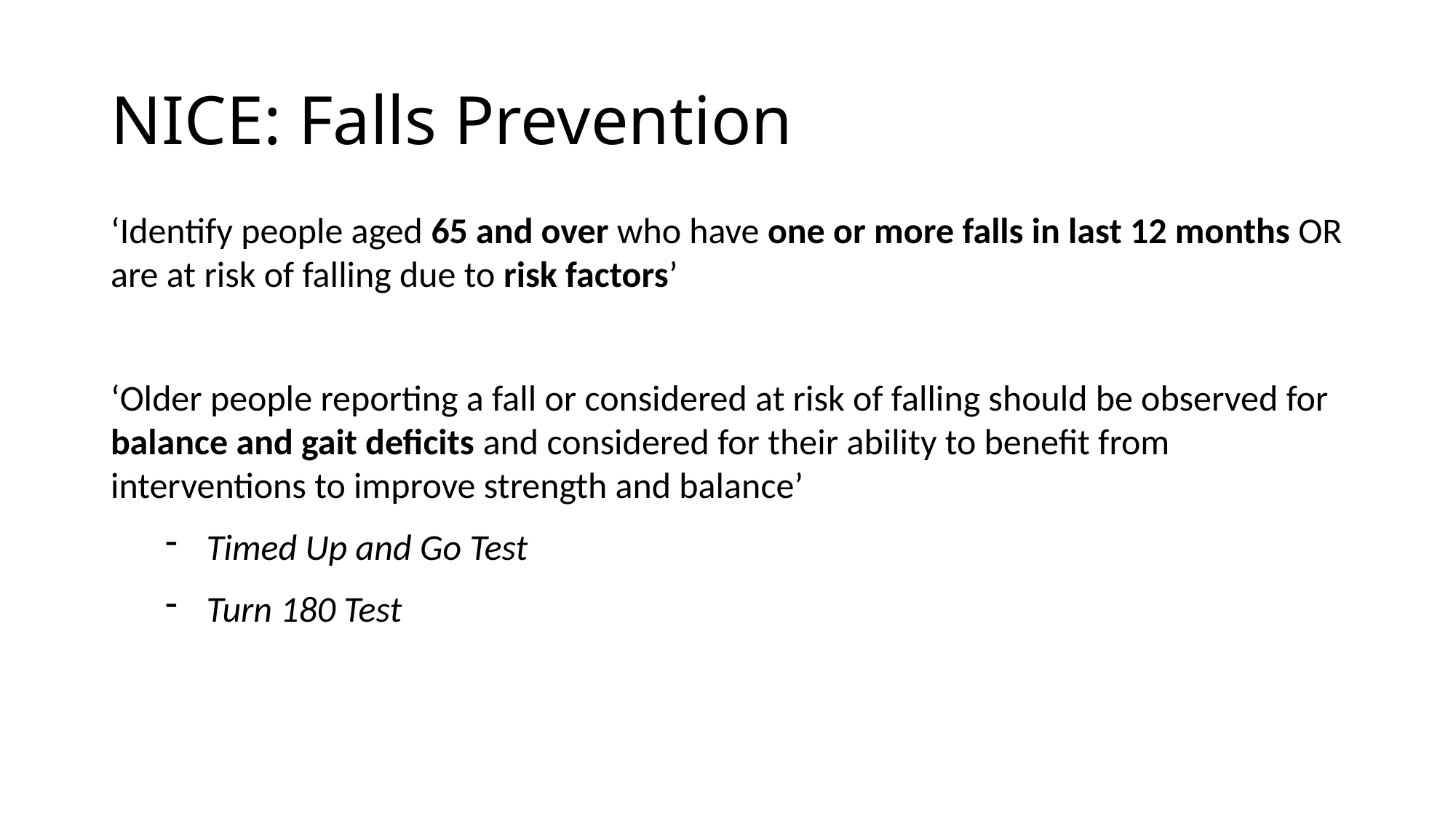

# NICE: Falls Prevention
‘Identify people aged 65 and over who have one or more falls in last 12 months OR are at risk of falling due to risk factors’
‘Older people reporting a fall or considered at risk of falling should be observed for balance and gait deficits and considered for their ability to benefit from interventions to improve strength and balance’
Timed Up and Go Test
Turn 180 Test

## Slide 4
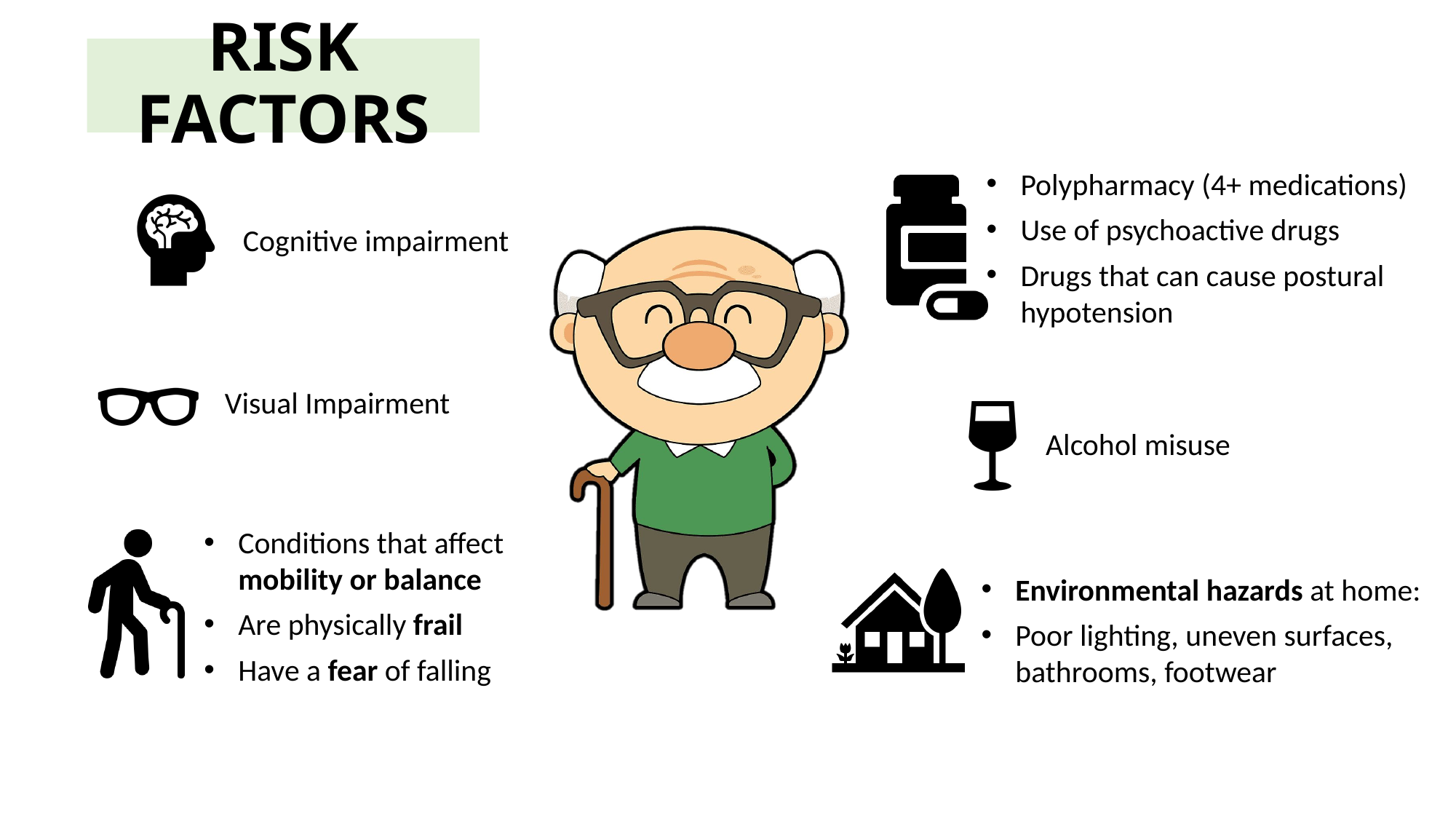

# RISK FACTORS
Polypharmacy (4+ medications)
Use of psychoactive drugs
Drugs that can cause postural hypotension
Cognitive impairment
Visual Impairment
Alcohol misuse
Conditions that affect mobility or balance
Are physically frail
Have a fear of falling
Environmental hazards at home:
Poor lighting, uneven surfaces, bathrooms, footwear

## Slide 5
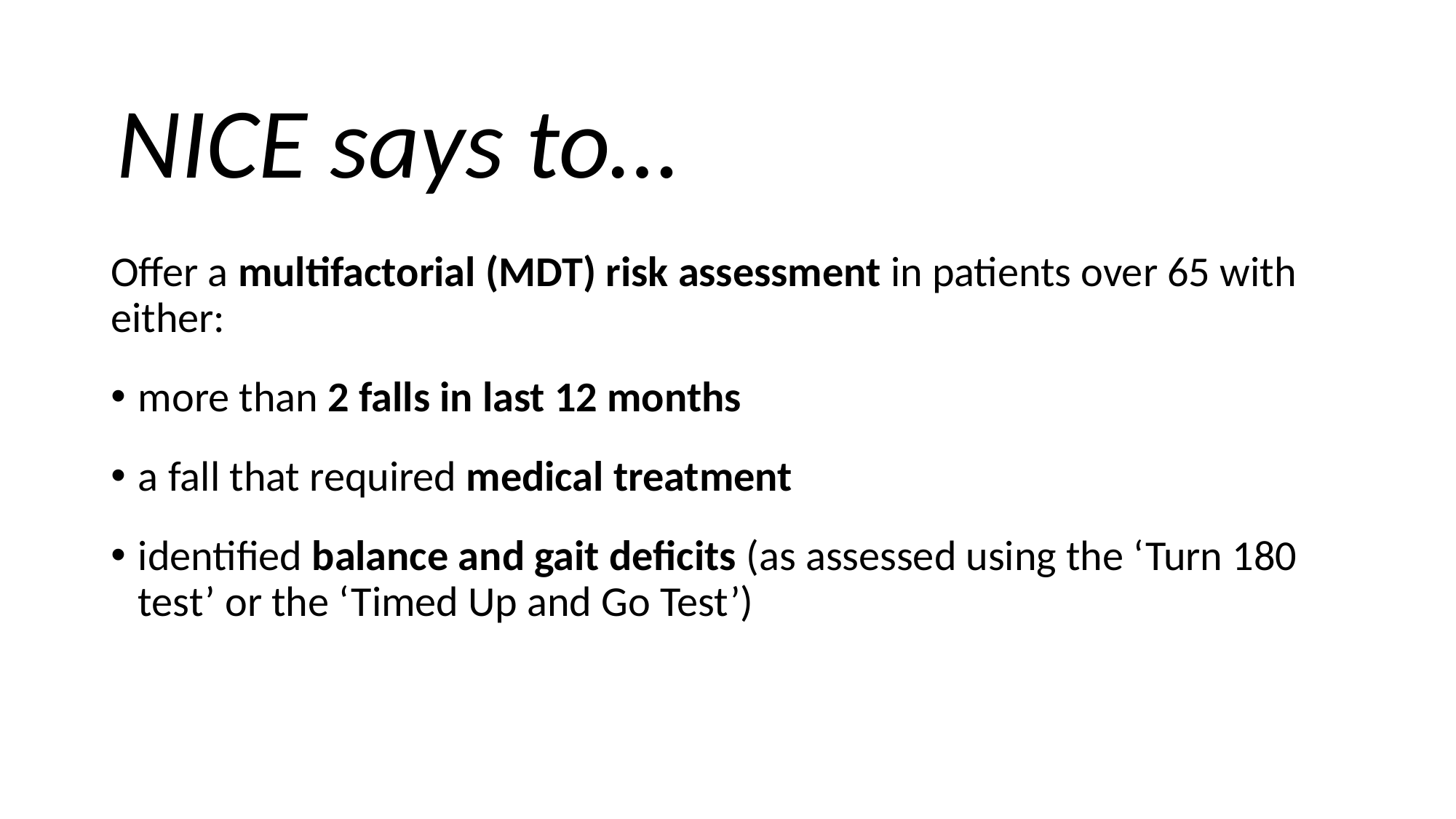

NICE says to…
Offer a multifactorial (MDT) risk assessment in patients over 65 with either:
more than 2 falls in last 12 months
a fall that required medical treatment
identified balance and gait deficits (as assessed using the ‘Turn 180 test’ or the ‘Timed Up and Go Test’)

## Slide 6
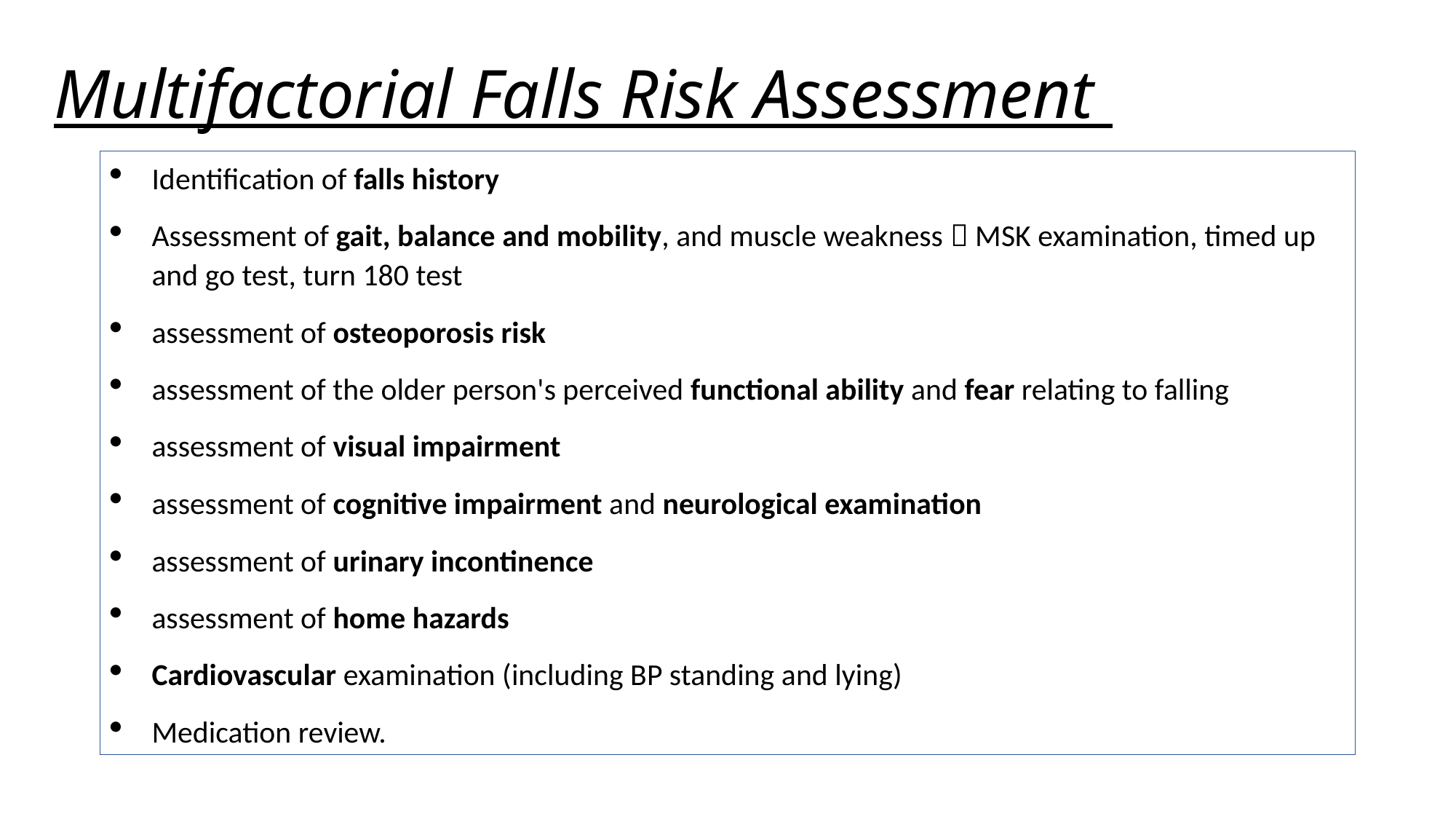

# Multifactorial Falls Risk Assessment
Identification of falls history
Assessment of gait, balance and mobility, and muscle weakness  MSK examination, timed up and go test, turn 180 test
assessment of osteoporosis risk
assessment of the older person's perceived functional ability and fear relating to falling
assessment of visual impairment
assessment of cognitive impairment and neurological examination
assessment of urinary incontinence
assessment of home hazards
Cardiovascular examination (including BP standing and lying)
Medication review.

## Slide 7
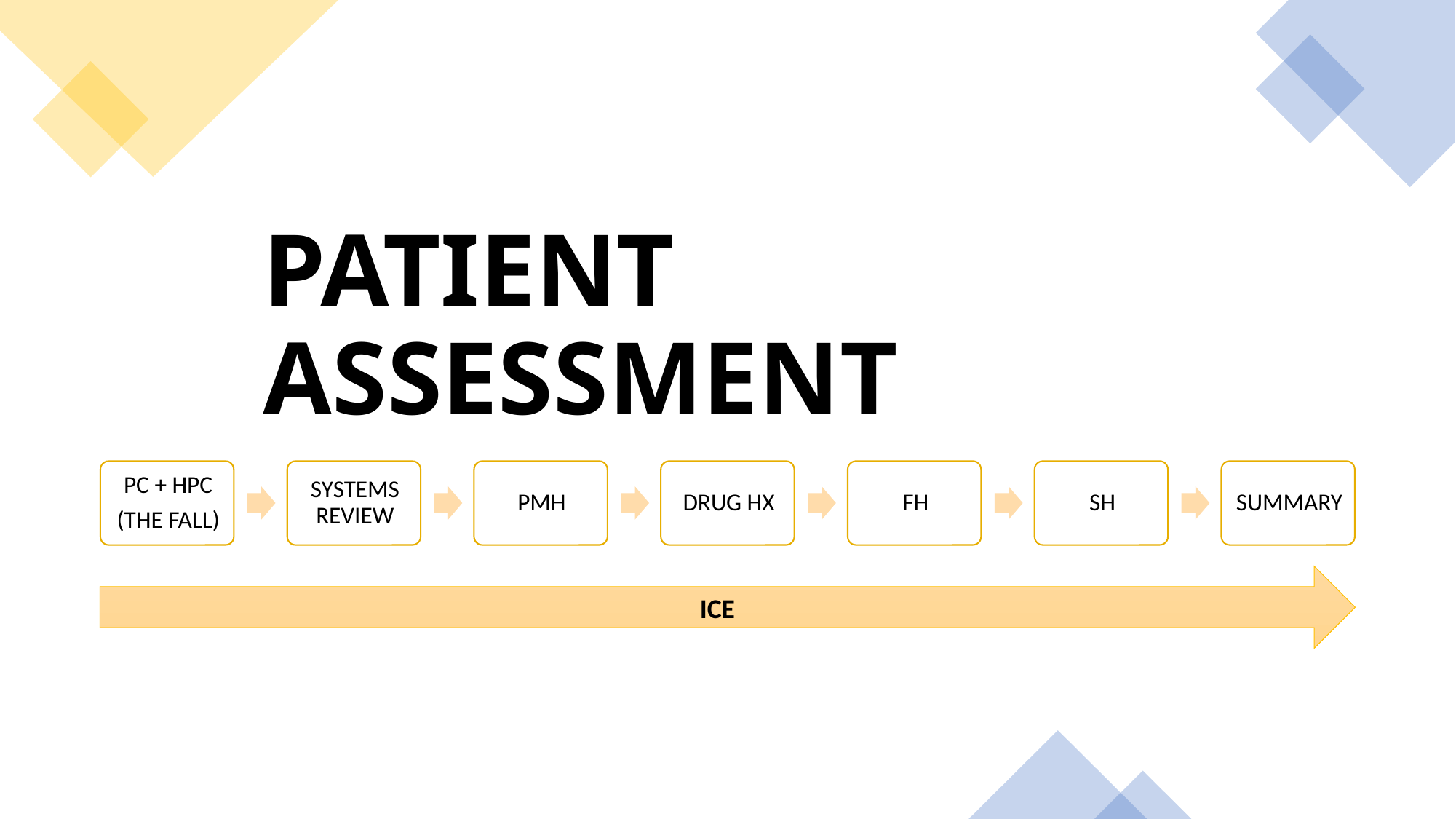

# PATIENT ASSESSMENT
ICE

## Slide 8
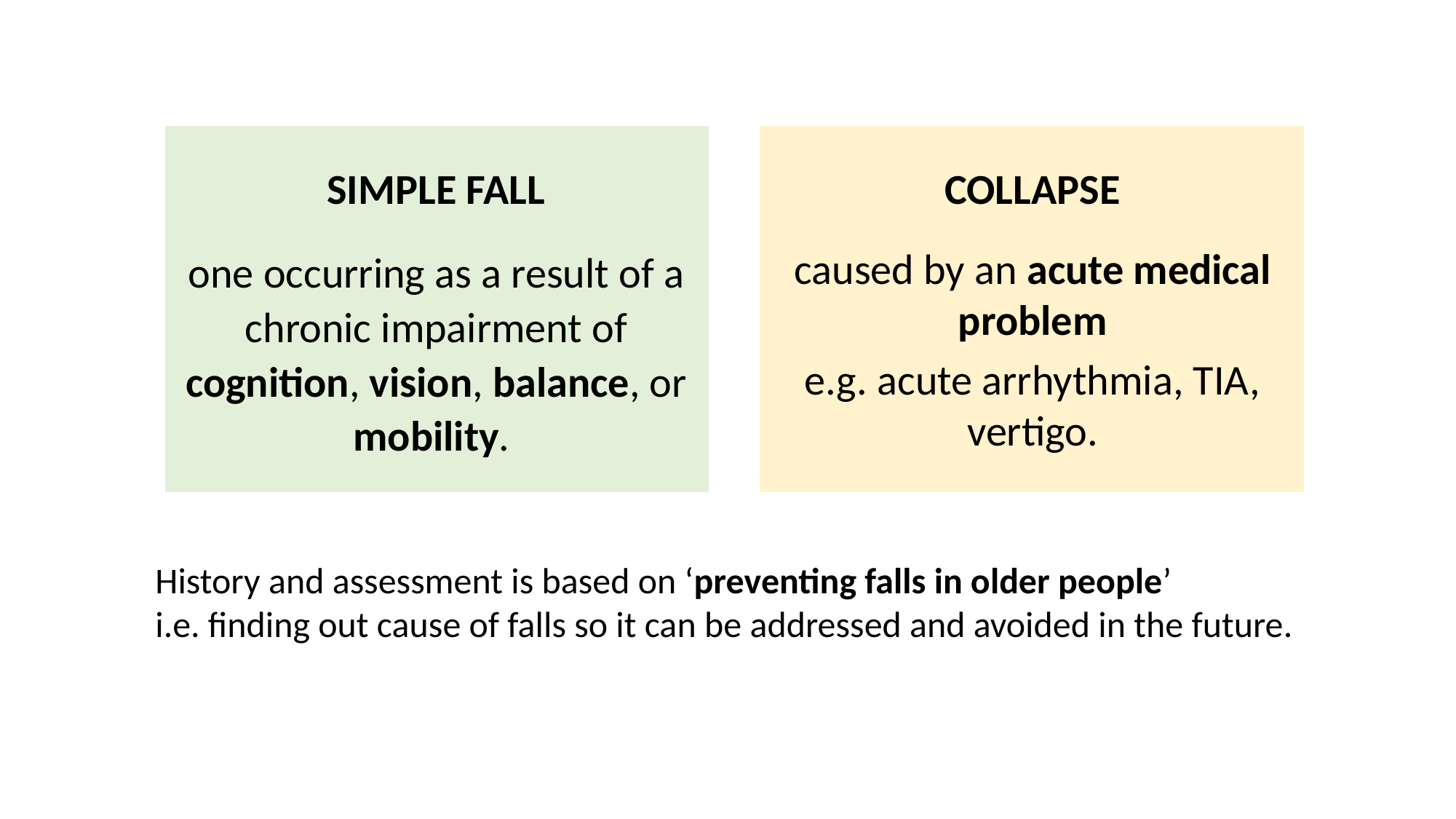

SIMPLE FALL
COLLAPSE
one occurring as a result of a chronic impairment of cognition, vision, balance, or mobility.
caused by an acute medical problem
e.g. acute arrhythmia, TIA, vertigo.
History and assessment is based on ‘preventing falls in older people’
i.e. finding out cause of falls so it can be addressed and avoided in the future.

## Slide 9
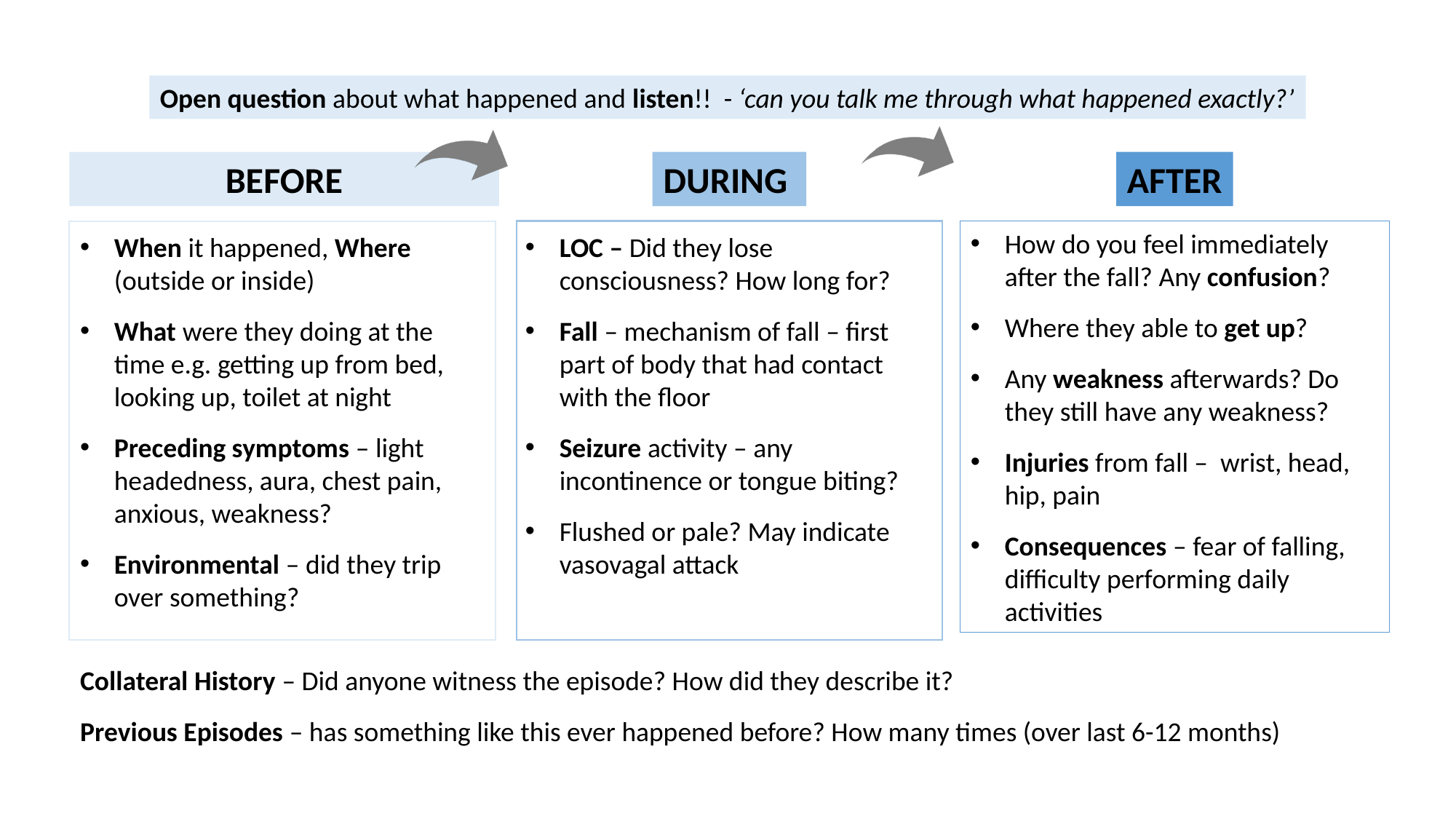

Open question about what happened and listen!! - ‘can you talk me through what happened exactly?’
BEFORE
DURING
AFTER
How do you feel immediately after the fall? Any confusion?
Where they able to get up?
Any weakness afterwards? Do they still have any weakness?
Injuries from fall – wrist, head, hip, pain
Consequences – fear of falling, difficulty performing daily activities
When it happened, Where (outside or inside)
What were they doing at the time e.g. getting up from bed, looking up, toilet at night
Preceding symptoms – light headedness, aura, chest pain, anxious, weakness?
Environmental – did they trip over something?
LOC – Did they lose consciousness? How long for?
Fall – mechanism of fall – first part of body that had contact with the floor
Seizure activity – any incontinence or tongue biting?
Flushed or pale? May indicate vasovagal attack
Collateral History – Did anyone witness the episode? How did they describe it?
Previous Episodes – has something like this ever happened before? How many times (over last 6-12 months)

## Slide 10
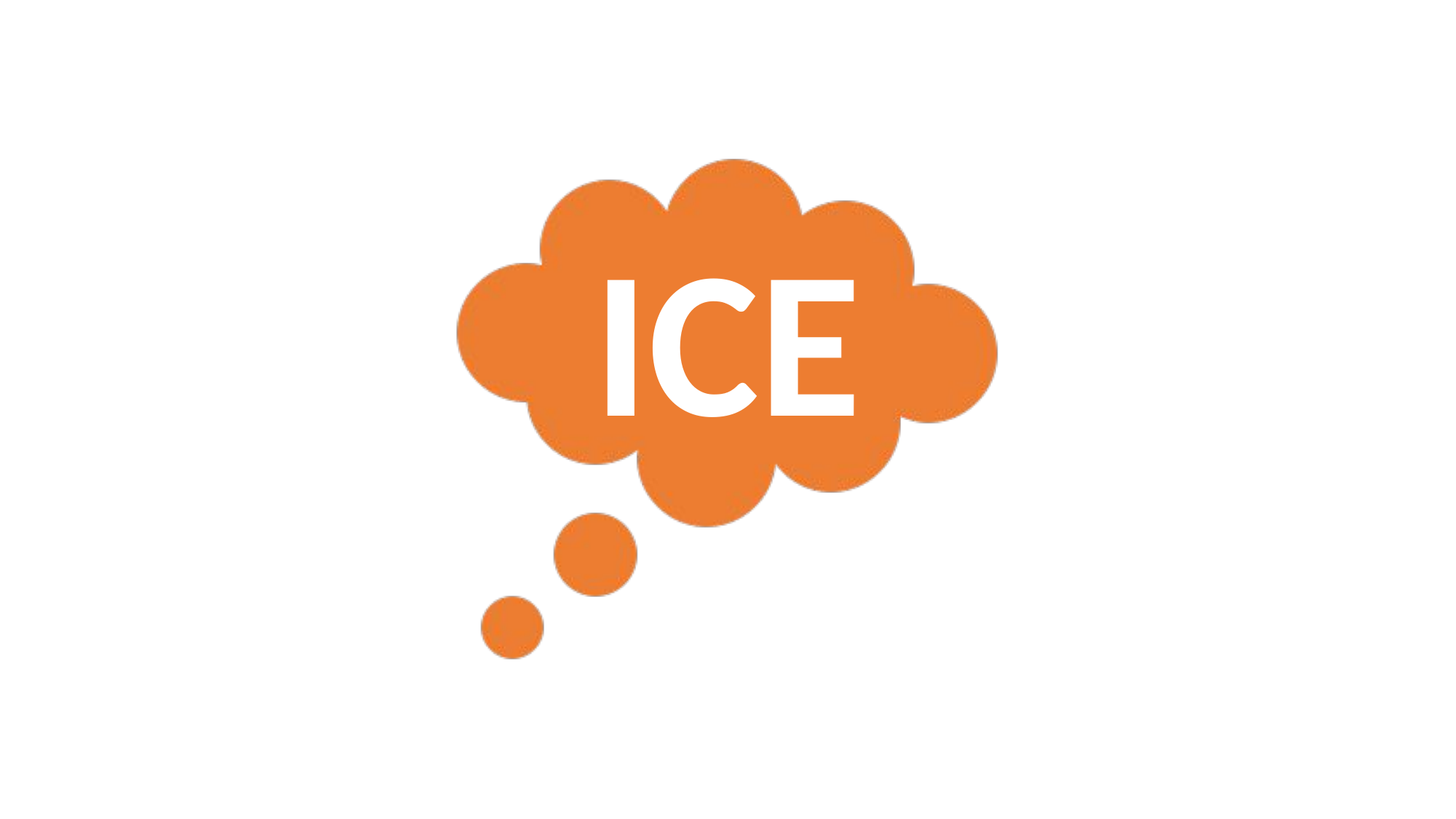

ICE

## Slide 11
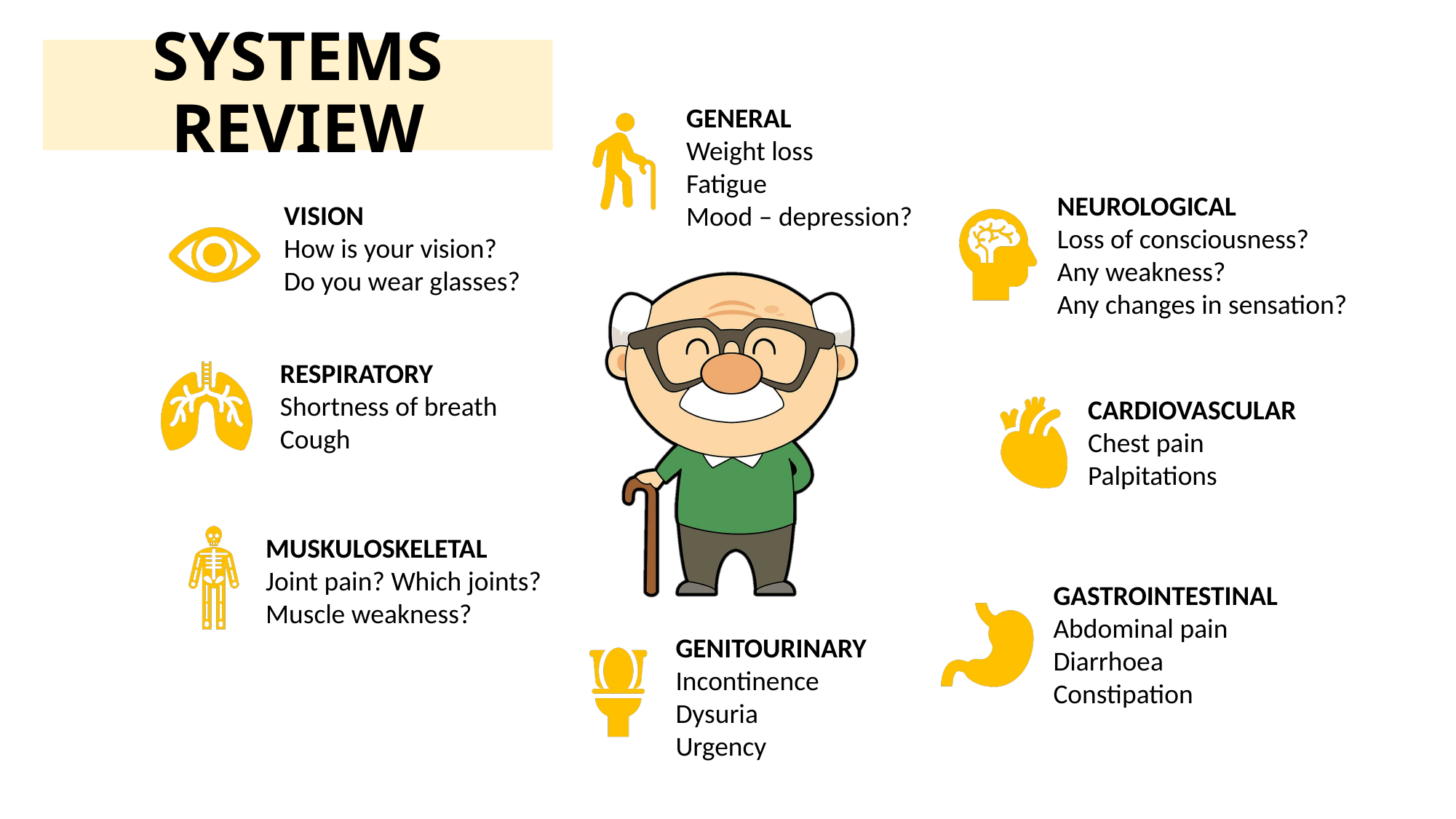

# SYSTEMS REVIEW
GENERAL
Weight loss
Fatigue
Mood – depression?
NEUROLOGICAL
Loss of consciousness?
Any weakness?
Any changes in sensation?
VISION
How is your vision?
Do you wear glasses?
RESPIRATORY
Shortness of breath
Cough
CARDIOVASCULAR
Chest pain
Palpitations
MUSKULOSKELETAL
Joint pain? Which joints?
Muscle weakness?
GASTROINTESTINAL
Abdominal pain
Diarrhoea
Constipation
GENITOURINARY
Incontinence
Dysuria
Urgency

## Slide 12
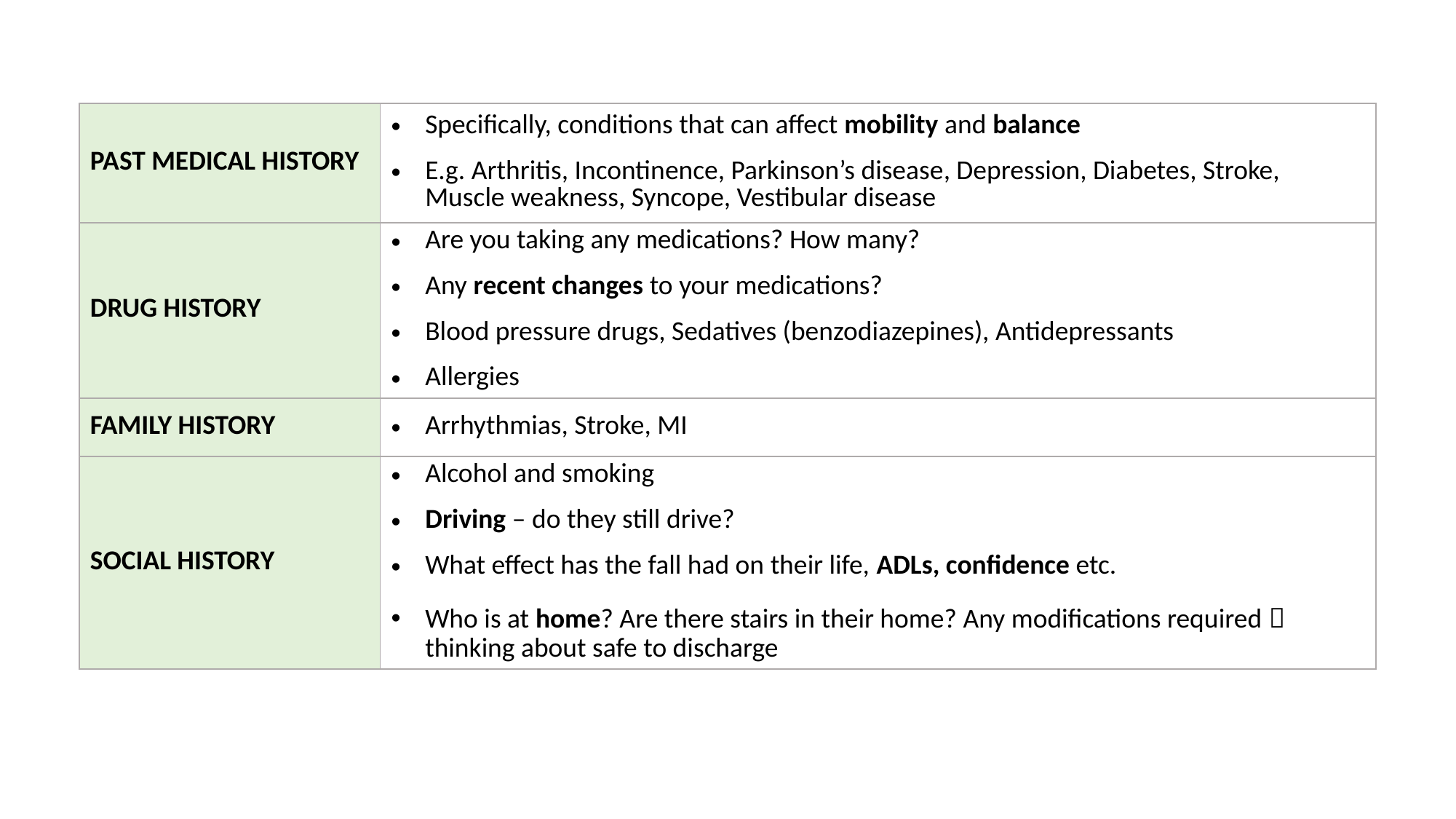

| PAST MEDICAL HISTORY | Specifically, conditions that can affect mobility and balance E.g. Arthritis, Incontinence, Parkinson’s disease, Depression, Diabetes, Stroke, Muscle weakness, Syncope, Vestibular disease |
| --- | --- |
| DRUG HISTORY | Are you taking any medications? How many? Any recent changes to your medications? Blood pressure drugs, Sedatives (benzodiazepines), Antidepressants Allergies |
| FAMILY HISTORY | Arrhythmias, Stroke, MI |
| SOCIAL HISTORY | Alcohol and smoking Driving – do they still drive? What effect has the fall had on their life, ADLs, confidence etc. Who is at home? Are there stairs in their home? Any modifications required  thinking about safe to discharge |

## Slide 13
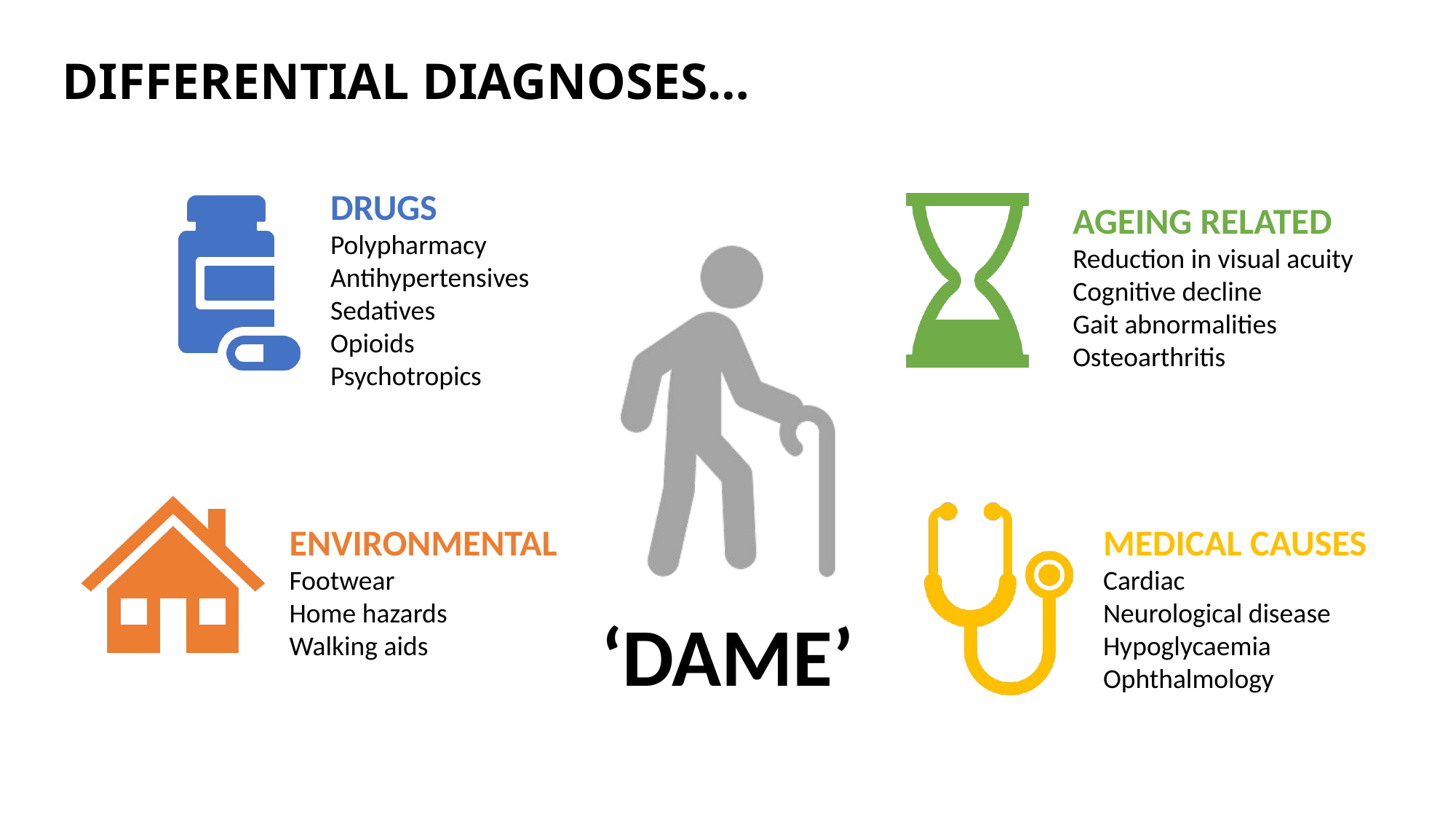

# DIFFERENTIAL DIAGNOSES…
DRUGS
Polypharmacy
Antihypertensives
Sedatives
Opioids
Psychotropics
AGEING RELATED
Reduction in visual acuity
Cognitive decline
Gait abnormalities
Osteoarthritis
ENVIRONMENTAL
Footwear
Home hazards
Walking aids
MEDICAL CAUSES
Cardiac
Neurological disease
Hypoglycaemia
Ophthalmology
‘DAME’

## Slide 14
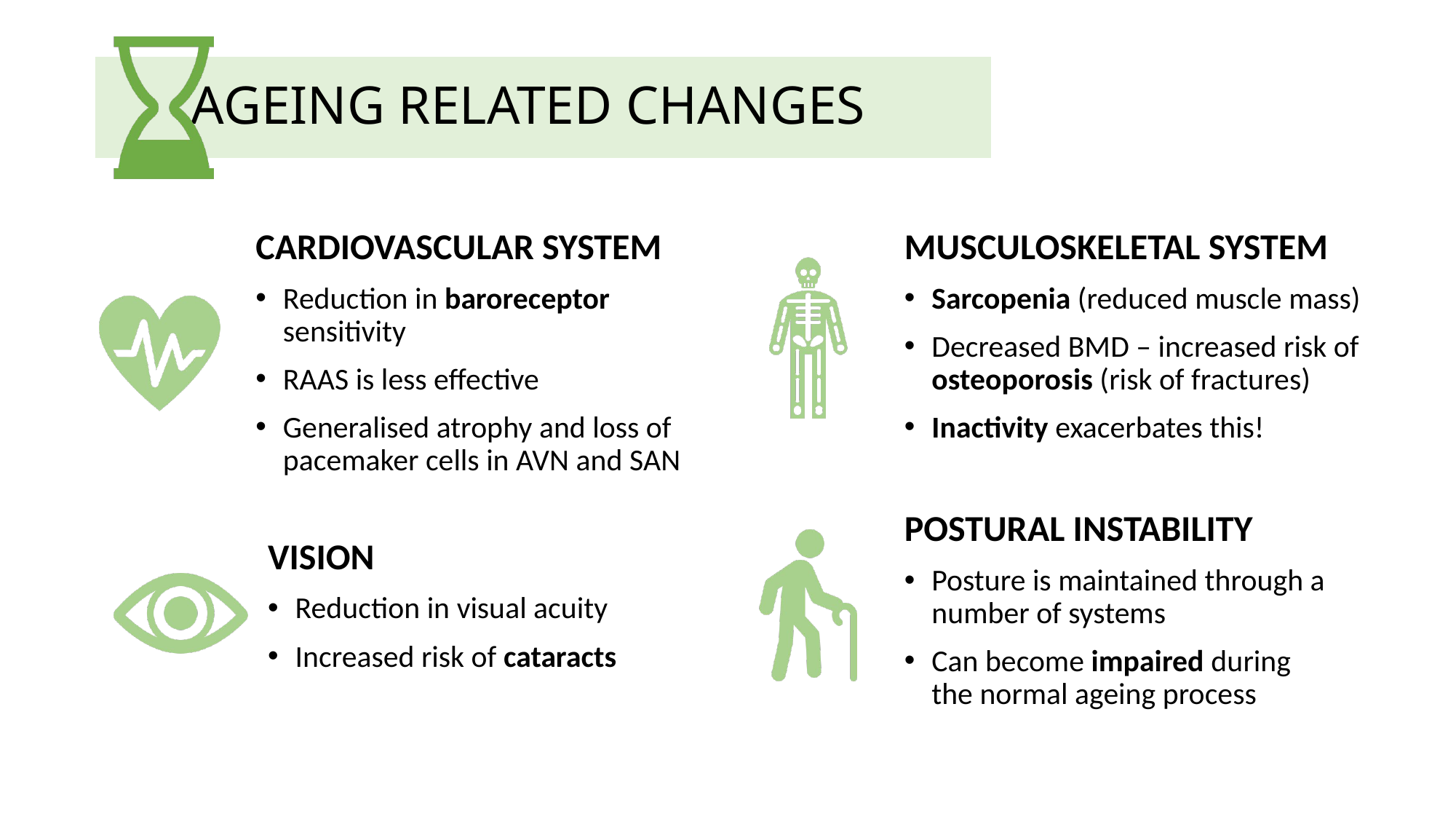

# AGEING RELATED CHANGES
CARDIOVASCULAR SYSTEM
Reduction in baroreceptor sensitivity
RAAS is less effective
Generalised atrophy and loss of pacemaker cells in AVN and SAN
MUSCULOSKELETAL SYSTEM
Sarcopenia (reduced muscle mass)
Decreased BMD – increased risk of osteoporosis (risk of fractures)
Inactivity exacerbates this!
POSTURAL INSTABILITY
Posture is maintained through a number of systems
Can become impaired during the normal ageing process
VISION
Reduction in visual acuity
Increased risk of cataracts

## Slide 15
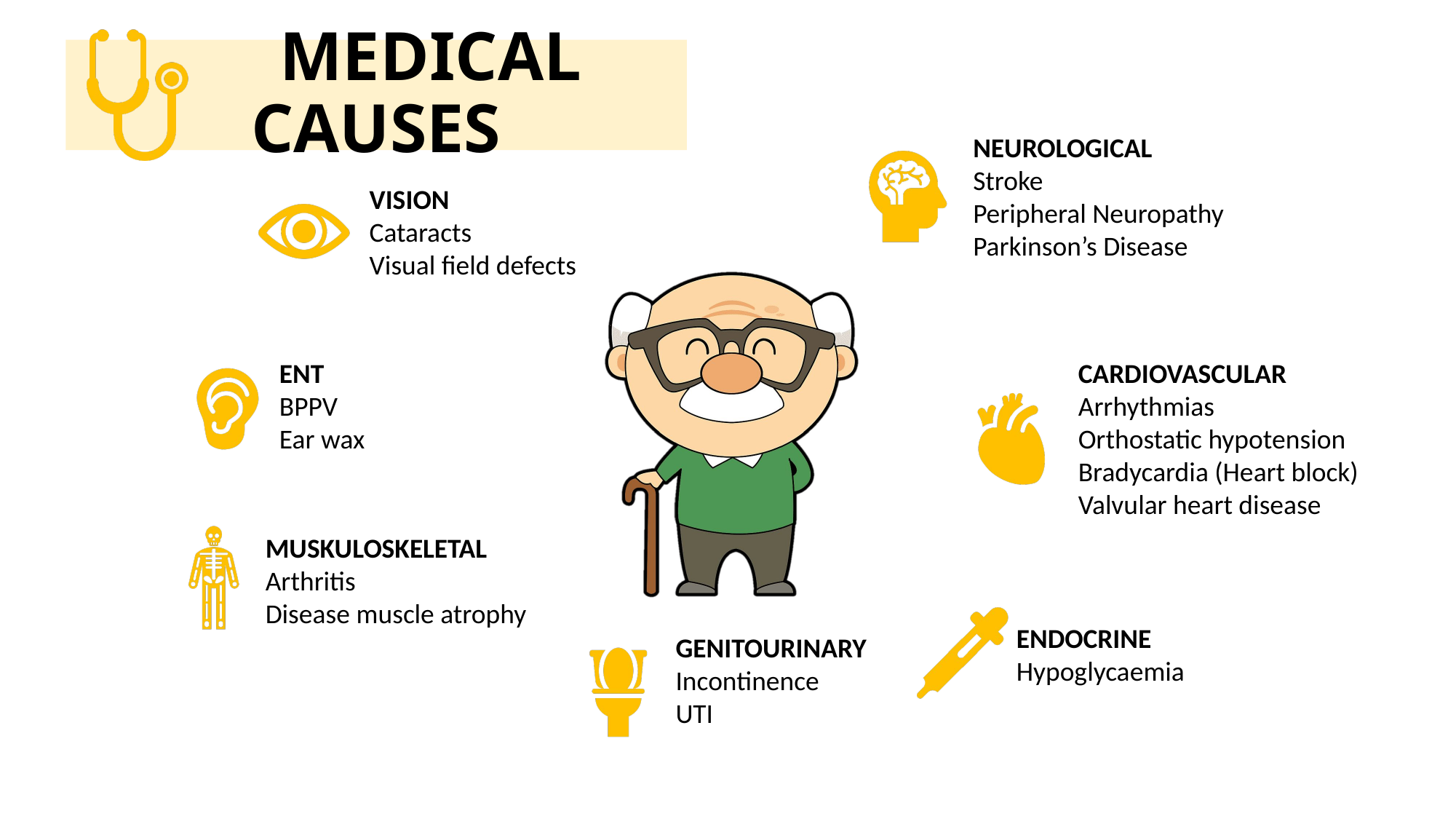

# MEDICAL CAUSES
NEUROLOGICAL
Stroke
Peripheral Neuropathy
Parkinson’s Disease
VISION
Cataracts
Visual field defects
ENT
BPPV
Ear wax
CARDIOVASCULAR
Arrhythmias
Orthostatic hypotension
Bradycardia (Heart block)
Valvular heart disease
MUSKULOSKELETAL
Arthritis
Disease muscle atrophy
ENDOCRINE
Hypoglycaemia
GENITOURINARY
Incontinence
UTI

## Slide 16
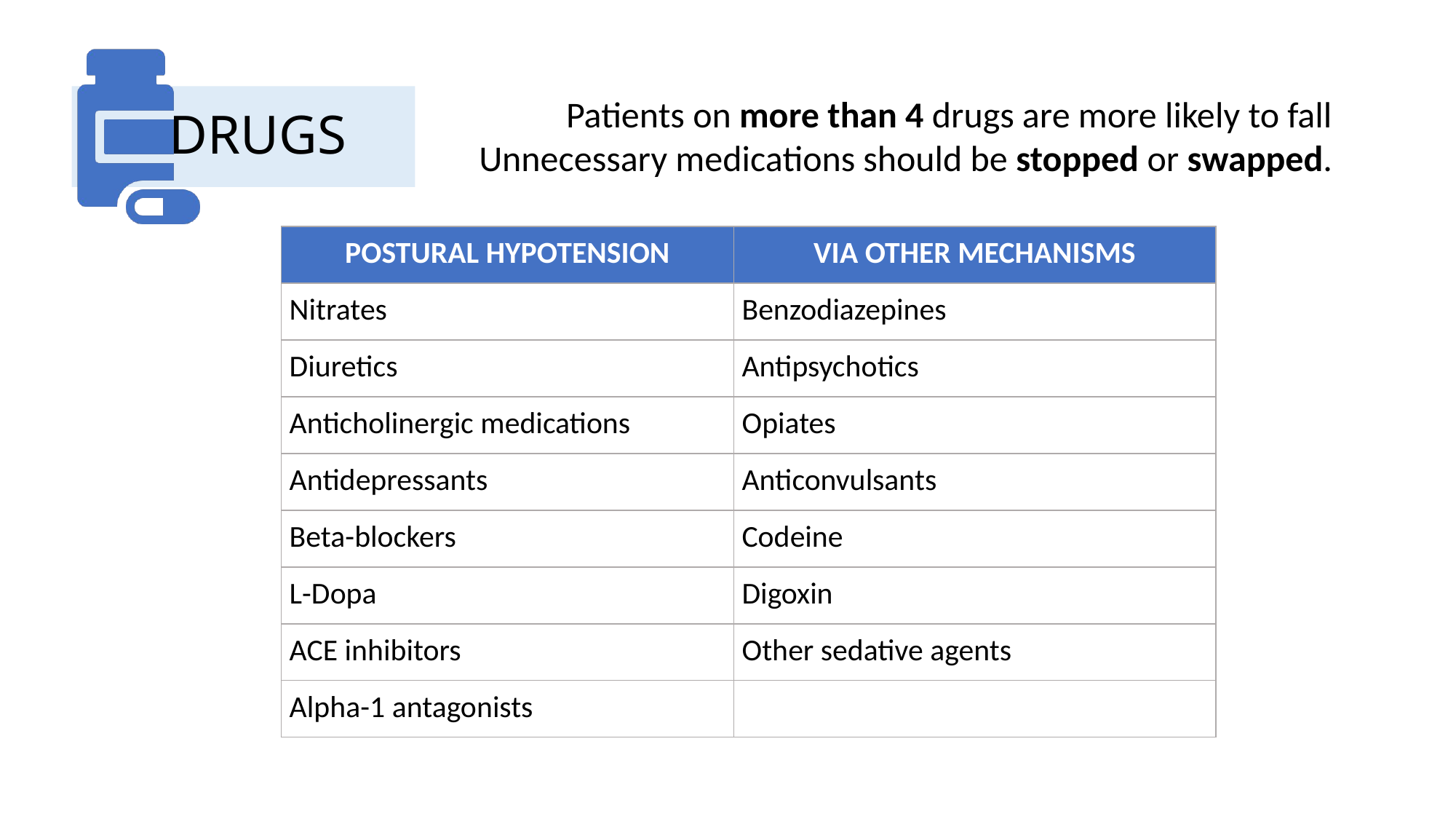

DRUGS
Patients on more than 4 drugs are more likely to fall
Unnecessary medications should be stopped or swapped.
| POSTURAL HYPOTENSION | VIA OTHER MECHANISMS |
| --- | --- |
| Nitrates | Benzodiazepines |
| Diuretics | Antipsychotics |
| Anticholinergic medications | Opiates |
| Antidepressants | Anticonvulsants |
| Beta-blockers | Codeine |
| L-Dopa | Digoxin |
| ACE inhibitors | Other sedative agents |
| Alpha-1 antagonists | |

## Slide 17
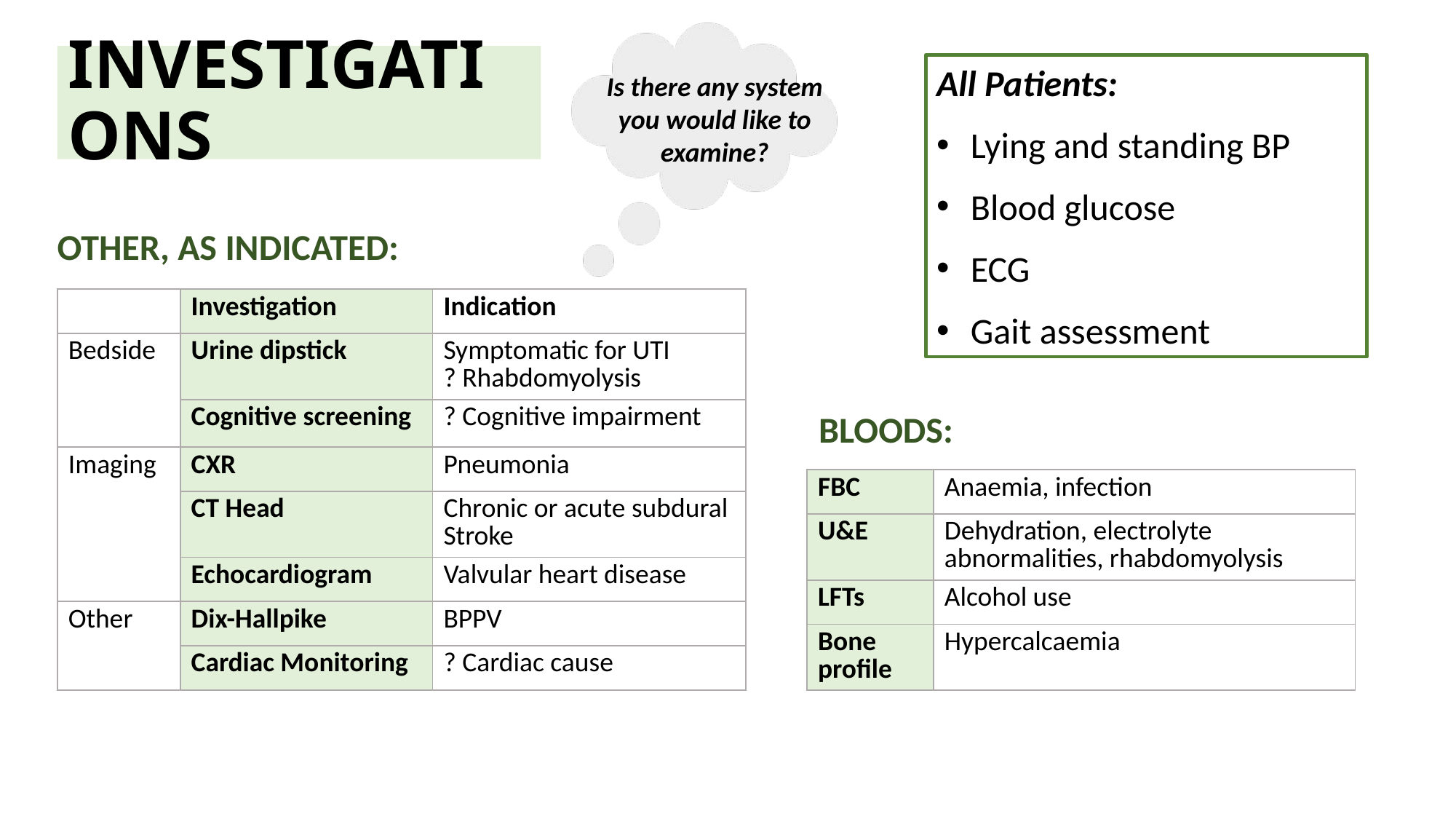

# INVESTIGATIONS
All Patients:
Lying and standing BP
Blood glucose
ECG
Gait assessment
Is there any system you would like to examine?
OTHER, AS INDICATED:
| | Investigation | Indication |
| --- | --- | --- |
| Bedside | Urine dipstick | Symptomatic for UTI ? Rhabdomyolysis |
| | Cognitive screening | ? Cognitive impairment |
| Imaging | CXR | Pneumonia |
| | CT Head | Chronic or acute subdural Stroke |
| | Echocardiogram | Valvular heart disease |
| Other | Dix-Hallpike | BPPV |
| | Cardiac Monitoring | ? Cardiac cause |
BLOODS:
| FBC | Anaemia, infection |
| --- | --- |
| U&E | Dehydration, electrolyte abnormalities, rhabdomyolysis |
| LFTs | Alcohol use |
| Bone profile | Hypercalcaemia |

## Slide 18
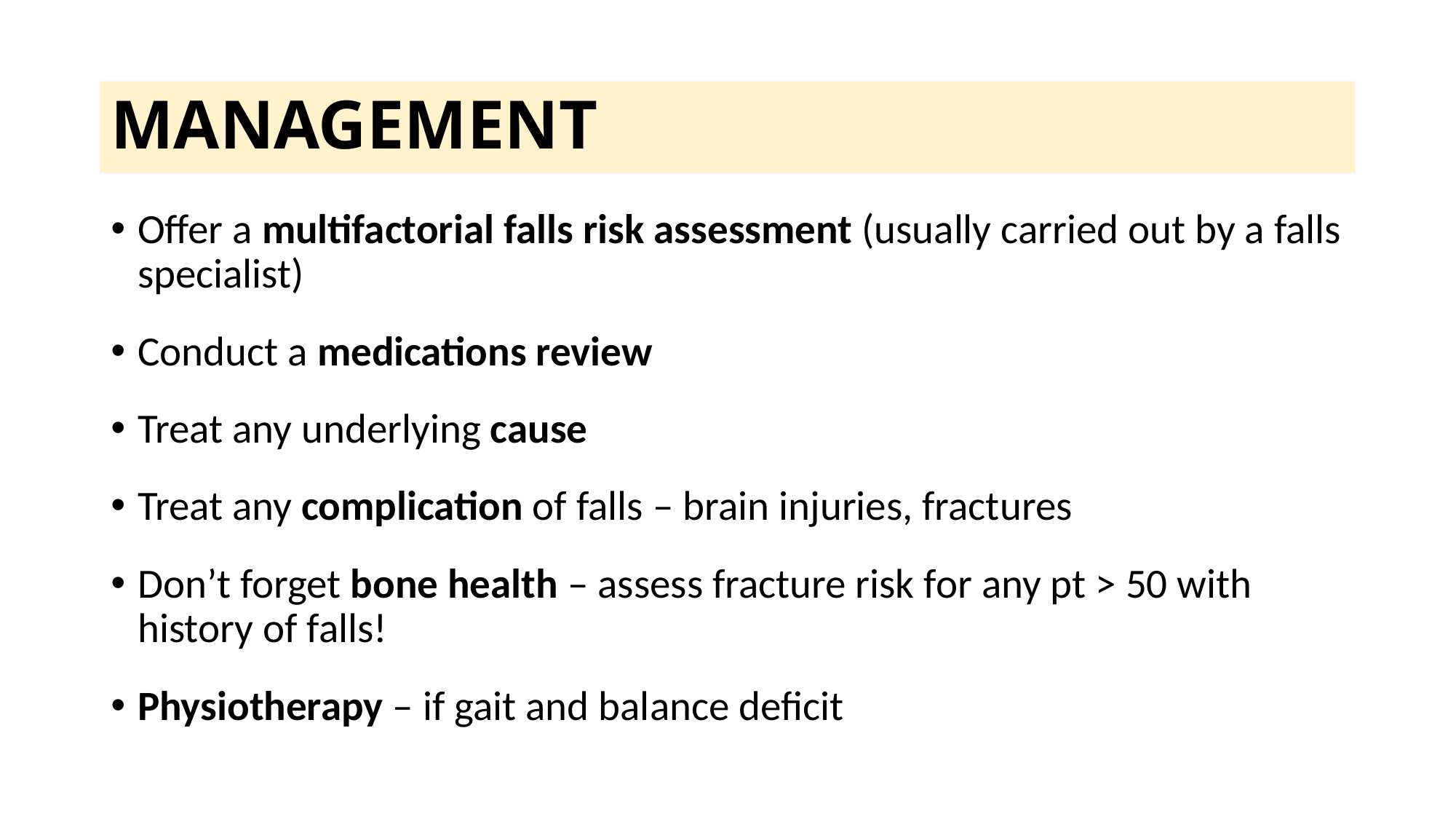

# MANAGEMENT
Offer a multifactorial falls risk assessment (usually carried out by a falls specialist)
Conduct a medications review
Treat any underlying cause
Treat any complication of falls – brain injuries, fractures
Don’t forget bone health – assess fracture risk for any pt > 50 with history of falls!
Physiotherapy – if gait and balance deficit

## Slide 19
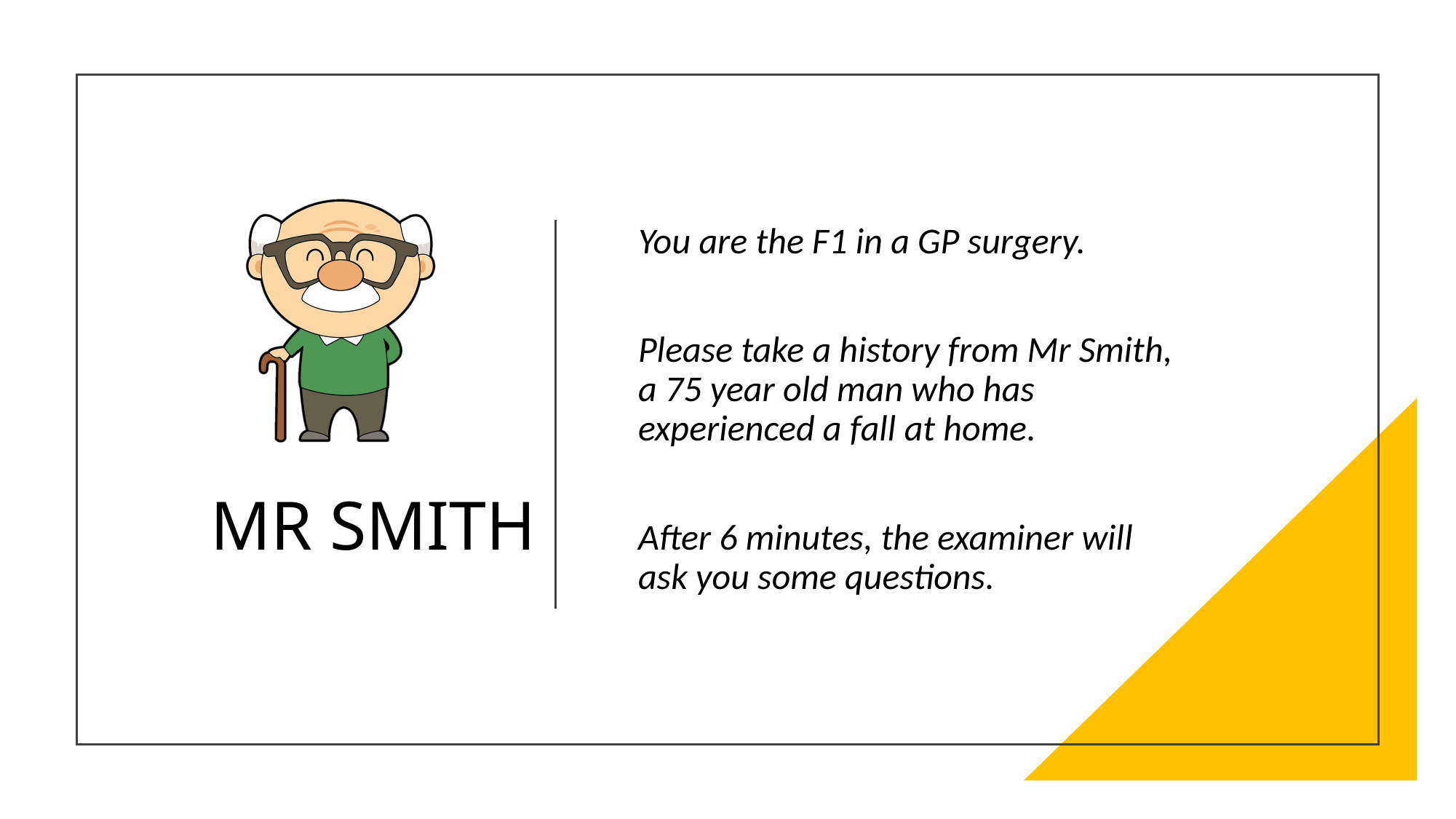

You are the F1 in a GP surgery.
Please take a history from Mr Smith, a 75 year old man who has experienced a fall at home.
After 6 minutes, the examiner will ask you some questions.
# MR SMITH

## Slide 20
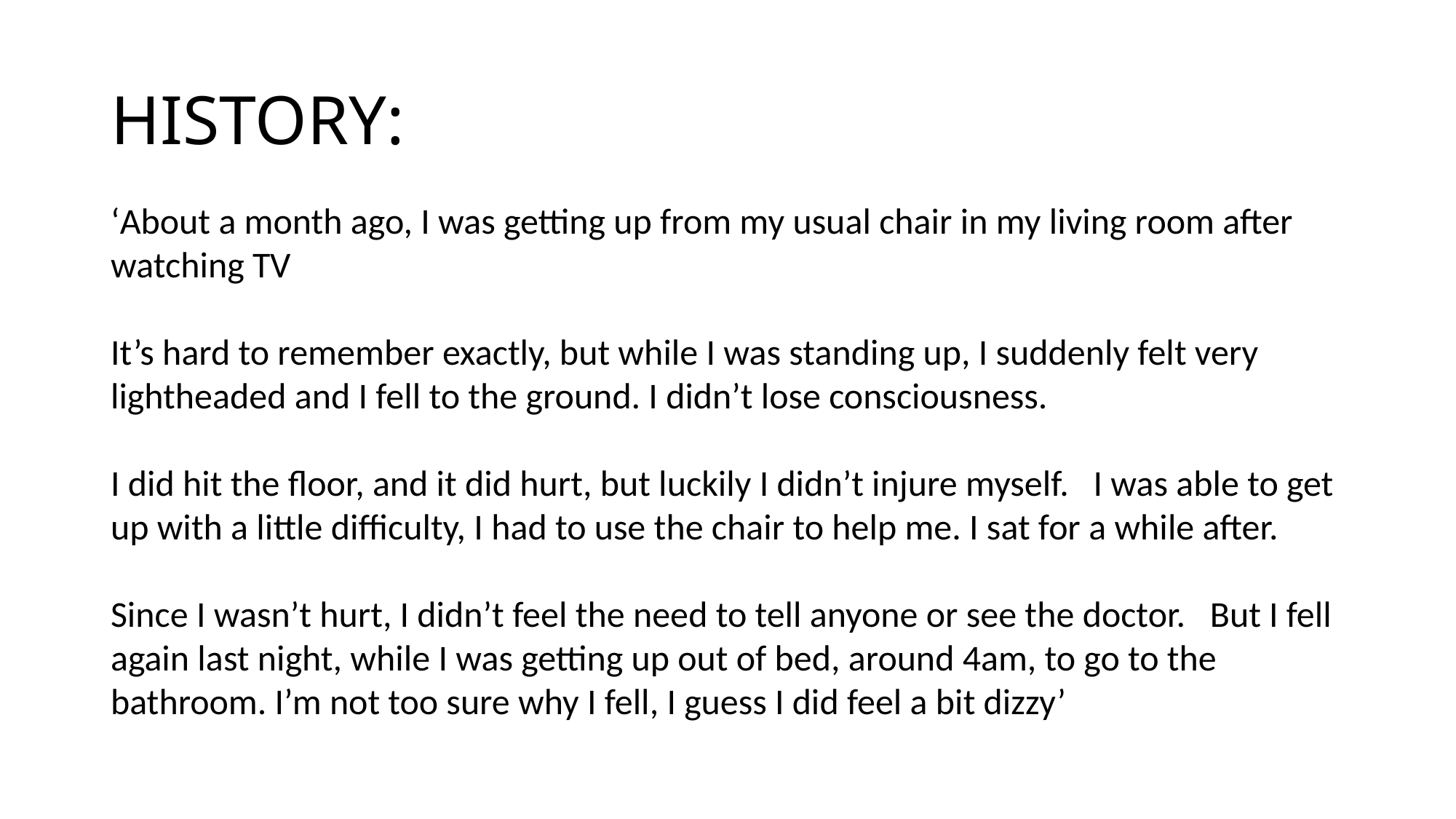

# HISTORY:
‘About a month ago, I was getting up from my usual chair in my living room after watching TV
It’s hard to remember exactly, but while I was standing up, I suddenly felt very lightheaded and I fell to the ground. I didn’t lose consciousness.
I did hit the floor, and it did hurt, but luckily I didn’t injure myself. I was able to get up with a little difficulty, I had to use the chair to help me. I sat for a while after.
Since I wasn’t hurt, I didn’t feel the need to tell anyone or see the doctor. But I fell again last night, while I was getting up out of bed, around 4am, to go to the bathroom. I’m not too sure why I fell, I guess I did feel a bit dizzy’

## Slide 21
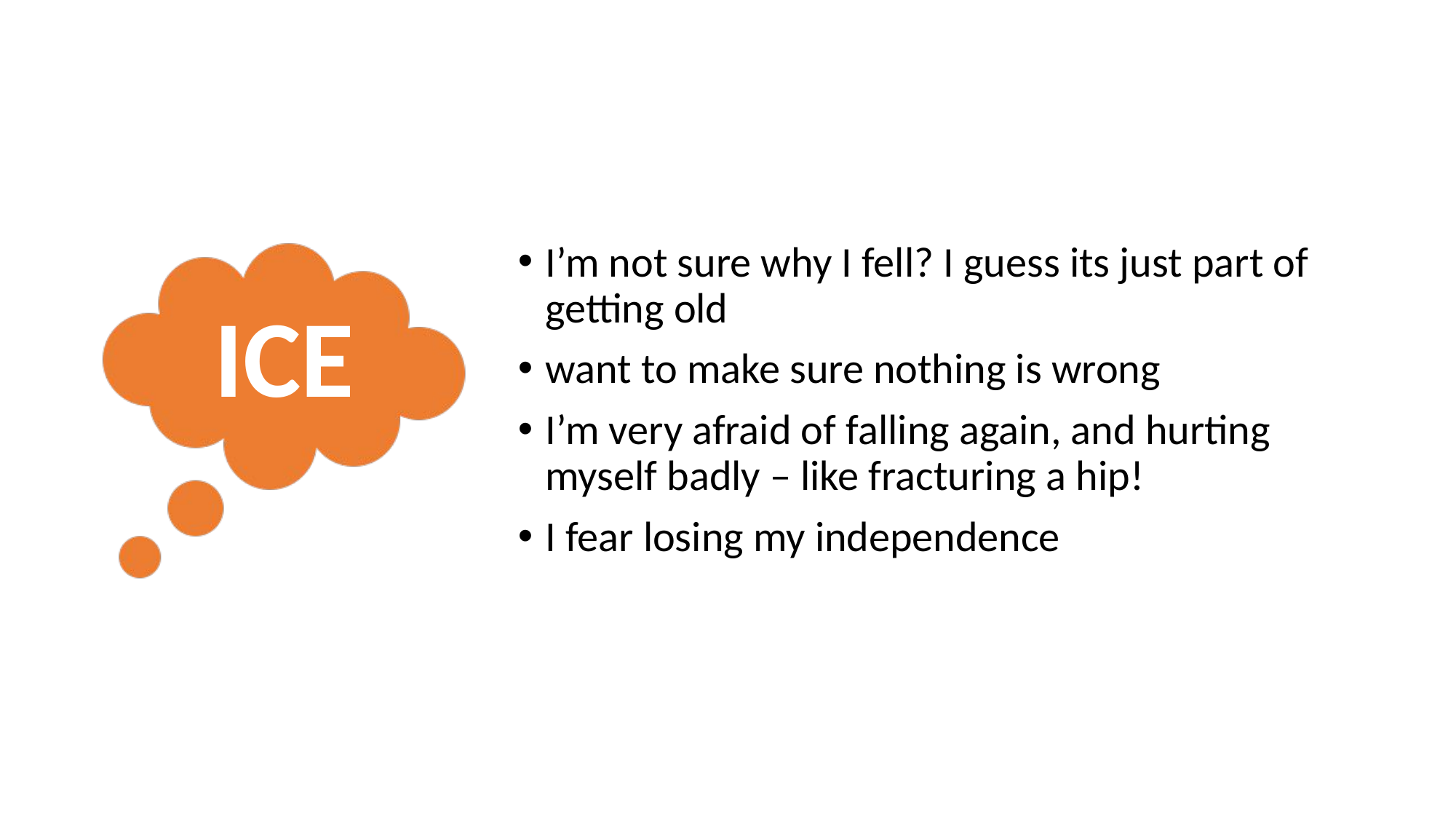

I’m not sure why I fell? I guess its just part of getting old
want to make sure nothing is wrong
I’m very afraid of falling again, and hurting myself badly – like fracturing a hip!
I fear losing my independence
ICE

## Slide 22
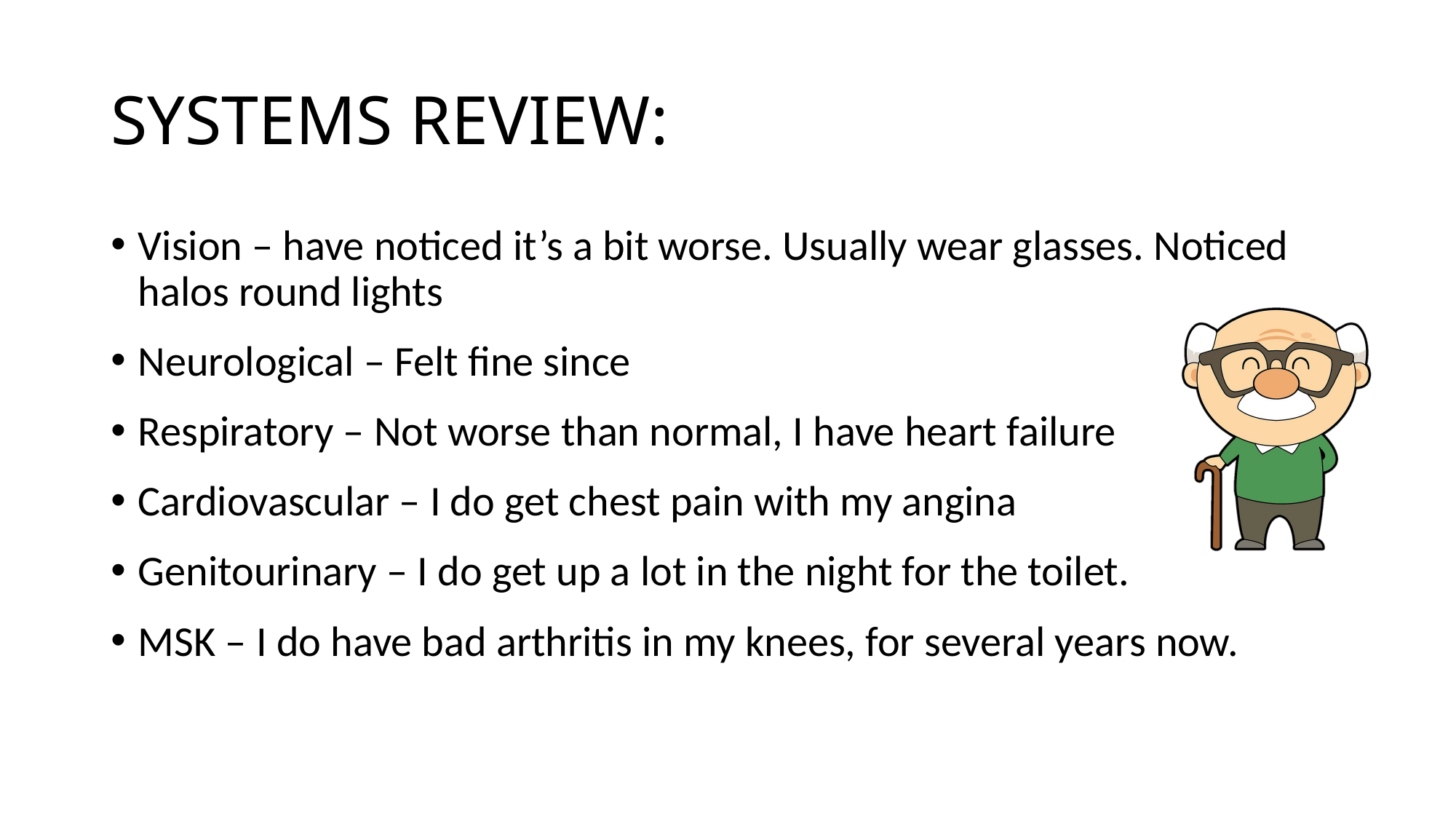

# SYSTEMS REVIEW:
Vision – have noticed it’s a bit worse. Usually wear glasses. Noticed halos round lights
Neurological – Felt fine since
Respiratory – Not worse than normal, I have heart failure
Cardiovascular – I do get chest pain with my angina
Genitourinary – I do get up a lot in the night for the toilet.
MSK – I do have bad arthritis in my knees, for several years now.

## Slide 23
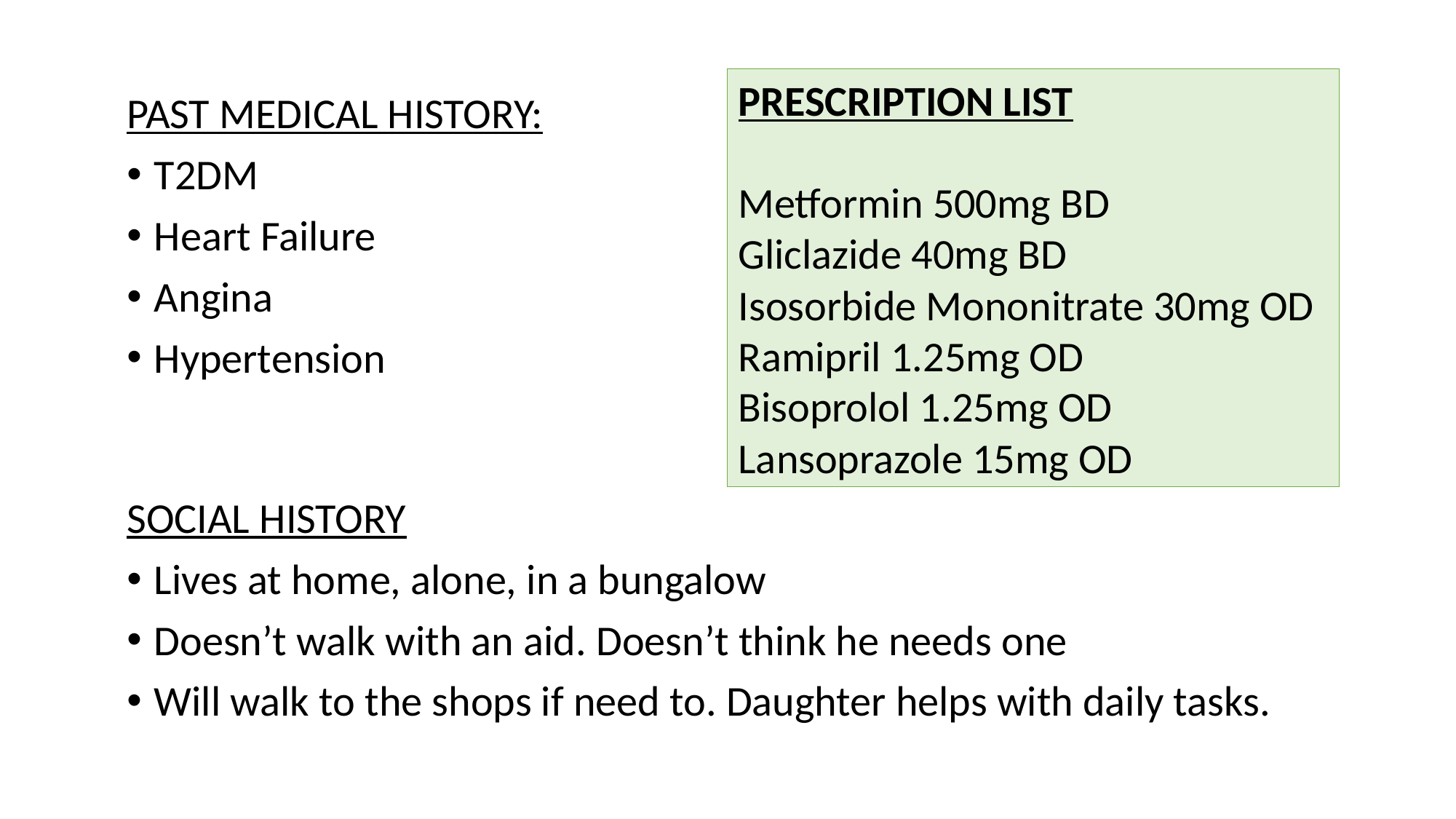

PRESCRIPTION LIST
Metformin 500mg BD
Gliclazide 40mg BD
Isosorbide Mononitrate 30mg OD
Ramipril 1.25mg OD
Bisoprolol 1.25mg OD
Lansoprazole 15mg OD
PAST MEDICAL HISTORY:
T2DM
Heart Failure
Angina
Hypertension
SOCIAL HISTORY
Lives at home, alone, in a bungalow
Doesn’t walk with an aid. Doesn’t think he needs one
Will walk to the shops if need to. Daughter helps with daily tasks.

## Slide 24
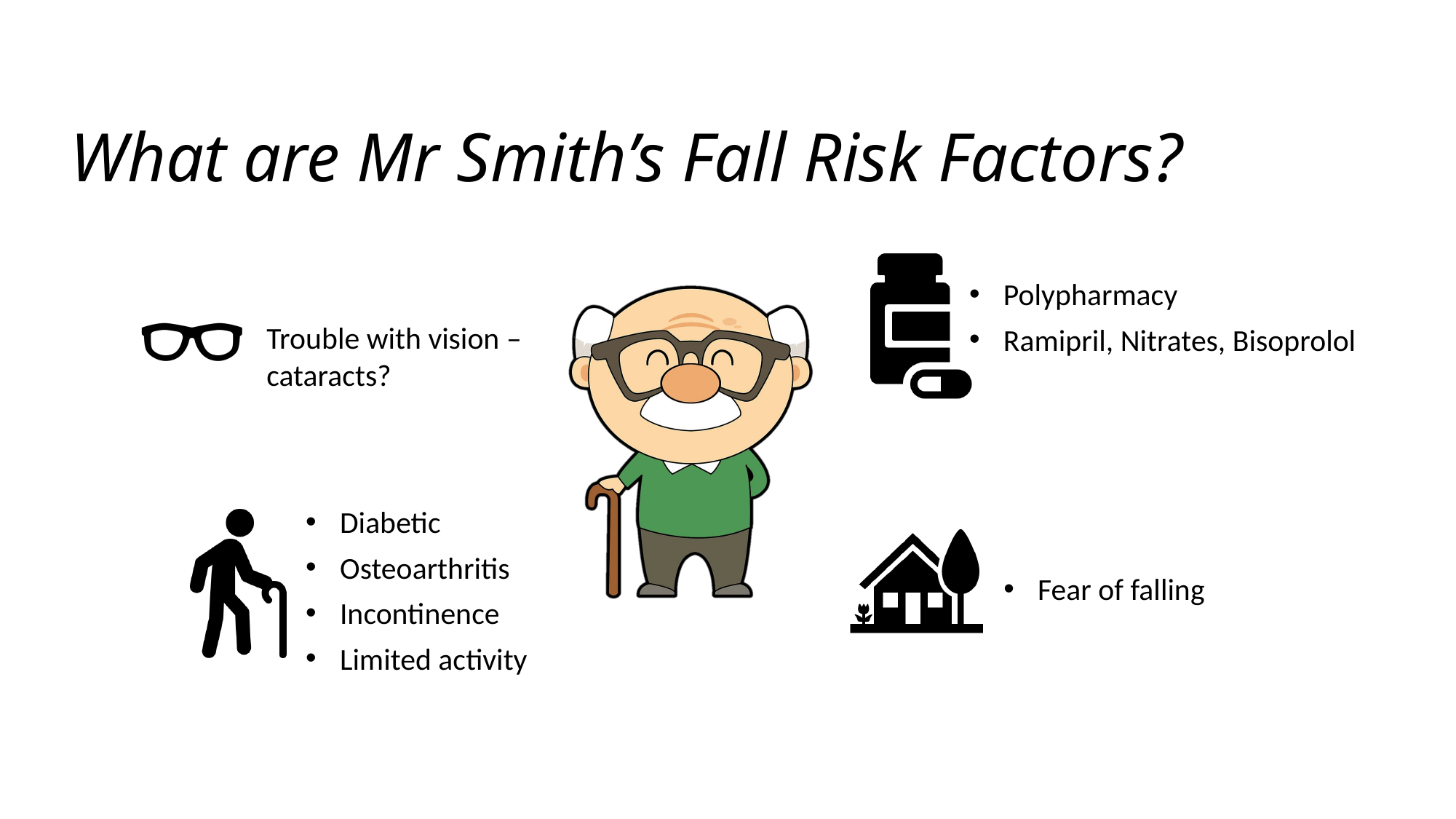

# What are Mr Smith’s Fall Risk Factors?
Polypharmacy
Ramipril, Nitrates, Bisoprolol
Trouble with vision – cataracts?
Diabetic
Osteoarthritis
Incontinence
Limited activity
Fear of falling

## Slide 25
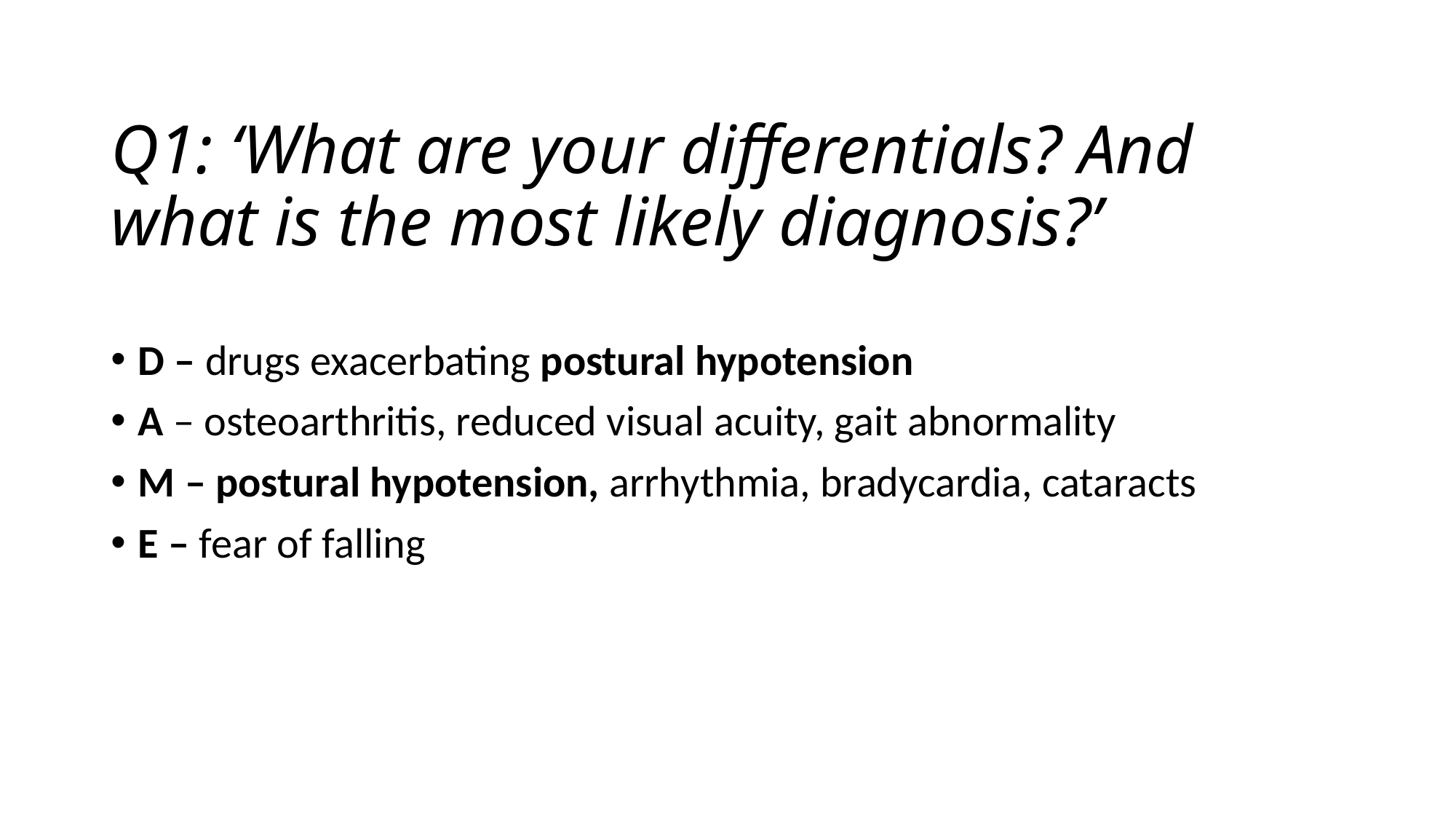

# Q1: ‘What are your differentials? And what is the most likely diagnosis?’
D – drugs exacerbating postural hypotension
A – osteoarthritis, reduced visual acuity, gait abnormality
M – postural hypotension, arrhythmia, bradycardia, cataracts
E – fear of falling

## Slide 26
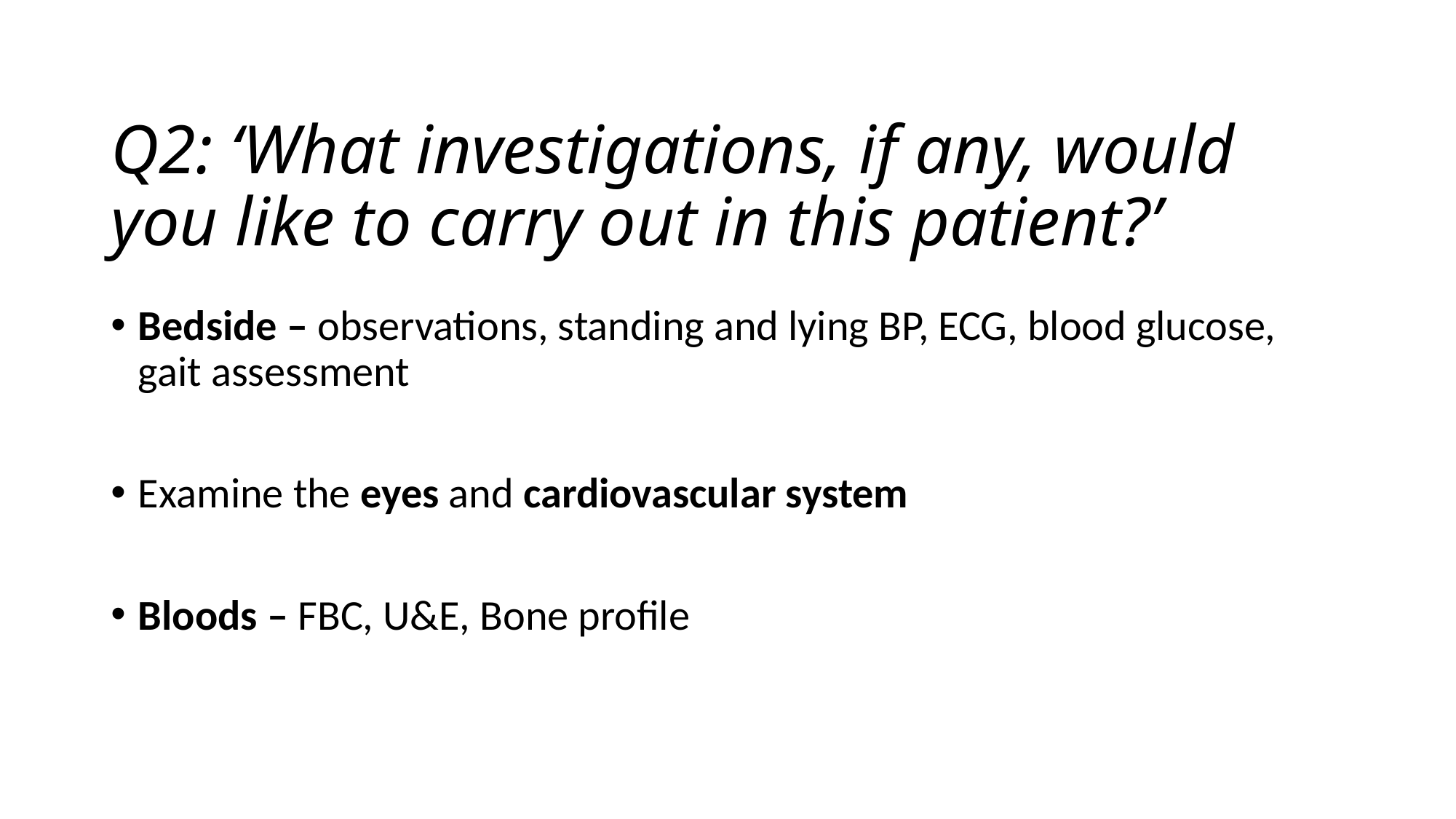

# Q2: ‘What investigations, if any, would you like to carry out in this patient?’
Bedside – observations, standing and lying BP, ECG, blood glucose, gait assessment
Examine the eyes and cardiovascular system
Bloods – FBC, U&E, Bone profile

## Slide 27
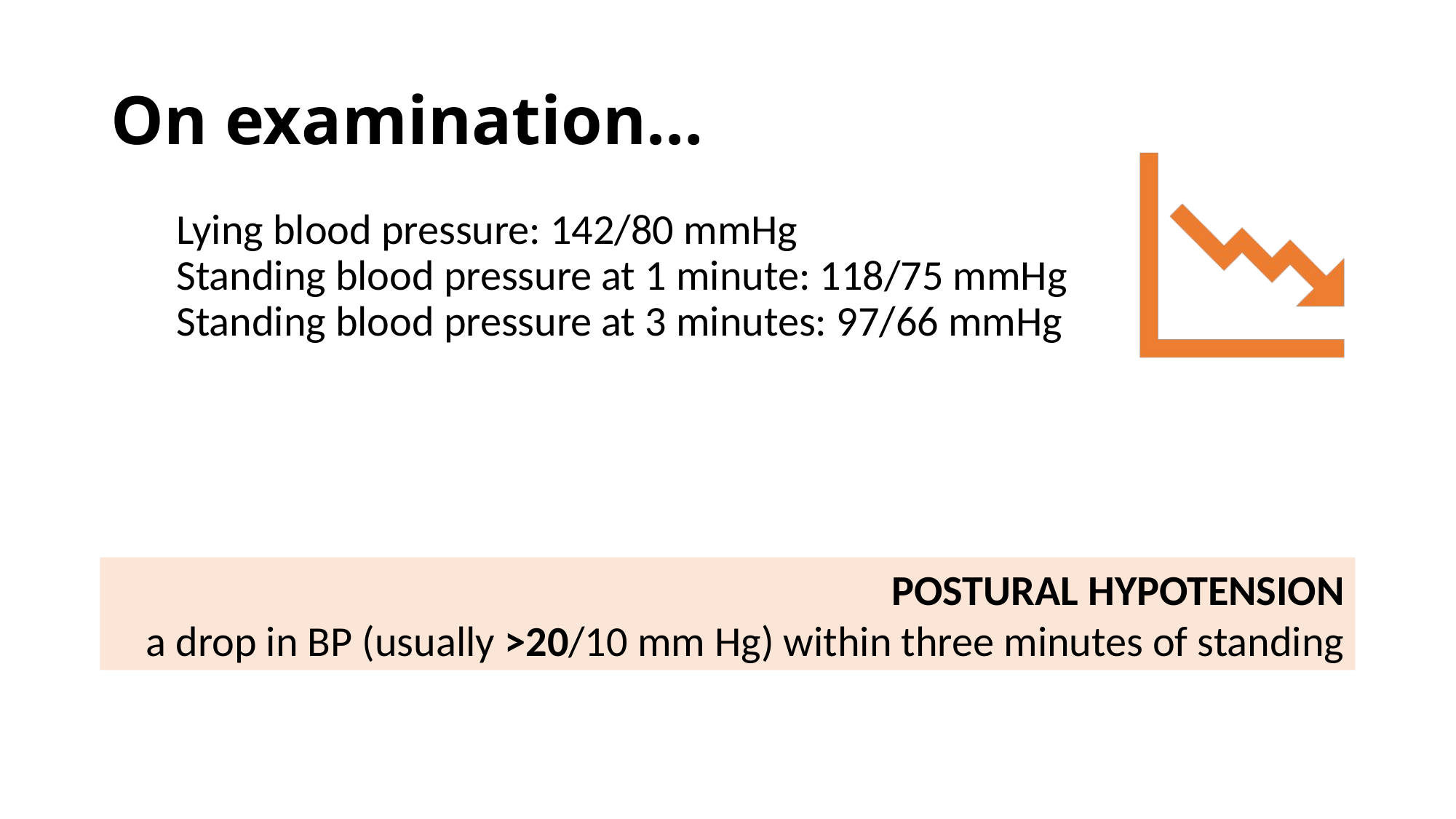

# On examination…
Lying blood pressure: 142/80 mmHg 
Standing blood pressure at 1 minute: 118/75 mmHg
Standing blood pressure at 3 minutes: 97/66 mmHg
 POSTURAL HYPOTENSION
a drop in BP (usually >20/10 mm Hg) within three minutes of standing

## Slide 28
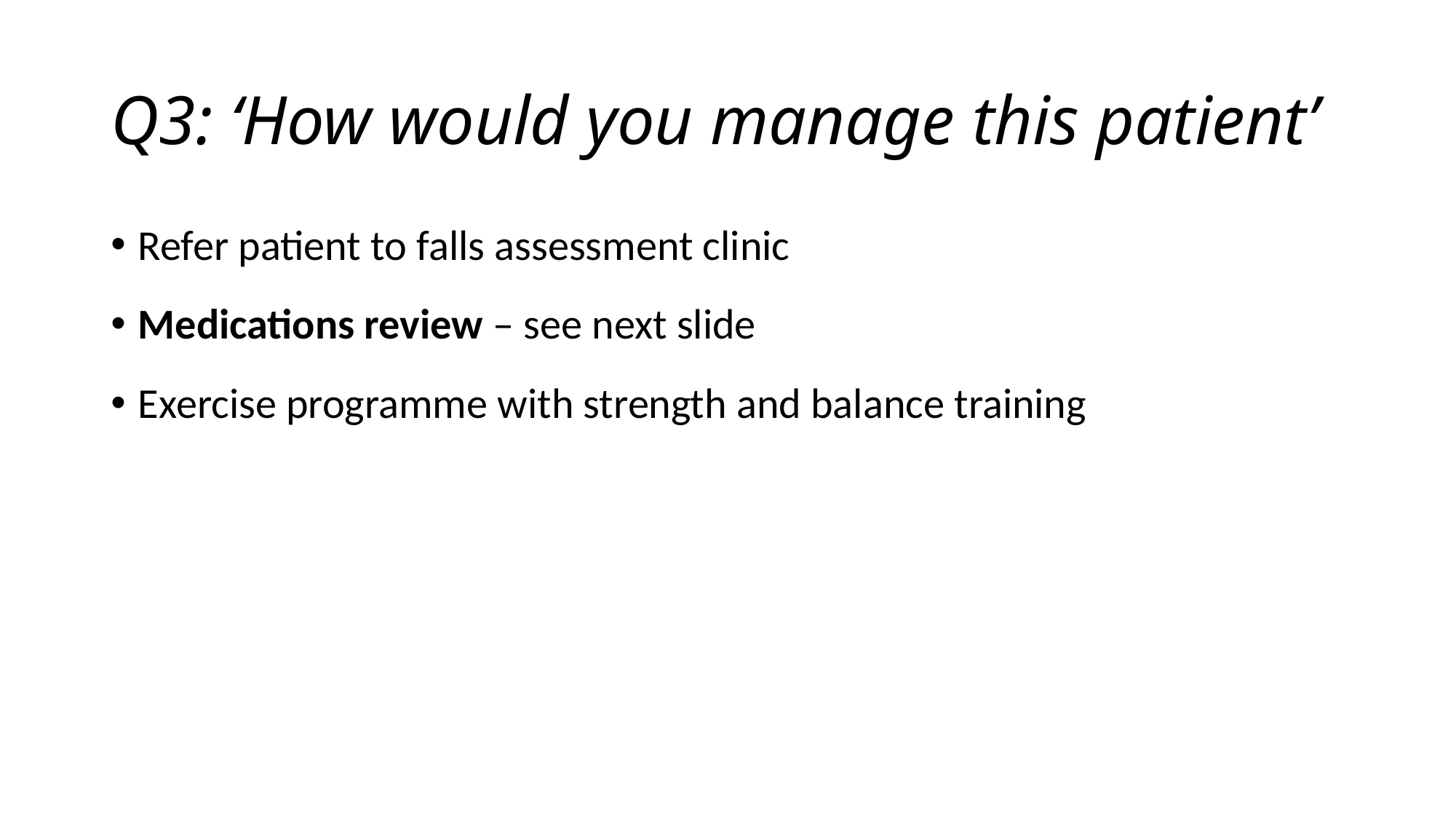

# Q3: ‘How would you manage this patient’
Refer patient to falls assessment clinic
Medications review – see next slide
Exercise programme with strength and balance training

## Slide 29
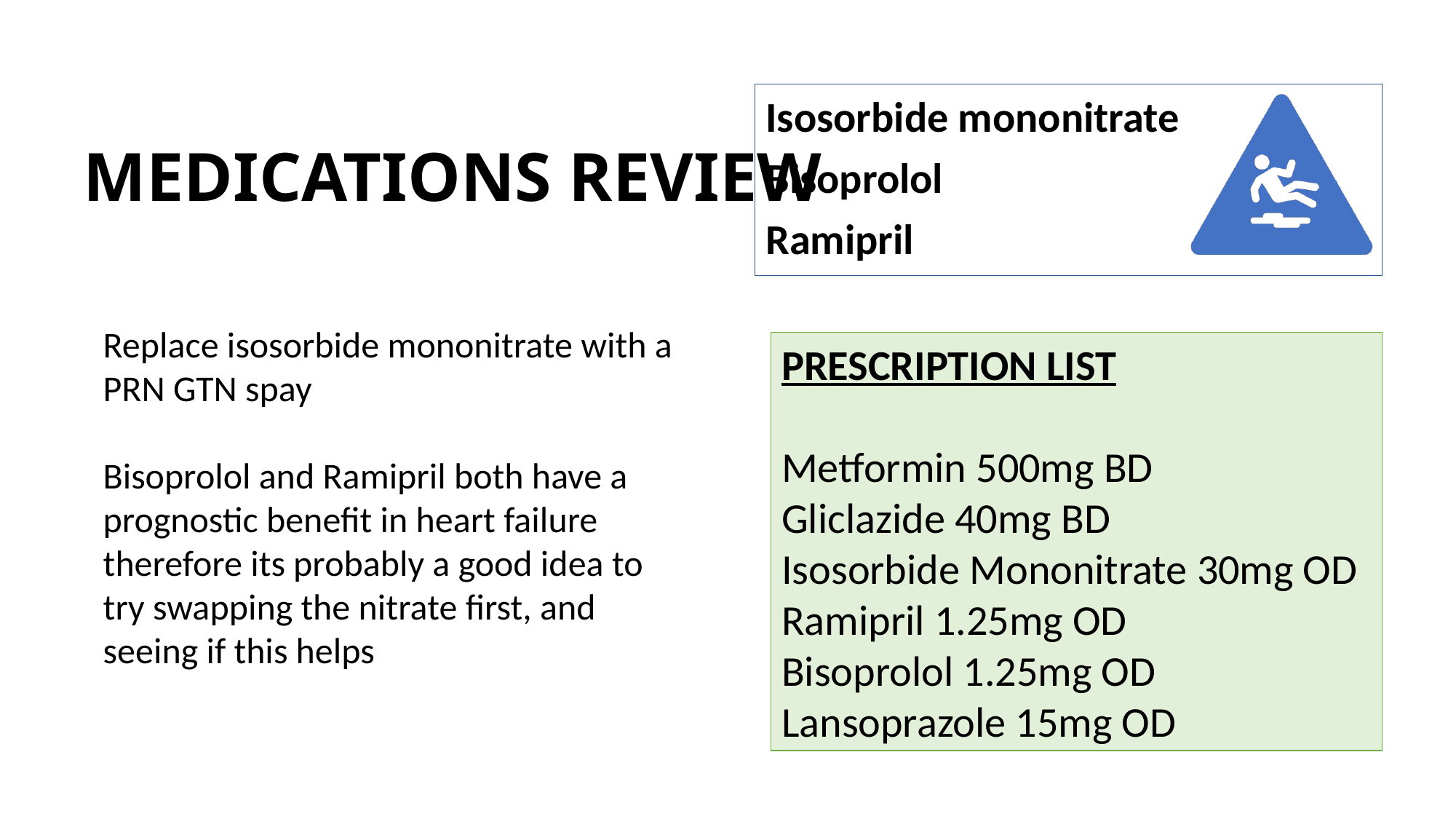

Isosorbide mononitrate
Bisoprolol
Ramipril
# MEDICATIONS REVIEW
Replace isosorbide mononitrate with a PRN GTN spay
Bisoprolol and Ramipril both have a prognostic benefit in heart failure therefore its probably a good idea to try swapping the nitrate first, and seeing if this helps
PRESCRIPTION LIST
Metformin 500mg BD
Gliclazide 40mg BD
Isosorbide Mononitrate 30mg OD
Ramipril 1.25mg OD
Bisoprolol 1.25mg OD
Lansoprazole 15mg OD

## Slide 30
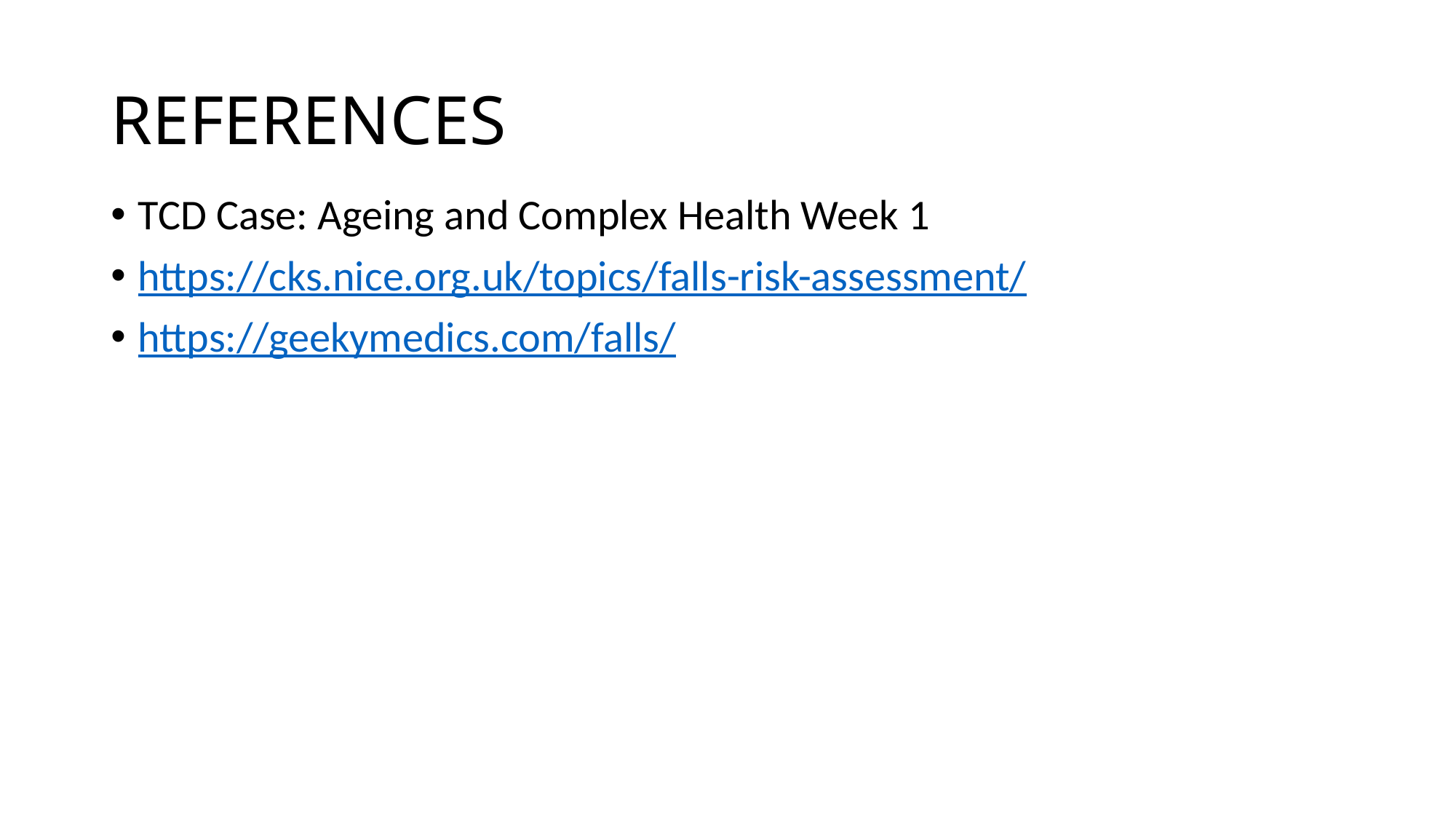

# REFERENCES
TCD Case: Ageing and Complex Health Week 1
https://cks.nice.org.uk/topics/falls-risk-assessment/
https://geekymedics.com/falls/
